# Advancing Advocacy: Implementation of a Child Health Advocacy Curriculum in a Pediatrics Residency Program

**DOI:** 10.15766/mep_2374-8265.10882

**Published:** 2020-02-14

**Authors:** Amara Majeed, Heather Newton, Arnold Mahesan, Turaj Vazifedan, Dana Ramirez

**Affiliations:** 1 Clinical Fellow, Department of Pediatric Cardiology, Harvard Medical School; 2 Instructor, GME Programs, Eastern Virginia Medical School; 3 Clinical Fellow, Department of Reproductive Endocrinology and Infertility, Eastern Virginia Medical School; 4 Instructor, Department of Pediatrics, Eastern Virginia Medical School; 5 Associate Professor, Department of Pediatrics, Eastern Virginia Medical School

**Keywords:** Child Health Advocacy Curriculum, Child Health, Advocacy, Case-Based Learning, Focus Groups/Interviews, Program Evaluation, Simulation

## Abstract

**Introduction:**

ACGME program requirements for graduate medical education state that pediatric residency programs should include elements of child advocacy education. Finding readily available, easily implementable advocacy curricula for pediatric residency programs is challenging. We conducted a generalized curricular needs assessment via literature review and a targeted needs assessment with health care providers and advocacy leaders and developed and implemented a child health advocacy curriculum in a pediatrics residency program.

**Methods:**

Delivered across 9 months, the curriculum included three components: electronic resources, didactic sessions, and interactive workshops aimed at developing advocacy skills in the context of pressing child health issues. The learner audience was PGY 1 through PGY 4. The curriculum was evaluated using pre- and postcurriculum surveys.

**Results:**

Our curriculum advanced child advocacy locally by establishing partnerships with state and federal American Academy of Pediatrics and pediatric residency programs, teaching residents to generate advocacy action plans, and implanting a longitudinal advocacy curriculum in the residency program. Sixty-four of 70 residents participated in the curriculum: 33% were PGY 1, 31% were PGY 2, 30% were PGY 3, and 6% were PGY 4. Pre- and postcurriculum surveys demonstrated improved knowledge of and comfort level with advocacy after curriculum completion.

**Discussion:**

Child advocacy teaching improved resident and faculty awareness about child health issues in the community, as well as understanding of pathways to advocate for child health. The curriculum is reproducible and feasible and can assist other institutions to develop advocacy education and skill development programs.

## Educational Objectives

After completing this advocacy curriculum, trainees will be able to:

1. Identify key child health issues in their community.
2. Demonstrate improved knowledge of state and federal legislation processes and of current state-specific legislation regarding child health.
3. Communicate with state and federal representatives regarding a child health issue.
4. Express opinions via communication with child health organizations and through op-ed writing.
5. Design and present a state-specific action plan to advocate to a legislative body on a child health issue.
6. Demonstrate an improved comfort level with child health advocacy as a component of their future careers as pediatricians.

## Introduction

Currently, the ACGME specifies that pediatric residency should include five educational units of ambulatory experiences, including elements of community pediatrics and child advocacy.^1^ Health advocacy comprises advocacy at the individual, community, state, and national levels.^2^ There has been a growing focus on child health legislative activities in pediatric residency programs, including greater resident involvement in providing legislative testimony.^3^ Recently, pilot innovative curricula and independent curricula have been developed for community-level advocacy.^4–7^ Tools have been developed to link advocacy training to resident competence^8^ and for community-based project planning.^9^ However, it can be a challenge for pediatric programs to find easily implemented and locally relevant curricula encompassing various levels of health advocacy.

To better accomplish this goal of training future pediatricians to be capable of participating effectively in community-, state-, and national-level child health advocacy, we developed a relevant and easily implementable curriculum in our pediatric residency program. Available pediatric curricula have focused on generalized advocacy training models^4^ and assessment tools.^8,9^ We add to the existing literature by describing in depth the development, implementation, and results of a locally relevant tested framework to help educate the next generation of pediatricians to advocate for their patients in a continuously evolving political advocacy landscape. We aimed to empower residents to be effective advocates by improving resident knowledge regarding state and federal children's health legislation and legislative processes and by teaching specific skills such as op-ed writing and the art of negotiation during child health advocacy meetings with federal and state representatives.

Here, we describe the process of development of this curriculum, as well as results and outputs from the pilot within our residency program, and share our curricular framework and resources that could be utilized by other programs. Our hope is that by outlining the stepwise approach we took in development, implementation, and evaluation, as well as by sharing the end product as a resource, we can help residency educators looking to implement locally relevant advocacy curricula at their institutions.

## Methods

We structured our curriculum development using Kern's six steps.^10^

### Step 1: Problem Identification and General Needs Assessment

We conducted an extensive literature search and identified a paucity of locally relevant curricula to effectively teach pediatric residents the art of community-, state-, and national-level child health advocacy. Out of 200 articles reviewed about advocacy in medicine, only seven introduced advocacy curricula for pediatric trainees. With support from an American Academy of Pediatrics (AAP) Community Pediatrics Training Initiative grant to develop a curriculum, we attended the AAP Legislative Conference in Washington, DC, in April 2016. This conference further highlighted gaps in pediatric trainee advocacy teaching and taught attendees basic advocacy skills via workshops, seminars, and meetings with federal representatives. Skills acquired were used for the layout of the sessions of our curriculum.

### Step 2: Targeted Needs Assessment

We formed a workgroup for a targeted needs assessment at our hospital, Children's Hospital of the King's Daughters. The workgroup included local and national child advocacy leaders from the hospital, advocacy representatives from the Eastern Virginia Medical School, the director of graduate medical education, and representatives of our state AAP chapter. Furthermore, residents were given a precurriculum survey that revealed resident gaps in advocacy knowledge and skills. Thirty-seven percent of residents responded that they were not aware of pertinent local child health issues, 58% indicated that they were not familiar with advocacy resources, and 43% felt intimidated by the legislative process. The workgroup guided incorporation of high-yield advocacy skills into the curriculum based on the AAP Legislative Conference and resident survey results. For our interactive workshops, we chose to focus on three child health issues of local and national importance: pediatric nutrition, sudden infant death syndrome (SIDS), and child mental health. We also chose three important advocacy skills: communicating with state representatives, the art of negotiation, and op-ed writing.

### Step 3: Curriculum Goals and Objectives

Table 1 shows our objectives. Knowledge, attitude, and skills objectives were formulated after community and resident needs assessments and aligned with the pediatric milestones and ACGME competencies.

**Table 1.** Advocacy Curriculum Objectives

		Essential Program Component for Advocacy Training						
Competency	Objective	Community Engagement	Transferable Skills	Health Disparities	Bidirectional Learning	Experiential Learning	Interprofessional Approach	Reflection
Systems-Based Practice	1. Pediatric residents will learn how to access and utilize advocacy training resources via the residency webpage.		X					
	2. Residents will discuss current issues with point of view of local medical societies, American Academy of Pediatrics, and local representative's office.	X		X			X	X
Interpersonal and Communication Skills	3. Residents will accurately determine their state and federal representatives and understand how to contact them.						X	
	4. Residents will demonstrate that they have gained the skills necessary to be able to write op-eds, practice negotiations, and conduct child health advocacy meetings with their representatives.	X	X		X	X		X
	5. Residents will communicate effectively with patients, families, and the public, as appropriate, across a broad range of socioeconomic and cultural backgrounds.	X					X	
Practice-Based Learning and Improvement	6. Residents will identify at least three major child health issues in their state.		X					
	7. Residents will become familiar with existing state policies on sudden infant death syndrome, child mental health, and child nutrition, and they will be able to identify opportunities for improvement.		X	X				X
	8. Residents will have the opportunity to attend their state's or a nearby state's general assembly day, write an op-ed, or meet with a representative with assistance from course directors.	X	X		X	X	X	
Professionalism	9. Residents will conduct high standards of ethical behavior, which includes maintaining appropriate professional boundaries.						X	
	10. Residents will recognize a sense of duty and accountability to patients, society, and the profession.						X	X

### Step 4: Educational Strategies

The curriculum consisted of reading materials, didactic lectures, and interactive workshops. Our goal was twofold: advocacy education through reading and didactics and practical skill development through workshops, so as to fulfill both of these ACGME requirements. The learning methods chosen were selected primarily with a focus on ease of implementation in complex resident schedules. Since there was protected time for noon teaching sessions, didactic lectures and interactive workshop sessions were the learning forms most easily scheduled in these blocks and executed with available resources. Interactive workshops started with a 15-minute didactic describing a child health issue and existing state and national programs, as well as an introduction to an advocacy skill. A case scenario related to the child health issue was then introduced, and residents in each group practiced the focused advocacy skill. The groups were facilitated by local AAP members, faculty, and advocacy leaders. Role-play in particular was utilized, as the act of political advocacy necessarily involves interpersonal interactions and this was an excellent way to practice such interactions. Each group had reference materials and checklists for guidance about effectively discussing the case scenario. At the end of the workshop, each group shared key points of the experience.

### Step 5: Implementation

We administered the curriculum as a pilot from October 2016 to June 2017 with the intent to incorporate it permanently into the pediatric residency program curriculum after postimplementation evaluation. These lectures and workshops took place in the hour allotted to noon didactics for residents. Printouts of the case scenarios and PowerPoint presentations were distributed at each session. The average preparation time for each workshop was 1 month, involving two formal committee meetings prior to each workshop. Four facilitators were available during the role-play, and each facilitator supervised six to eight residents. The curriculum activities, also described in detail in the *Lectures* subsection, below, included the following:

1. Basics of advocacy and an introduction to the curriculum (facilitator: curriculum organizers):
  • Highlight the basics of advocacy, relevance to pediatricians, educational resources, and curriculum layout.
2. Overview of the legislative process and policy (facilitator: local legislative representative or advocacy leader):
  • Host a discussion on state child health including history, current laws, current challenges in child health policy, current views of state legislatures on child health policies, gaps, and opportunities.
3. Child health concerns in Virginia and misconceptions preventing trainees from participating in advocacy (facilitator: local advocacy leader):
  • Host a discussion on the importance of advocacy for residents and share personal experiences. Highlight three main child health issues facing your state, existing work being conducted, and the level of legislature input.
4. AAP child health priorities and important currently pending legislation (facilitator: local legislative representative or AAP state lobbyist):
  • Organize a didactic with an introduction of your local AAP chapter, legislative successes, and upcoming legislative priorities.
5. Child nutrition and how to meet your representative (facilitator: local advocacy leaders):
  • Didactic:
    ○ Child nutrition and Special Supplemental Nutrition Program for Women, Infants, and Children in your state.
    ○ Existing state Department of Health programs for nutrition.
    ○ How to have interviews with your legislature.
    ○ How do you locate your legislature?
  • Workshop:
    ○ Case-based practice on conducting mock interviews with your legislature (case on infant nutrition).
6. SIDS and the art of negotiation (facilitator: local advocacy leaders):
  • Didactic:
    ○ SIDS in your state.
    ○ Department of Health programs to counter SIDS.
    ○ Art of negotiation: What are ways to effect change?
  • Workshop:
    ○ Case-based practice on the art of negotiation with decision makers (case on SIDS).
7. Child mental health and op-ed writing (facilitator: local advocacy leaders):
  • Didactic:
    ○ Child mental health statistics in your state.
    ○ Tips on writing op-eds.
    ○ Introduction to the local AAP chapter.
    ○ Introduction to the federal and state affairs office and section on medical students, residents, fellows, and trainees.
  • Workshop:
    ○ Case-based practice on op-ed writing (audience-choice cases on mental health).

The project was approved by the Eastern Virginia Medical School Institutional Review Board.

#### Reading materials

The precurriculum reading material provided was optional. The AAP *Advocacy Guide*^11^ was introduced to residents. This guide is free and accessible online. Residents were advised to read chapters 1, 3, and 4 prior to Lecture 1, Lecture 2, and Workshop 1, respectively.

#### Lectures

Lecture 1 covered the basics of health advocacy and an introduction to curriculum goals and real-life cases to stimulate interest (Appendix A). This lecture could be given by any faculty or resident. In our curriculum, it was delivered by one of the residents organizing the curriculum.

Lecture 2 was an overview of the legislative and bill implementation process, reviewing current priorities for child health policy and past progress in child health policy (Appendix B). This lecture should be given by an individual familiar with the legislative process, such as the local or state representative of the area. We found these individuals to be very interested in community outreach on subjects relating to child health in their constituencies. Collaborating with the local AAP chapter is a great way to identify and invite such individuals. In our curriculum, this lecture was delivered by the lieutenant governor of Virginia because he was a physician on faculty at our institution.

Lecture 3 described the fundamentals of advocacy and major child health issues in the area, and discussed challenges and misconceptions preventing trainees from participating in advocacy (Appendix C). This lecture should be given by an individual familiar with advocating to representatives and legislative bodies on behalf of child health issues. Considering that such advocacy is one of the important functions of the AAP, the local AAP chapter would be a good resource through which to identify a speaker. Alternatively, faculty members who are familiar with advocating can also deliver this didactic. In our curriculum, this lecture was delivered by the former Virginia health commissioner.

Lecture 4 focused on local AAP child health priorities, an in-depth overview of the state legislative process, and current pending legislation for which the AAP was actively advocating (Appendix D). A lecturer who is familiar with current legislative priorities should be identified through the local AAP chapter. In our curriculum, this lecture was delivered by an AAP state lobbyist.

#### Interactive workshops

Workshops can be facilitated by in-house or invited faculty members who have some baseline knowledge of health advocacy. In our curriculum, in-house faculty members who had been involved in child health advocacy facilitated the workshops.

Workshop 1 taught the issue of pediatric nutrition along with the advocacy skill of communicating with state representatives. The interactive scenario was called “Meet Your State Senator to Advocate for the Child Nutrition Authorization Bill 2016.” An example of a PowerPoint that highlighted the health issue of interest and proceeded to teach the skill in theory is shared in Appendix E. The skill was then practically taught in group format. The group facilitator acted as the senator, and residents advocated for a nutrition bill that was being considered in the House and Senate using a skill checklist that was developed by curriculum organizers (Appendix F). Appendix F is a framework learners use in engaging with the workshop. The answers to “What are the member/staff priorities for the coming year?” and “What committees does the representative/senator sit on?” should be known by the session facilitator in order for the facilitator to accurately embody the state senator's position. In our experience, this information was found online on the state legislature's government (.gov) website or on GovTrack.us (www.govtrack.us), where active bills and legislators' positions on them were described.

Workshop 2 taught the art of negotiation in the context of the child health issue of SIDS (Appendix G). This involved a scenario where residents practiced how to negotiate with a facilitator who acted as a Medicaid representative.

Workshop 3 taught residents how to write an op-ed article on a child mental health issue. A PowerPoint teaching the theoretical points of op-ed writing is shared in Appendix H. The small-group discussion culminated with each small group generating an outline of an op-ed and sharing it with the whole group.

At the conclusion of the curriculum, residents, with the help of curriculum facilitators, created a state-specific advocacy action plan (see the Figure). Residents devised a stepwise approach to facilitate effective communication with decision makers regarding health policies that were under current consideration in the legislature. The stepwise plan involved identifying bills and delegates using reliable and easily accessible websites; identifying channel, mode, and timing of contact; and establishing a follow-up plan. Residents then utilized the state-specific action plan by advocating at Virginia General Assembly Day 2017. Individuals in other programs can use the figure as a practical guide should they wish to communicate with their representatives.

**Figure.**
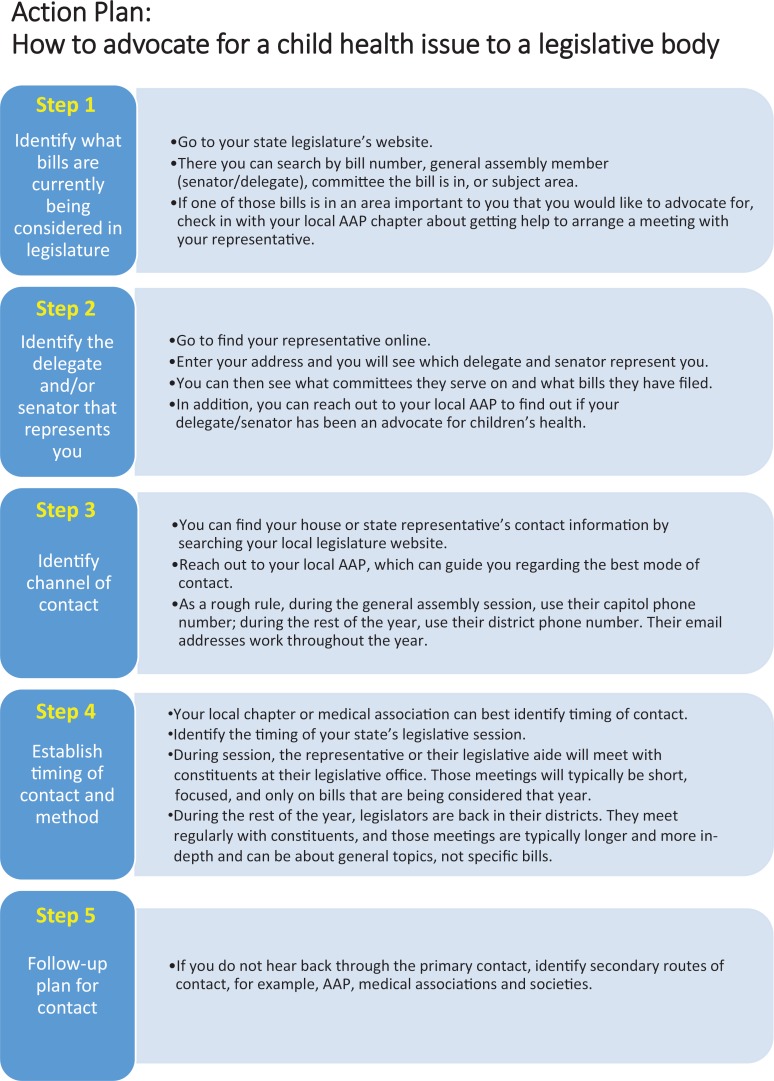
State-specific advocacy action plan. Abbreviation: AAP, American Academy of Pediatrics.

### Step 6: Evaluation and Feedback

Resident participation in the advocacy curriculum was voluntary. All who elected to participate received a precurriculum survey (Appendix I). Survey questions were developed using the AAP *Advocacy Guide.*^11^ The survey questions had not been previously validated.

We distributed the reading materials described earlier to participants. Residents who had reviewed the reading materials completed postcurriculum surveys that were identical to the precurriculum surveys. We numerically coded surveys to allow anonymous pre/post comparison between individuals.

Participants were asked for written feedback at each session.

Additionally, the Community Pediatrics Training Initiative leadership was involved in ongoing feedback via conference calls. A longitudinal collaboration was established and maintained throughout the curriculum implementation period in which the other three AAP grant recipients shared documents, educational modules, and other information about their advocacy projects in an online forum and on conference call discussions.

## Results

The curriculum generated several noteworthy outcomes, including (1) residents creating an advocacy action plan for community- and state-level advocacy, (2) residents collaborating with the local AAP to advocate at the local general assembly day, (3) strengthening of relationships with state- and community-level resources, (4) the curriculum being implemented as an annual offering for pediatrics residents, and (5) demonstrated improvement in resident knowledge and comfort level with health advocacy. Each of these is described in the following paragraphs.

After creation of the state-level legislative advocacy action plan, residents utilized skills acquired in real time and collaborated with the Virginia AAP to advocate at Virginia General Assembly Day 2017. Fifteen residents met their state representatives to advocate for legislation pertinent to opioid prescribing in the context of neonatal abstinence syndrome.

We had representative speakers from community organizations and the Virginia AAP who delivered a didactic session. This enabled the residents to network and learn more about resources available in the community not only for patients but also for potential future collaborations.

Sixty-four out of 70 residents (91%) completed the precurriculum survey and were invited to participate in the curriculum. Sixty-one out of 64 residents (95%) completed the postcurriculum survey. Thirty-three percent of curriculum participants were PGY 1, 31% were PGY 2, 30% were PGY 3, and 6% were PGY 4. In the precurriculum survey, most residents received news about pediatric health issues from AAP emails (87%) and the AAP website (66%). Few residents had been involved in community-, state-, or federal-level advocacy (18%, 2%, and 1%, respectively). Many residents were somewhat (31%) or moderately interested (33%) in learning how to effect change. However, almost half of pediatric residents were not comfortable communicating with (47%) or finding (41%) their representative.

Five questions on the postcurriculum survey had also been asked on the precurriculum survey. All five questions showed significant improvement in mean scores postcurriculum (Table 2): likelihood of speaking out for a child health issue, familiarity with the process of a bill becoming law, familiarity with online resources for advocacy training, familiarity with finding a legislative representative online, and comfort level in communicating with state and local representatives.

**Table 2.** Pre- and Postcurriculum Survey Results Showing Improvement in Knowledge of and Attitudes Toward Political Advocacy

Question^a^	Precurriculum	Postcurriculum	*p*
Likelihood of speaking out for a child health issue	2.30	3.48	<.001
Familiarity with the process of a bill becoming law	2.17	2.63	<.001
Familiarity with online resources for advocacy training	1.56	2.38	<.001
Familiarity with finding a legislative representative online	1.82	2.65	<.001
Comfort level in communicating with state and local representatives	1.77	2.33	.001

a Rated on a 5-point scale (1 = *not at all*, 2 = *slightly*, 3 = *moderately*, 4 = *very*, 5 = *extremely*).

## Discussion

Our experience shows successful implementation of a locally relevant advocacy curriculum now in its second year. The curriculum updated residents on community, state, and national child health issues, and it taught them the skills needed to advocate effectively. It was well received by residents and resulted in improvements in resident knowledge and attitudes regarding child health advocacy.

The goals, objectives, and educational strategies of this curriculum are reproducible and feasible. They can be utilized by other pediatric residency programs and tailored to state-specific health issues. Using a state-specific child health needs assessment can be informative in choosing health issues around which workshops can be modeled. This can enhance residents' awareness of local health issues and empower them to direct their future advocacy efforts toward pressing child health concerns. Most programs have the capability to form the types of collaborations utilized in our curriculum, such as with their local AAP chapter. The local chapter can facilitate communication and extend invitations to local representatives who can deliver didactics in pediatric facilities. These collaborations can also be helpful in providing appropriate tools that can be utilized to teach various advocacy skills. Most programs have mandatory educational conferences into which didactics and workshops could be incorporated. The didactics were highly attended in our curriculum, perhaps in part due to our high-profile speakers. This may not be reproducible if institutions do not have proximity to such speakers; however, one of our best-attended invited speaker lectures was given by an AAP lobbyist who was not known to the residents. Additionally, a stepwise advocacy action plan can easily be created for different states. This can further be modified by choosing a specific bill of interest and generating steps to best address an individual's or group's position on certain legislation. In addition, most pediatric residency programs have the ability to participate in a local advocacy day or general assembly day where residents can practice the skills acquired from such a curriculum. As with exposure to quality improvement programs, advocacy exposure should be longitudinal throughout postgraduate medical education, and an effort should be made to maximize time allocation where possible.^12^

Studies have shown that exposure to advocacy teaching and training during residency leads to a higher rate of resident participation in community involvement and advocacy during and after residency.^13,14^ We found that blocks of protected time were useful for effectively teaching residents practical advocacy skills. To increase participation in curricular activities, we suggest that interested residents be provided with protected time in which they can focus on their advocacy interests.

Multiple challenges were encountered during this project. Specific roles were not assigned to members in the steering committee. In retrospect, assigning roles would have facilitated smoother preparation, work division, and delivery of the curriculum. Core facilitator availability for each workshop was difficult to arrange due to conflicting schedules. Having a variety of faculty facilitators for each workshop may be feasible for future workshops. Resident attendance due to clinical duties was another barrier. We found that when on inpatient wards, residents were unable to attend the workshops or would have to leave midway. Resident participation in the survey was challenging because the survey was in paper form instead of electronic.

We did not formally assess real-world application of skills taught or long-term change in practices with regard to advocacy. A limitation of our study is the lack of long-term data on residents going on to advocate for bills or participate in legislative advocacy days in the future. A future study is planned to survey residents who took part in the curriculum to assess long-term influence of the curriculum on their involvement in political advocacy in their future careers.

We have shown that a reproducible, feasible, and locally relevant longitudinal advocacy curriculum can be successfully developed and implemented to improve child health advocacy knowledge and attitudes in pediatric residents. Since the implementation of this curriculum, a core advocacy committee, including residents and faculty, has been formed in our program that will continue educational activities annually. The committee further aims to assess the influence of this curriculum on resident behavior and real-world application of advocacy skills. The AAP continues to be a key partner in yearly curriculum evolution. The ACGME alludes to the importance of advocacy training during pediatric residency, and we have suggested the development of specific recommendations with regard to the content and layout of such curricula. Other pediatric residency programs can utilize our curricular framework and resources to implement a longitudinal advocacy curriculum.

## Appendices

A. Lecture 1.pptx
B. Lecture 2.pptx
C. Lecture 3.ppt
D. Lecture 4.pptx
E. Workshop 1.pptx
F. Workshop 1 Skill Checklist.pdf
G. Workshop 2.pptx
H. Workshop 3.pptx
I. Curriculum Survey.docx All appendices are peer reviewed as integral parts of the Original Publication.
