# Supplementary material for: Advancing Advocacy: Implementation of a Child Health Advocacy Curriculum in a Pediatrics Residency Program
Source: MedEdPORTAL. 2020 Feb 14;16:10882. doi: 10.15766/mep_2374-8265.10882 (PMC7062538; doi:10.15766/mep_2374-8265.10882)
Supplement: Supplementary file 1 — A. Lecture 1.pptx B. Lecture 2.pptx C. Lecture 3.ppt D. Lecture 4.pptx E. Workshop 1.pptx F. Workshop 1 Skill Checklist.pdf G. Workshop 2.pptx H. Workshop 3.pptx I. Curriculum Survey.docx [file mep-16-10882-s001.zip › A. Lecture 1.pptx]

## Slide 1
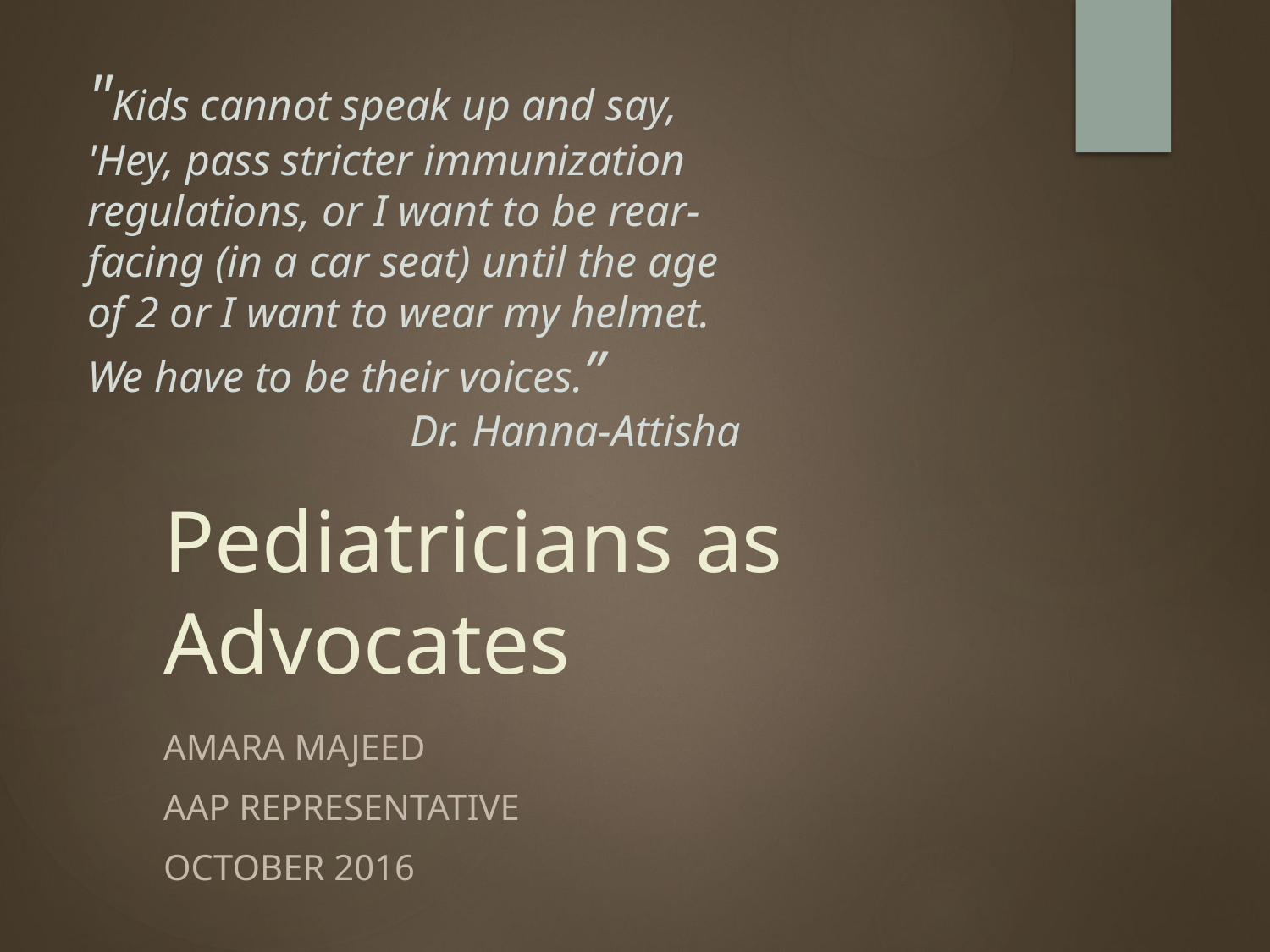

"Kids cannot speak up and say, 'Hey, pass stricter immunization regulations, or I want to be rear-facing (in a car seat) until the age of 2 or I want to wear my helmet. We have to be their voices.”
 Dr. Hanna-Attisha
# Pediatricians as Advocates
Amara majeed
Aap representative
October 2016

## Slide 2
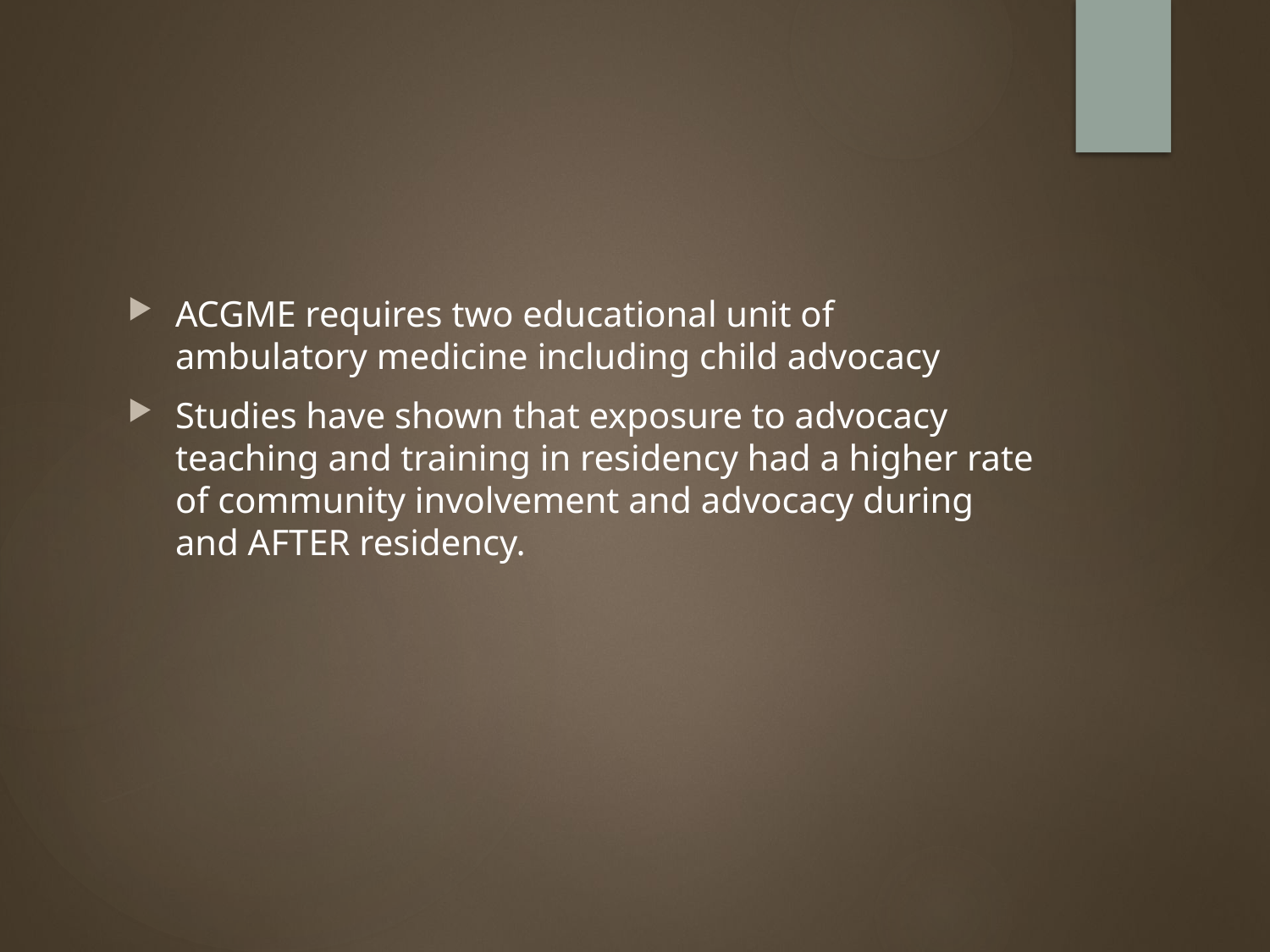

#
ACGME requires two educational unit of ambulatory medicine including child advocacy
Studies have shown that exposure to advocacy teaching and training in residency had a higher rate of community involvement and advocacy during and AFTER residency.

## Slide 3
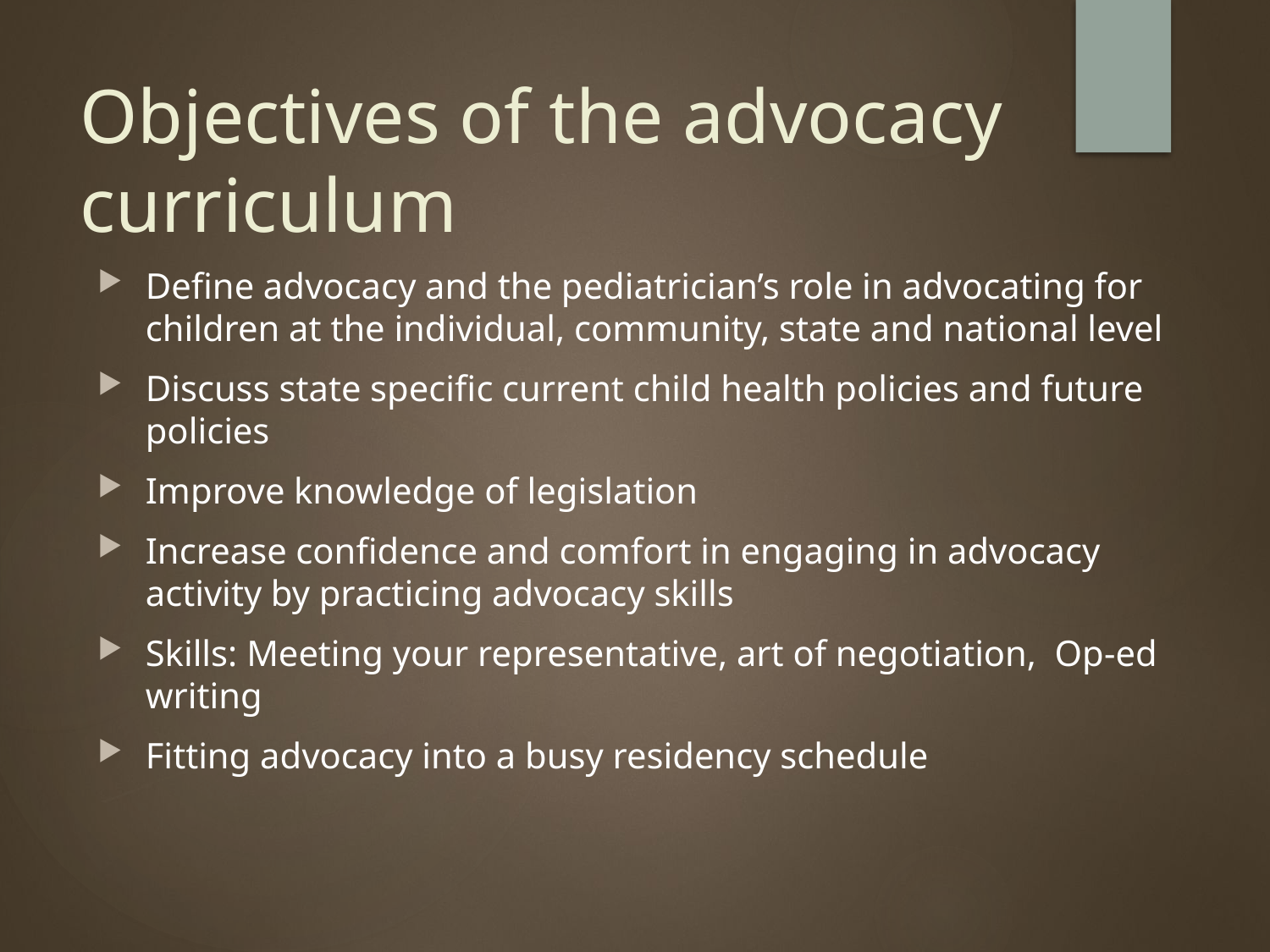

# Objectives of the advocacy curriculum
Define advocacy and the pediatrician’s role in advocating for children at the individual, community, state and national level
Discuss state specific current child health policies and future policies
Improve knowledge of legislation
Increase confidence and comfort in engaging in advocacy activity by practicing advocacy skills
Skills: Meeting your representative, art of negotiation, Op-ed writing
Fitting advocacy into a busy residency schedule

## Slide 4
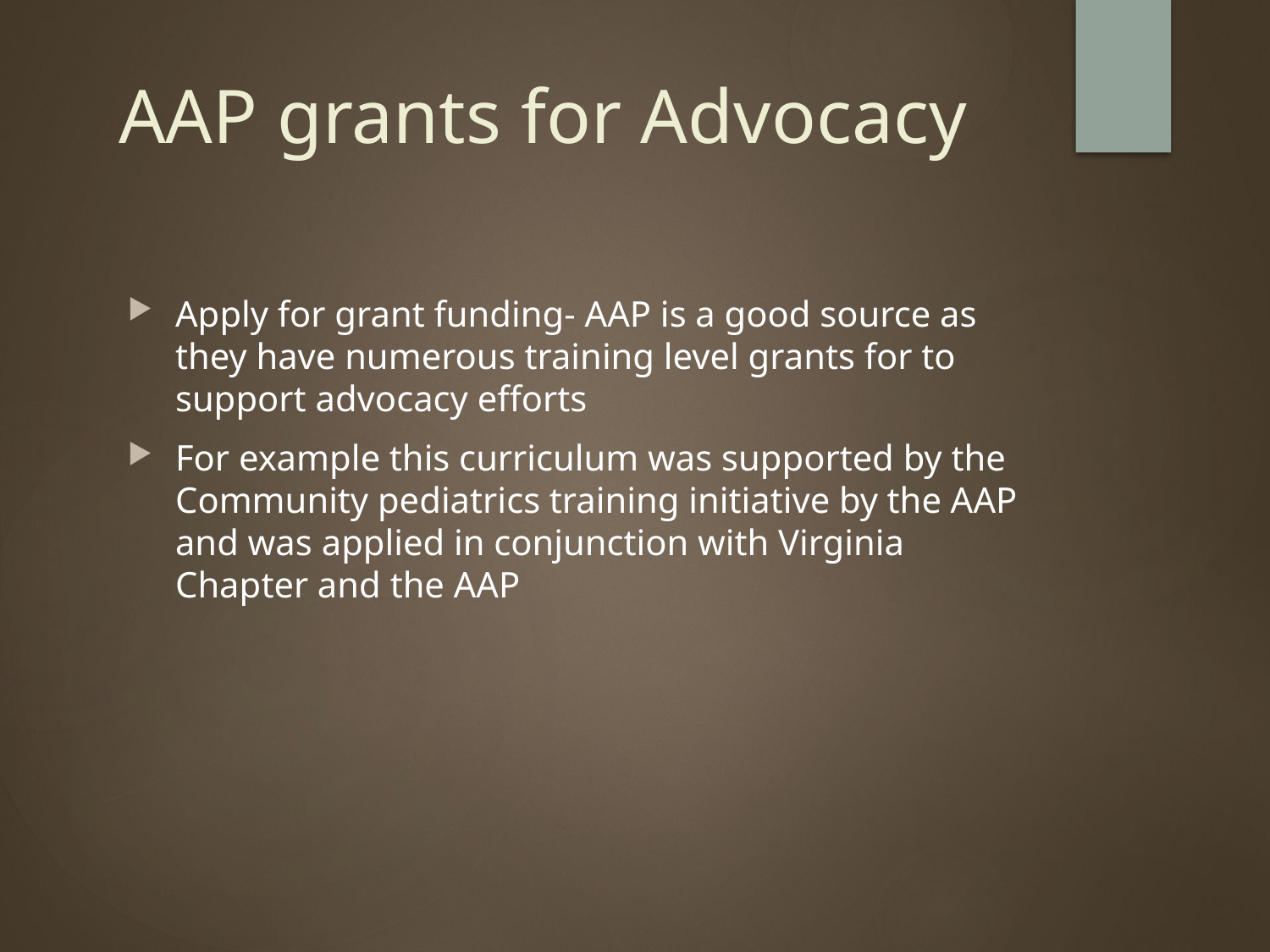

# AAP grants for Advocacy
Apply for grant funding- AAP is a good source as they have numerous training level grants for to support advocacy efforts
For example this curriculum was supported by the Community pediatrics training initiative by the AAP and was applied in conjunction with Virginia Chapter and the AAP

## Slide 5
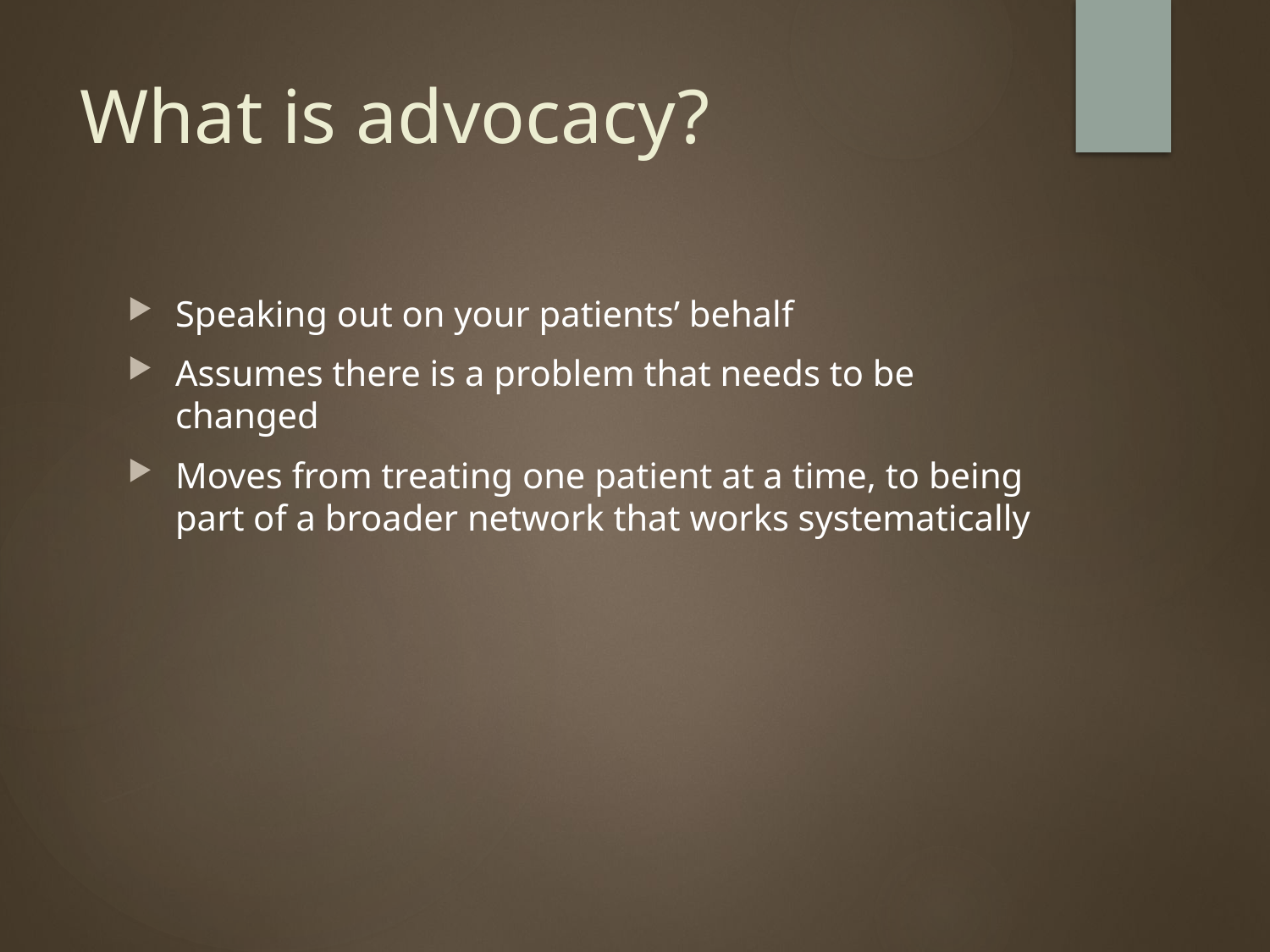

# What is advocacy?
Speaking out on your patients’ behalf
Assumes there is a problem that needs to be changed
Moves from treating one patient at a time, to being part of a broader network that works systematically

## Slide 6
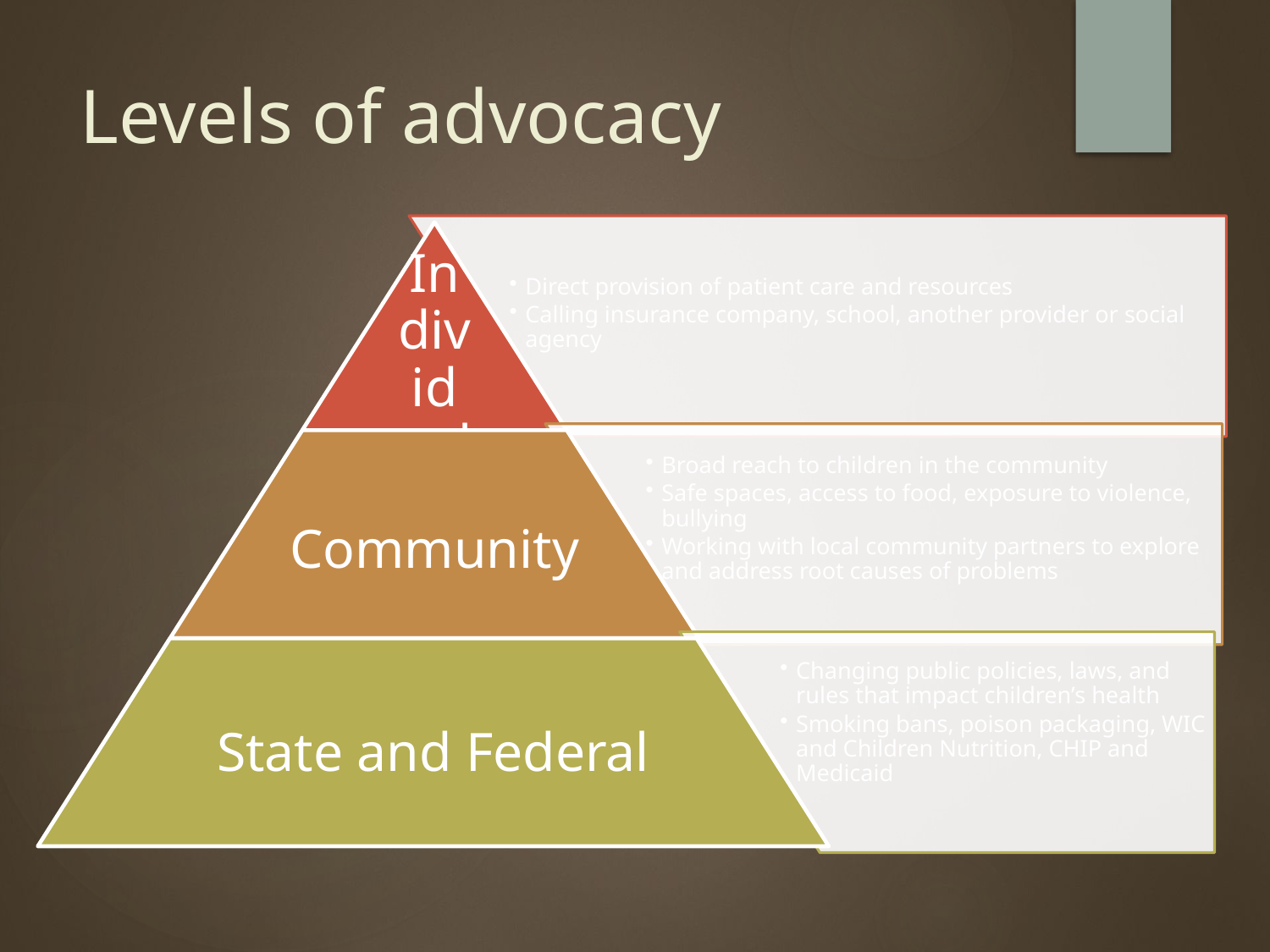

# Levels of advocacy

## Slide 7
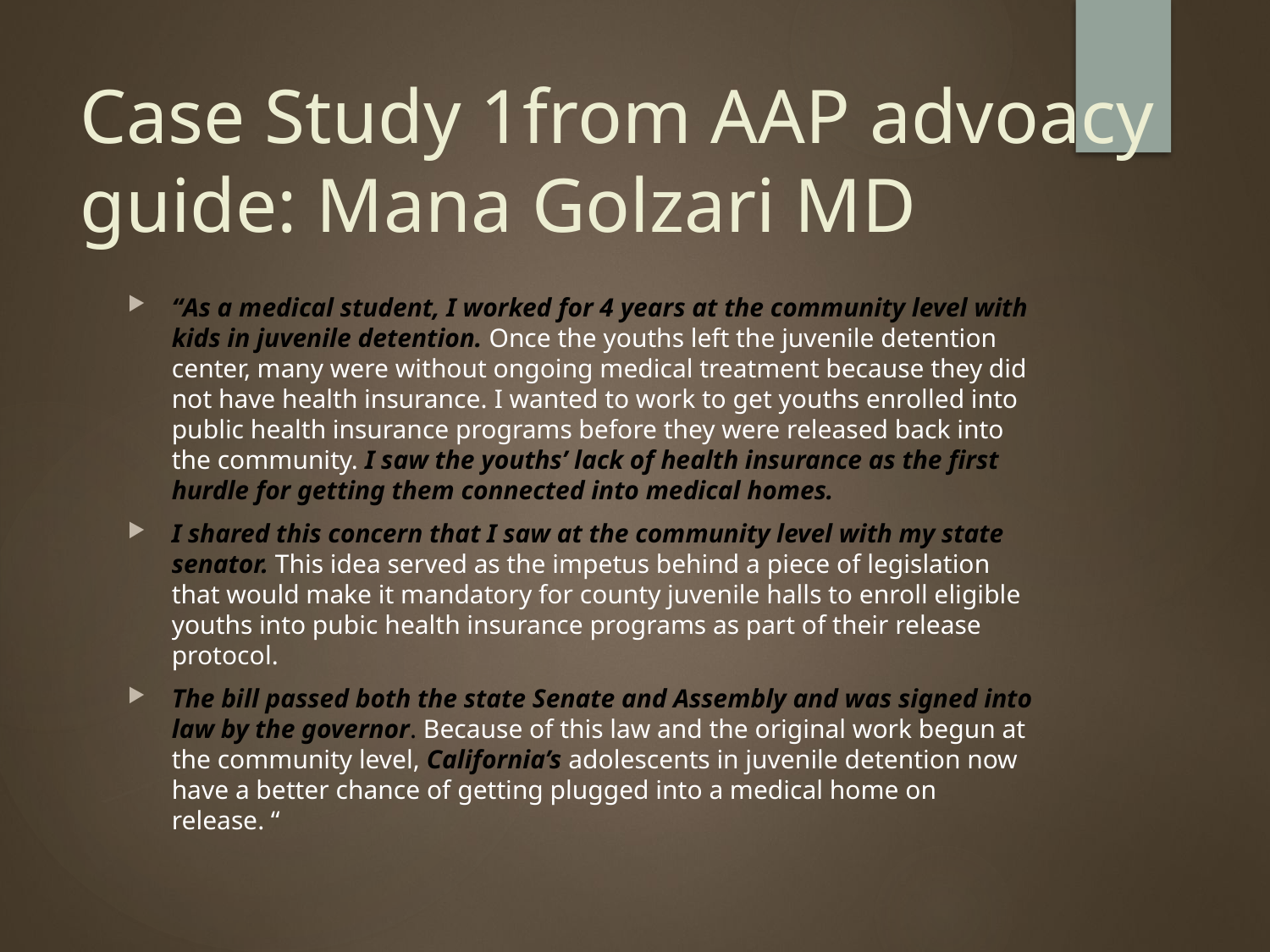

# Case Study 1from AAP advoacy guide: Mana Golzari MD
“As a medical student, I worked for 4 years at the community level with kids in juvenile detention. Once the youths left the juvenile detention center, many were without ongoing medical treatment because they did not have health insurance. I wanted to work to get youths enrolled into public health insurance programs before they were released back into the community. I saw the youths’ lack of health insurance as the first hurdle for getting them connected into medical homes.
I shared this concern that I saw at the community level with my state senator. This idea served as the impetus behind a piece of legislation that would make it mandatory for county juvenile halls to enroll eligible youths into pubic health insurance programs as part of their release protocol.
The bill passed both the state Senate and Assembly and was signed into law by the governor. Because of this law and the original work begun at the community level, California’s adolescents in juvenile detention now have a better chance of getting plugged into a medical home on release. “

## Slide 8
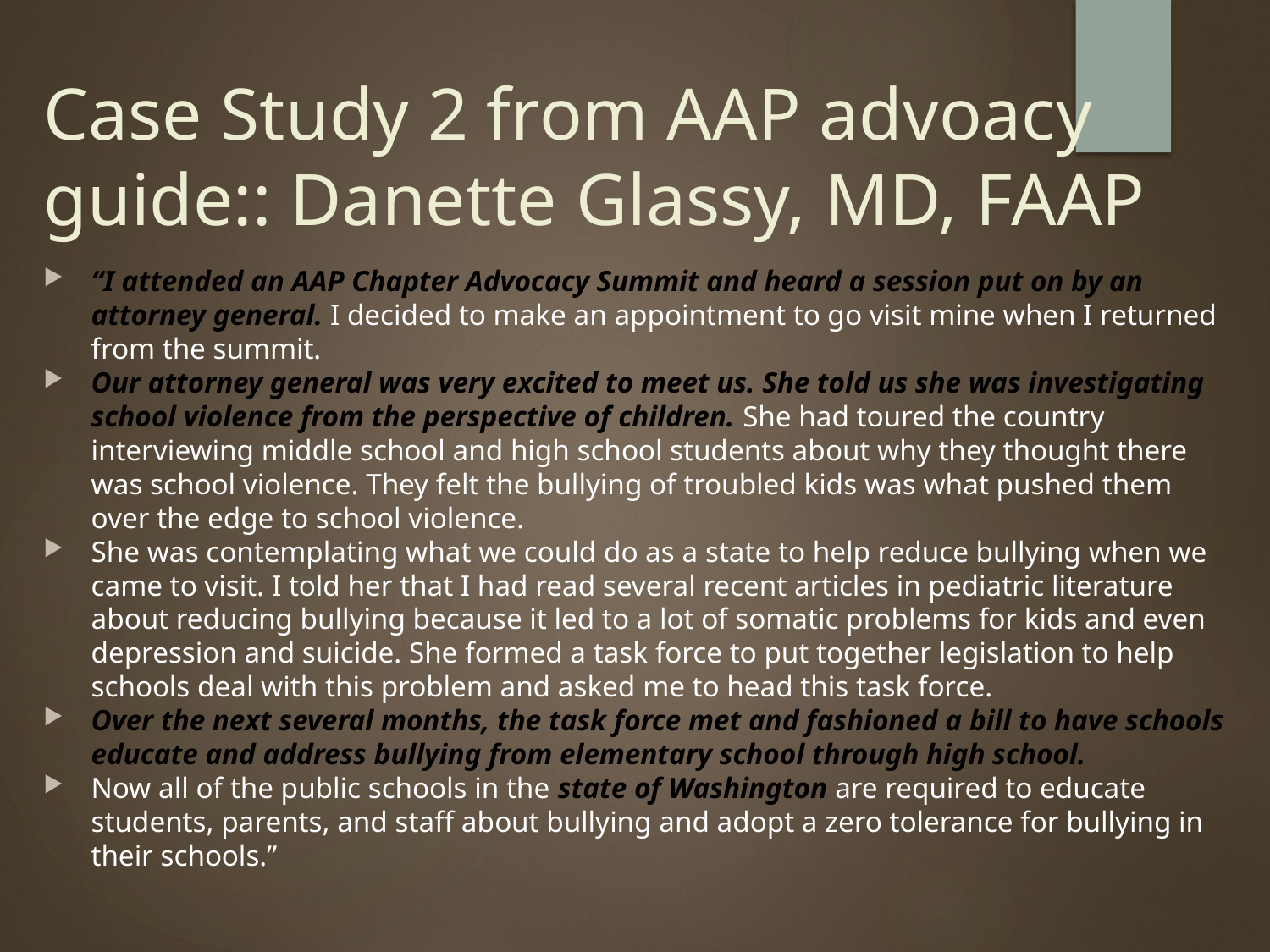

# Case Study 2 from AAP advoacy guide:: Danette Glassy, MD, FAAP
“I attended an AAP Chapter Advocacy Summit and heard a session put on by an attorney general. I decided to make an appointment to go visit mine when I returned from the summit.
Our attorney general was very excited to meet us. She told us she was investigating school violence from the perspective of children. She had toured the country interviewing middle school and high school students about why they thought there was school violence. They felt the bullying of troubled kids was what pushed them over the edge to school violence.
She was contemplating what we could do as a state to help reduce bullying when we came to visit. I told her that I had read several recent articles in pediatric literature about reducing bullying because it led to a lot of somatic problems for kids and even depression and suicide. She formed a task force to put together legislation to help schools deal with this problem and asked me to head this task force.
Over the next several months, the task force met and fashioned a bill to have schools educate and address bullying from elementary school through high school.
Now all of the public schools in the state of Washington are required to educate students, parents, and staff about bullying and adopt a zero tolerance for bullying in their schools.”

## Slide 9
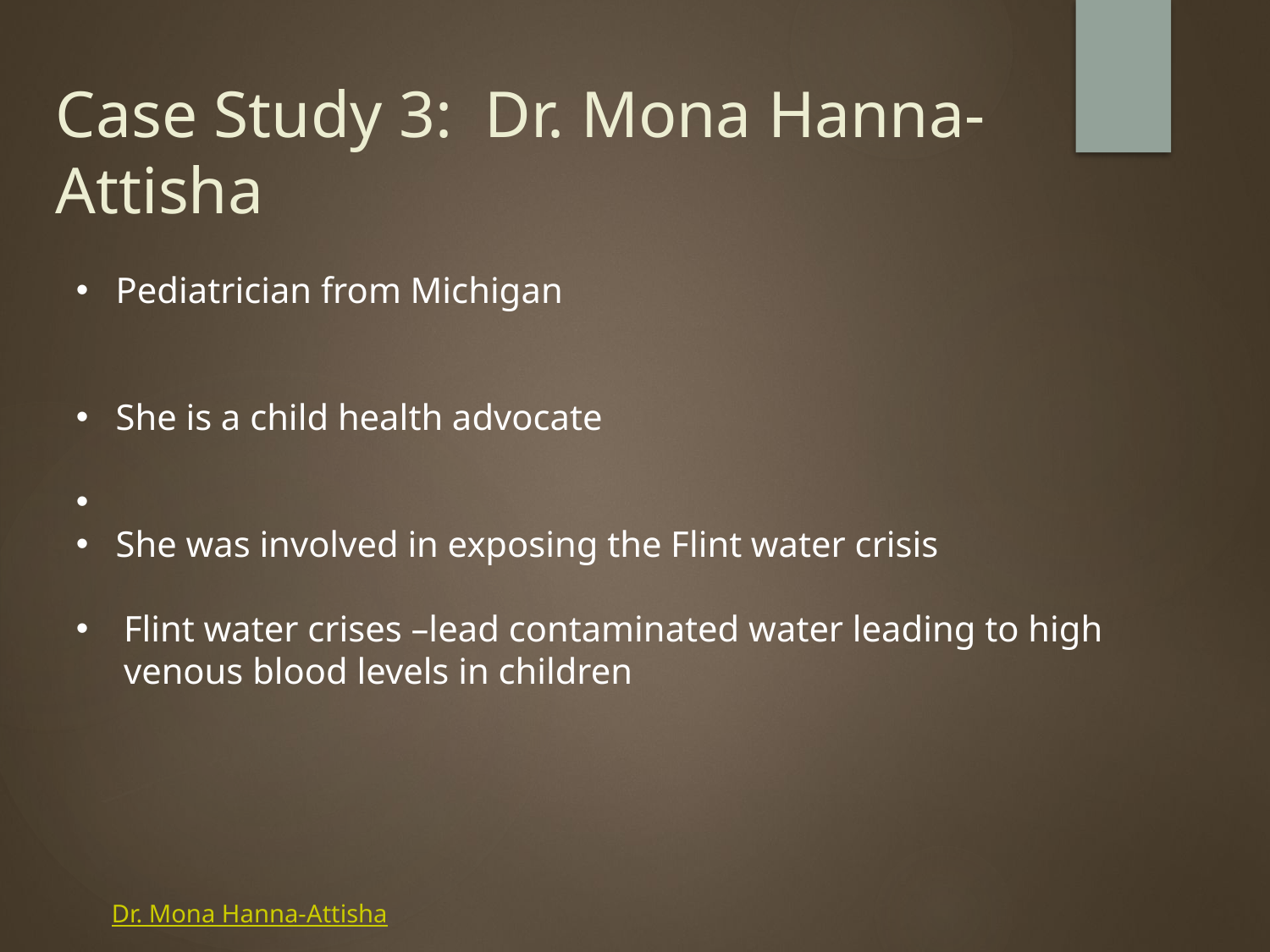

# Case Study 3: Dr. Mona Hanna-Attisha
Pediatrician from Michigan
She is a child health advocate
She was involved in exposing the Flint water crisis
Flint water crises –lead contaminated water leading to high venous blood levels in children
Dr. Mona Hanna-Attisha

## Slide 10
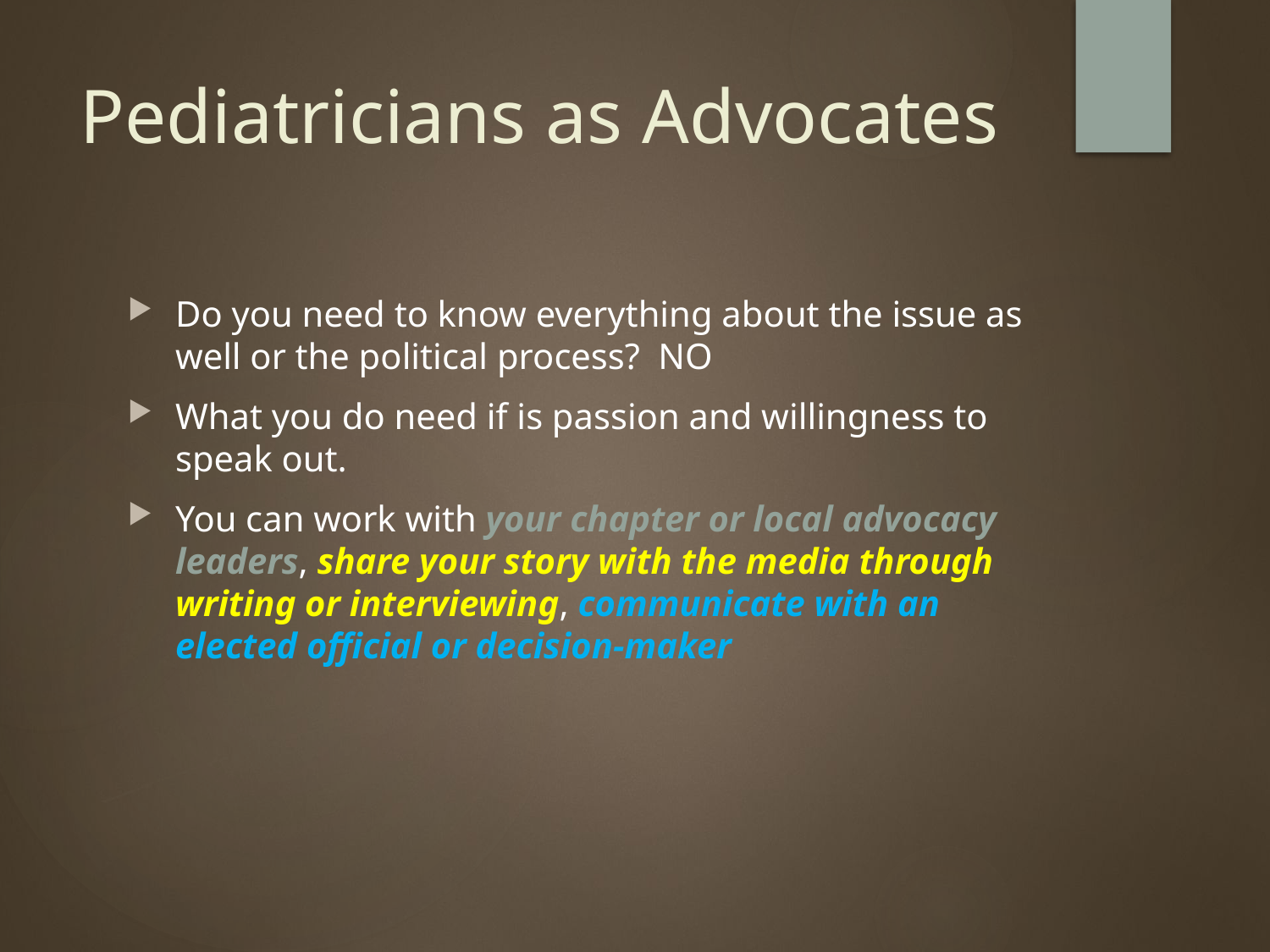

# Pediatricians as Advocates
Do you need to know everything about the issue as well or the political process? NO
What you do need if is passion and willingness to speak out.
You can work with your chapter or local advocacy leaders, share your story with the media through writing or interviewing, communicate with an elected official or decision-maker

## Slide 11
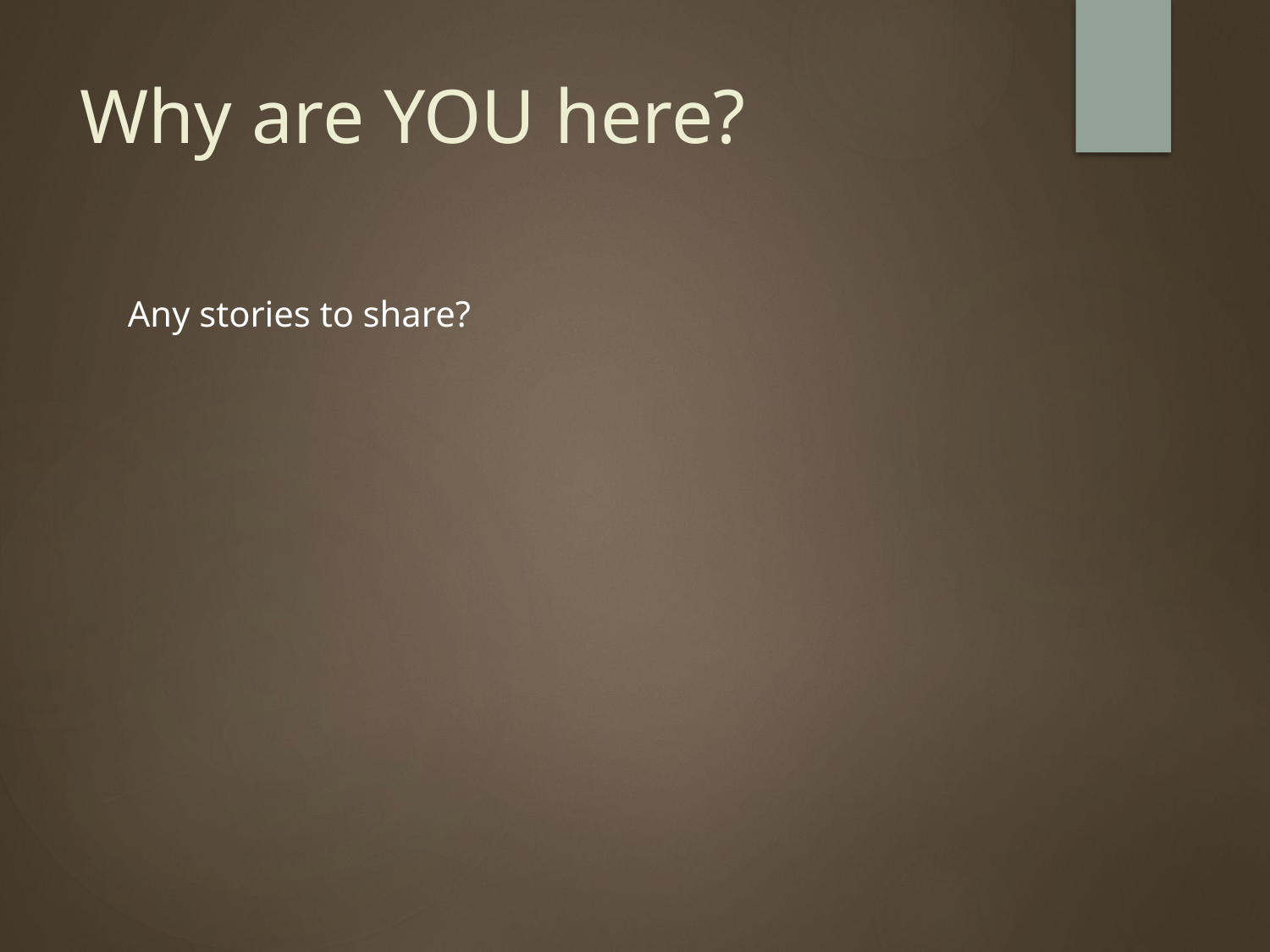

# Why are YOU here?
Any stories to share?

## Slide 12
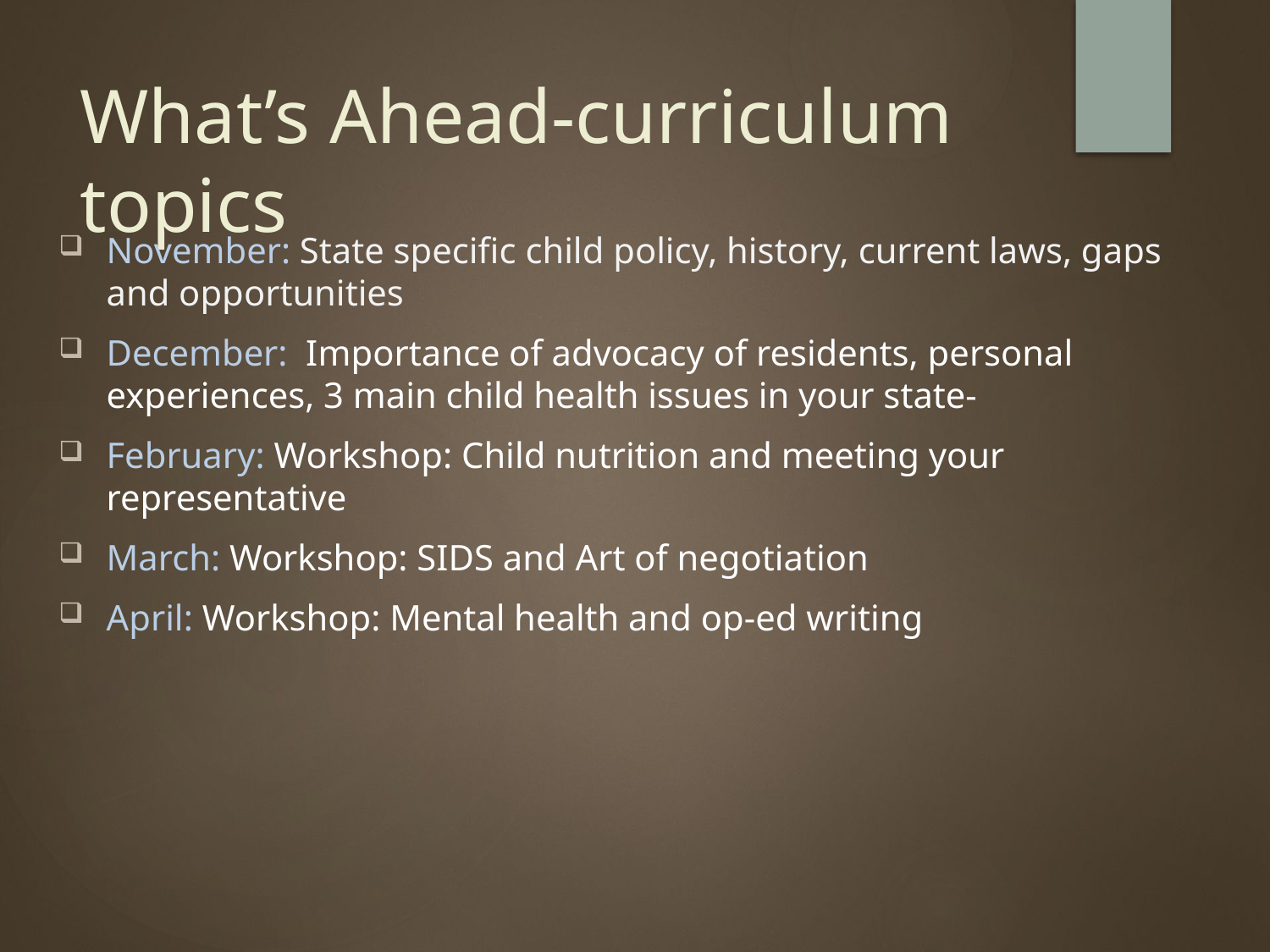

# What’s Ahead-curriculum topics
November: State specific child policy, history, current laws, gaps and opportunities
December: Importance of advocacy of residents, personal experiences, 3 main child health issues in your state-
February: Workshop: Child nutrition and meeting your representative
March: Workshop: SIDS and Art of negotiation
April: Workshop: Mental health and op-ed writing

## Slide 13
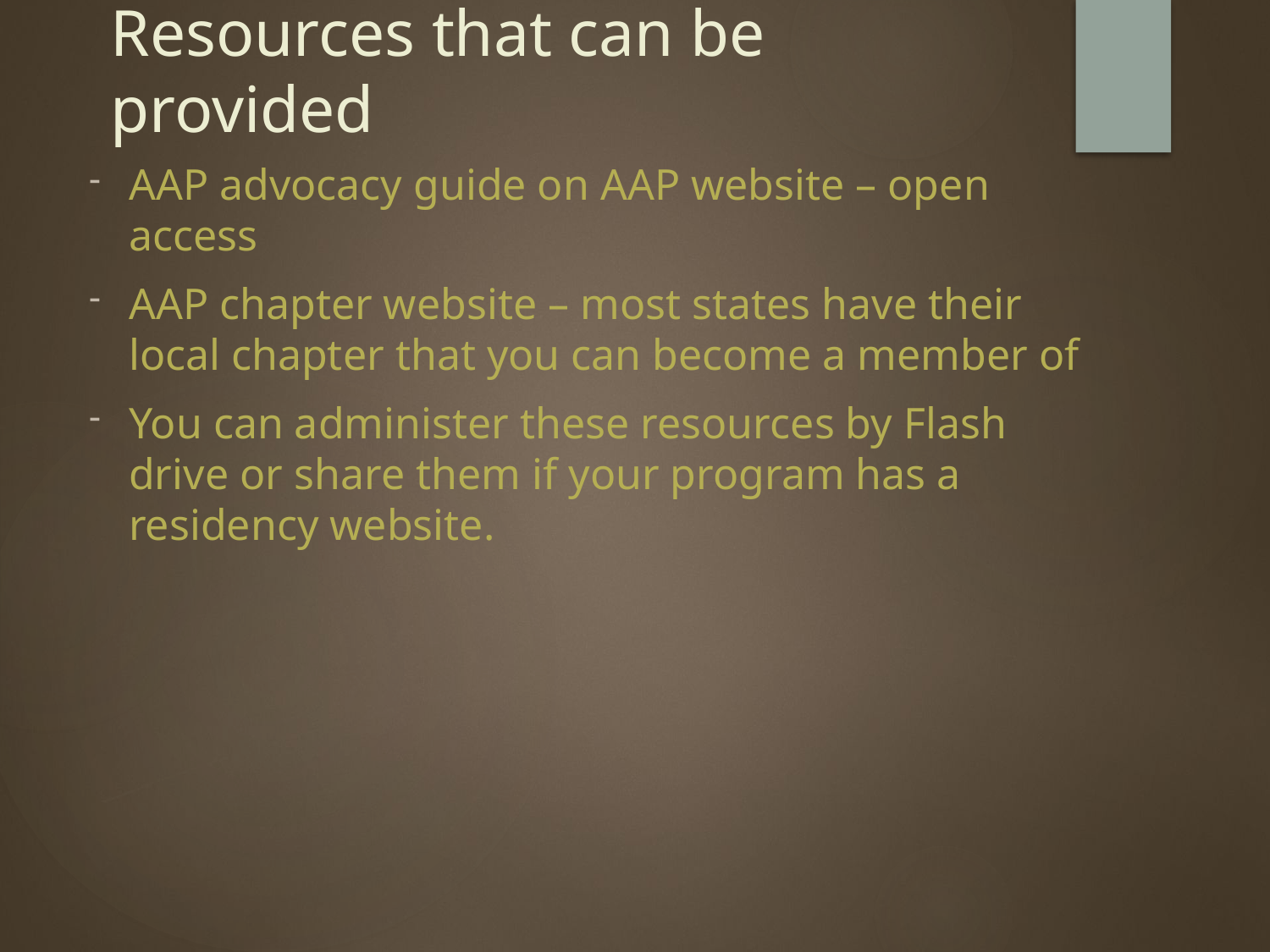

# Resources that can be provided
AAP advocacy guide on AAP website – open access
AAP chapter website – most states have their local chapter that you can become a member of
You can administer these resources by Flash drive or share them if your program has a residency website.

## Slide 14
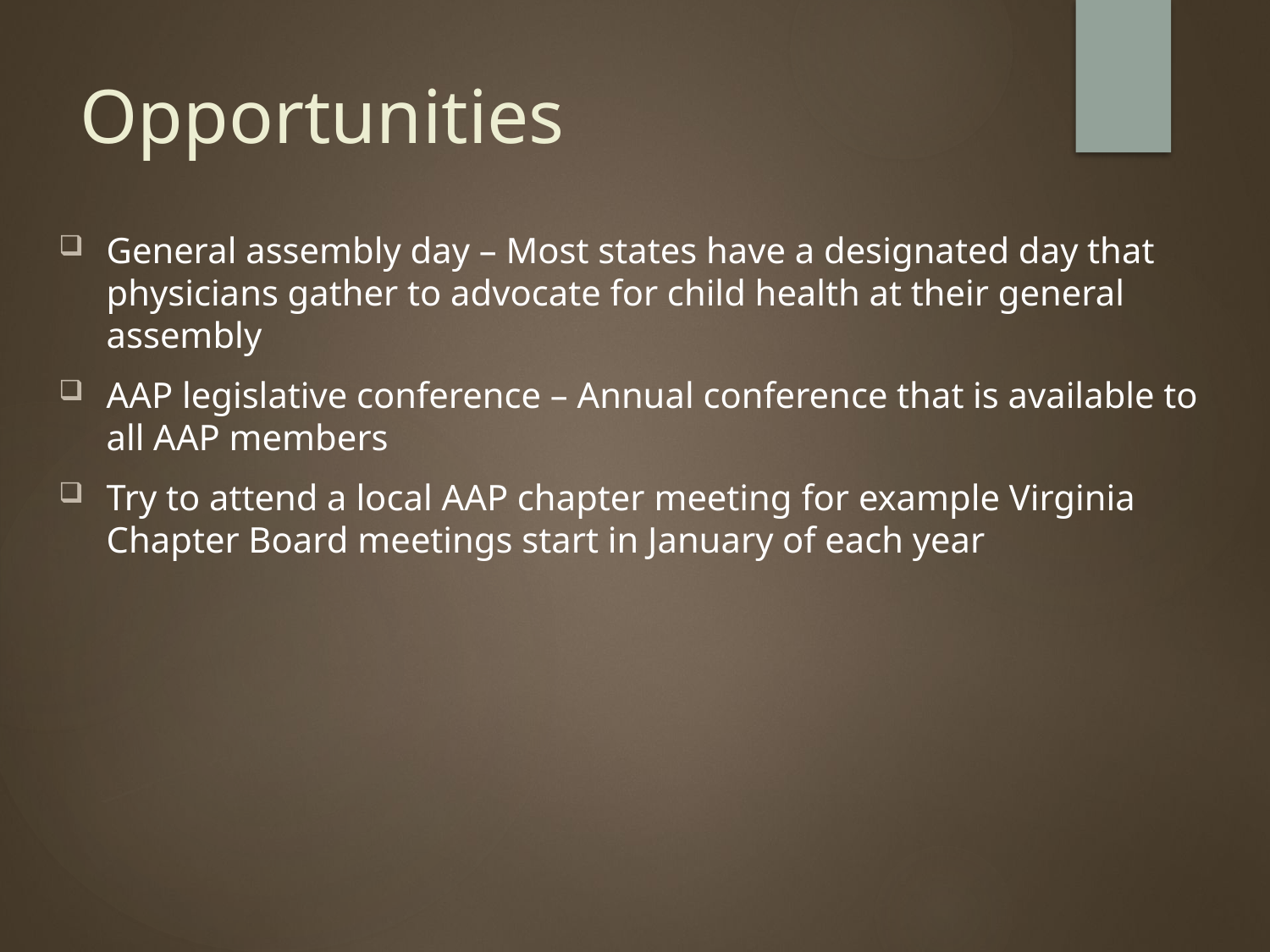

# Opportunities
General assembly day – Most states have a designated day that physicians gather to advocate for child health at their general assembly
AAP legislative conference – Annual conference that is available to all AAP members
Try to attend a local AAP chapter meeting for example Virginia Chapter Board meetings start in January of each year
