# Supplementary material for: Advancing Advocacy: Implementation of a Child Health Advocacy Curriculum in a Pediatrics Residency Program
Source: MedEdPORTAL. 2020 Feb 14;16:10882. doi: 10.15766/mep_2374-8265.10882 (PMC7062538; doi:10.15766/mep_2374-8265.10882)

## Slide 1
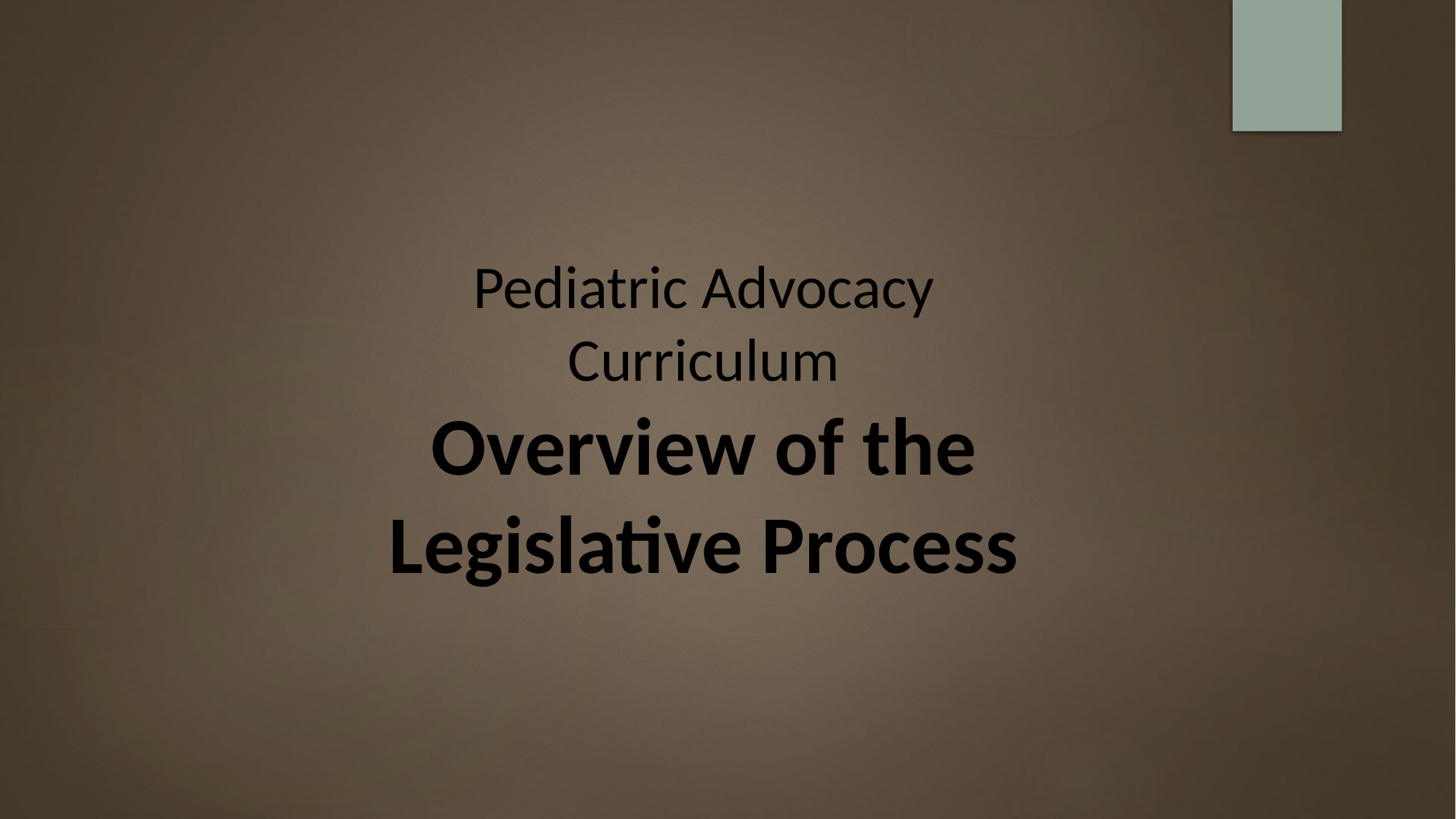

‘
Pediatric Advocacy
Curriculum
Overview of the
Legislative Process

## Slide 2
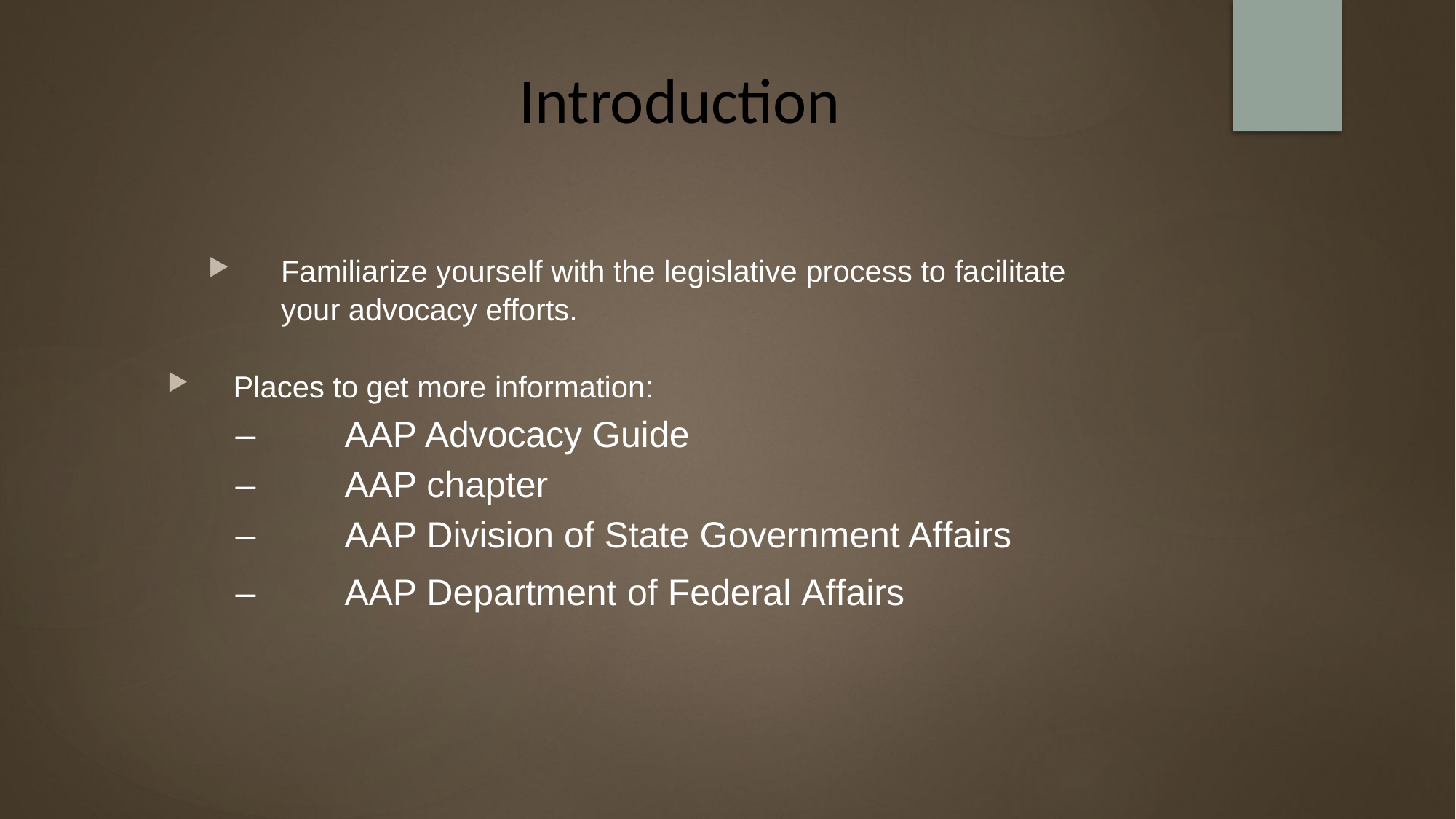

# Introduction
Familiarize yourself with the legislative process to facilitate your advocacy efforts.
 Places to get more information:
–	AAP Advocacy Guide
–	AAP chapter
–	AAP Division of State Government Affairs
–	AAP Department of Federal Affairs

## Slide 3
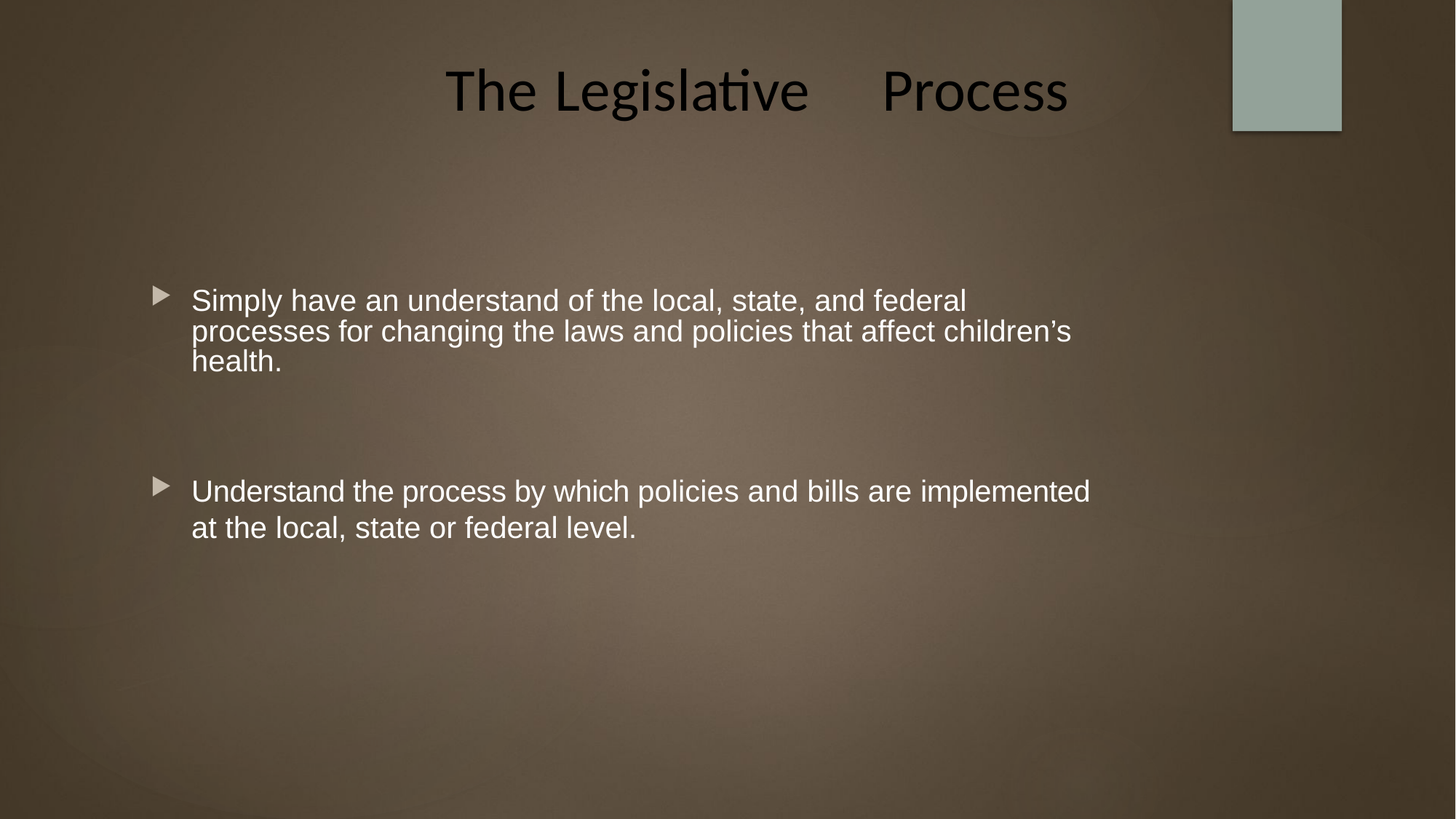

The	Legislative	Process
Simply have an understand of the local, state, and federal processes for changing the laws and policies that affect children’s health.
Understand the process by which policies and bills are implemented at the local, state or federal level.

## Slide 4
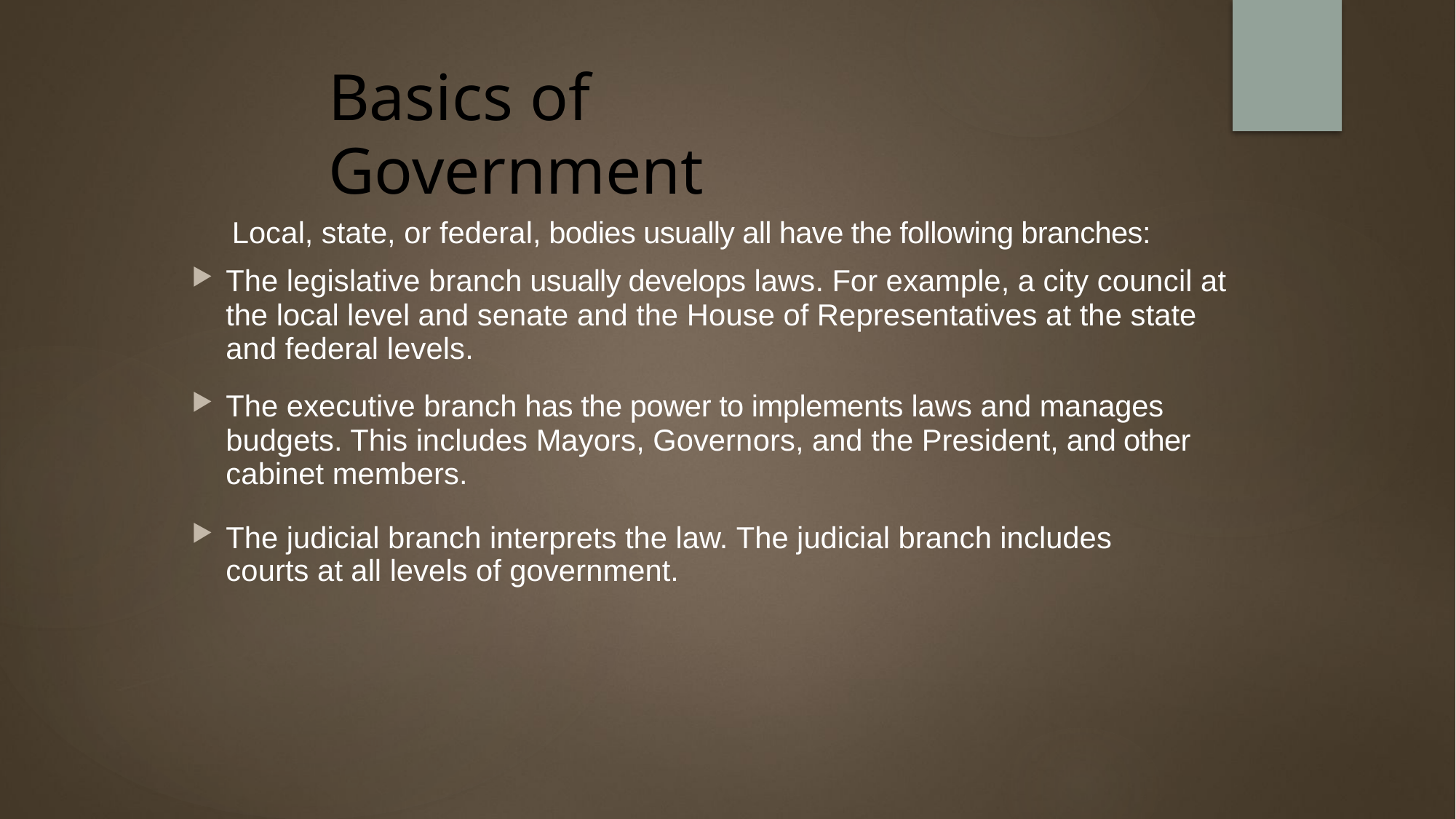

# Basics of	Government
Local, state, or federal, bodies usually all have the following branches:
The legislative branch usually develops laws. For example, a city council at the local level and senate and the House of Representatives at the state and federal levels.
The executive branch has the power to implements laws and manages budgets. This includes Mayors, Governors, and the President, and other cabinet members.
The judicial branch interprets the law. The judicial branch includes courts at all levels of government.

## Slide 5
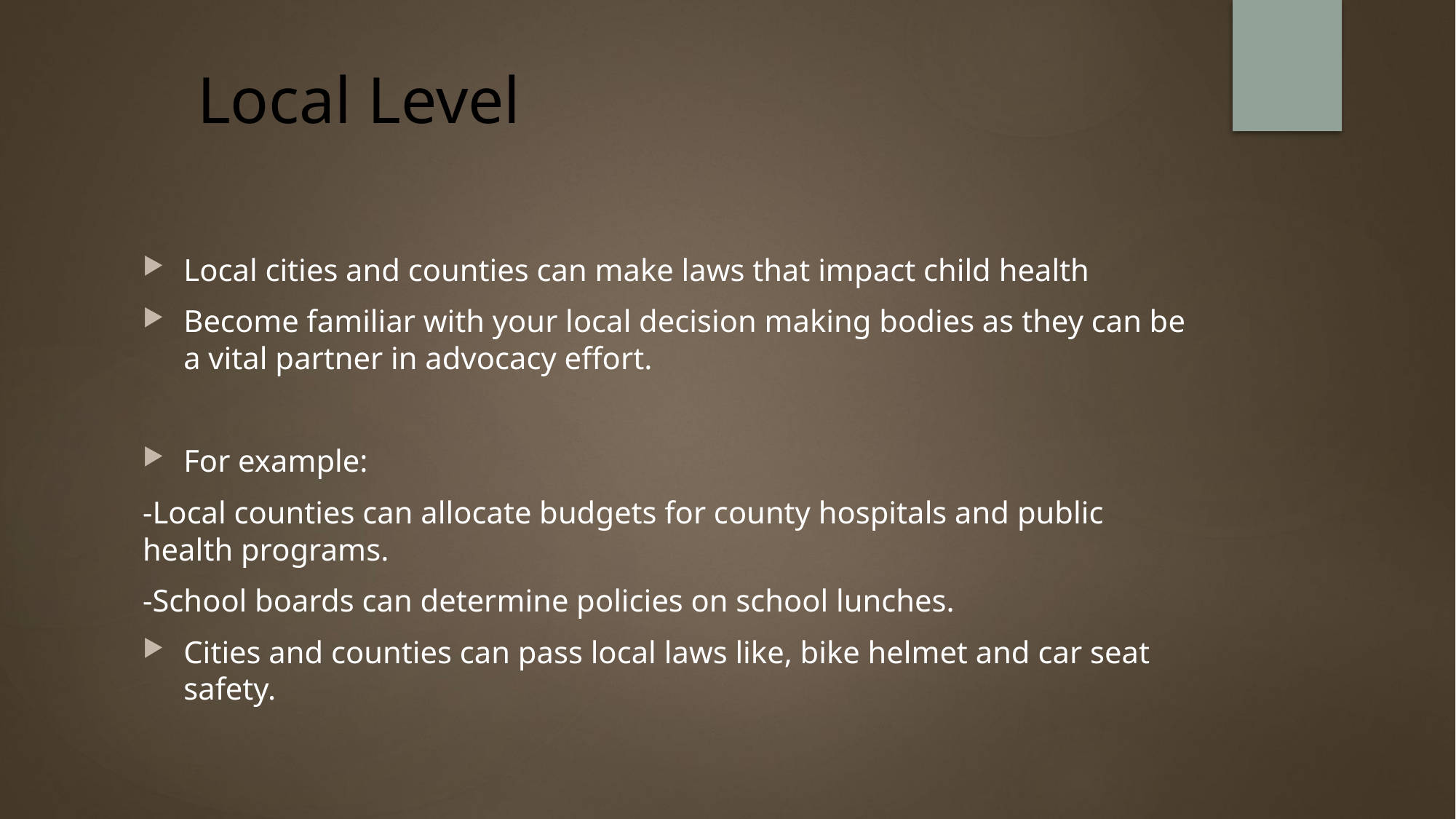

# Local Level
Local cities and counties can make laws that impact child health
Become familiar with your local decision making bodies as they can be a vital partner in advocacy effort.
For example:
-Local counties can allocate budgets for county hospitals and public health programs.
-School boards can determine policies on school lunches.
Cities and counties can pass local laws like, bike helmet and car seat safety.

## Slide 6
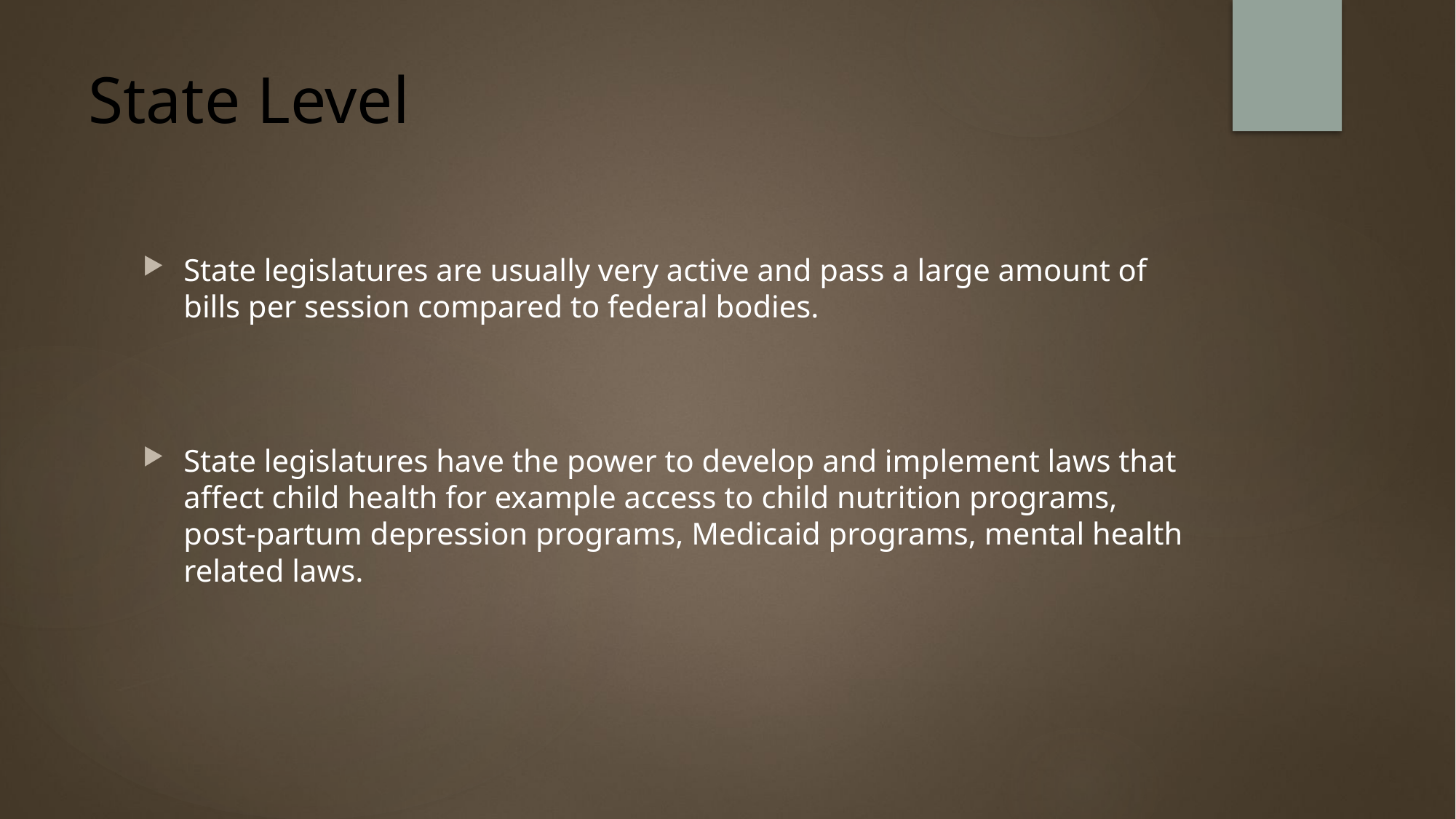

# State Level
State legislatures are usually very active and pass a large amount of bills per session compared to federal bodies.
State legislatures have the power to develop and implement laws that affect child health for example access to child nutrition programs, post-partum depression programs, Medicaid programs, mental health related laws.

## Slide 7
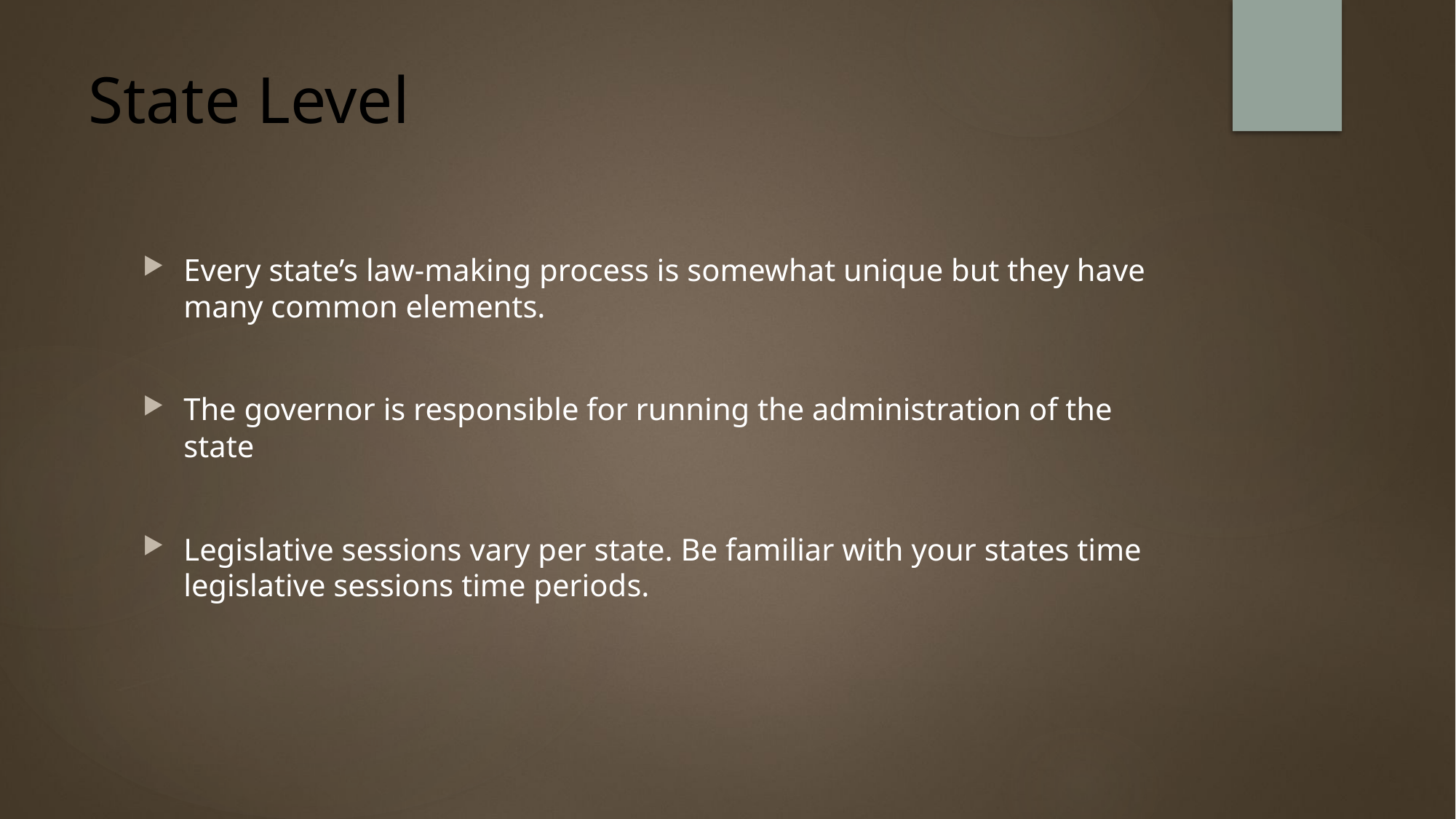

# State Level
Every state’s law-making process is somewhat unique but they have many common elements.
The governor is responsible for running the administration of the state
Legislative sessions vary per state. Be familiar with your states time legislative sessions time periods.

## Slide 8
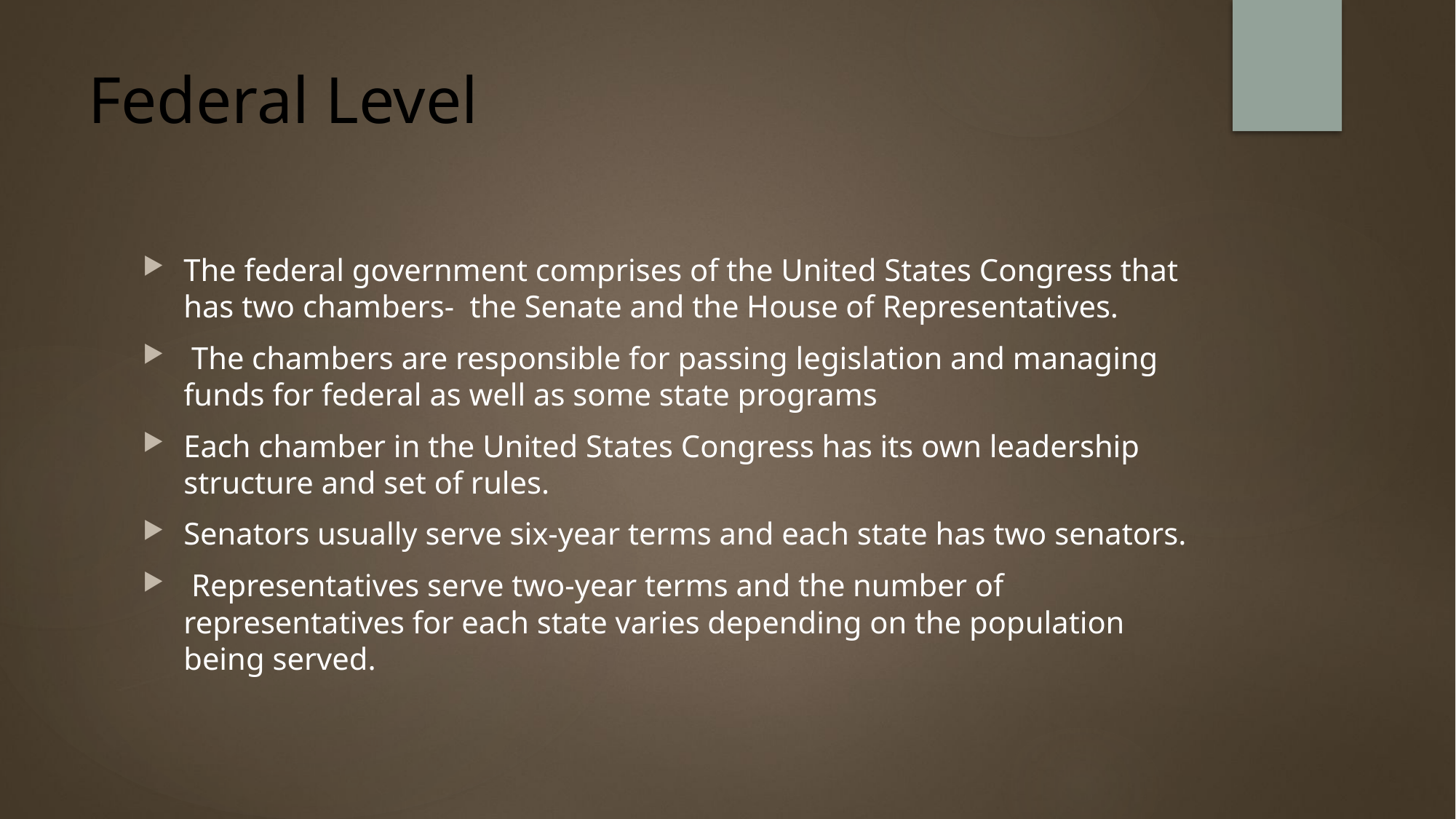

# Federal Level
The federal government comprises of the United States Congress that has two chambers- the Senate and the House of Representatives.
 The chambers are responsible for passing legislation and managing funds for federal as well as some state programs
Each chamber in the United States Congress has its own leadership structure and set of rules.
Senators usually serve six-year terms and each state has two senators.
 Representatives serve two-year terms and the number of representatives for each state varies depending on the population being served.

## Slide 9
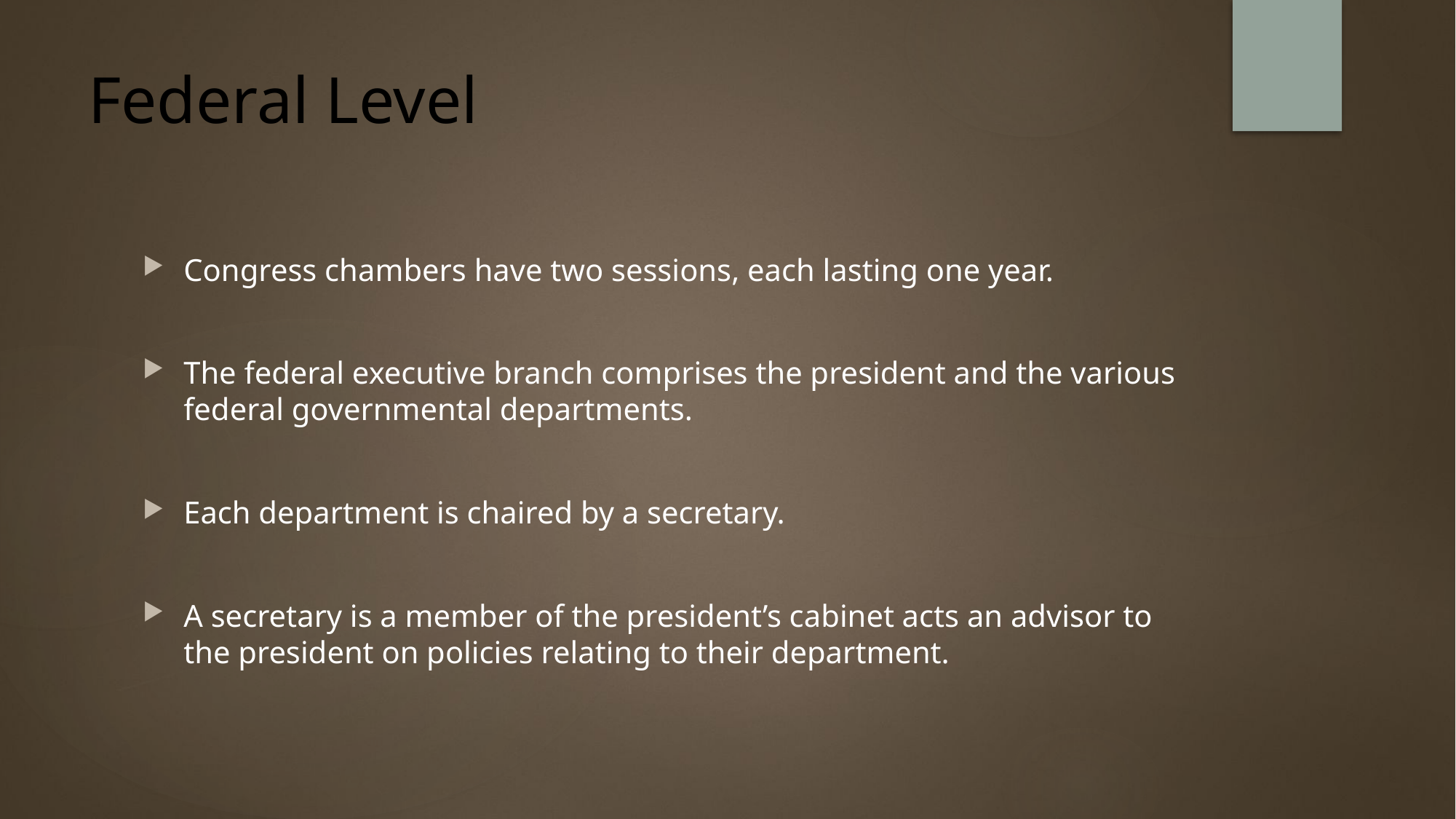

# Federal Level
Congress chambers have two sessions, each lasting one year.
The federal executive branch comprises the president and the various federal governmental departments.
Each department is chaired by a secretary.
A secretary is a member of the president’s cabinet acts an advisor to the president on policies relating to their department.

## Slide 10
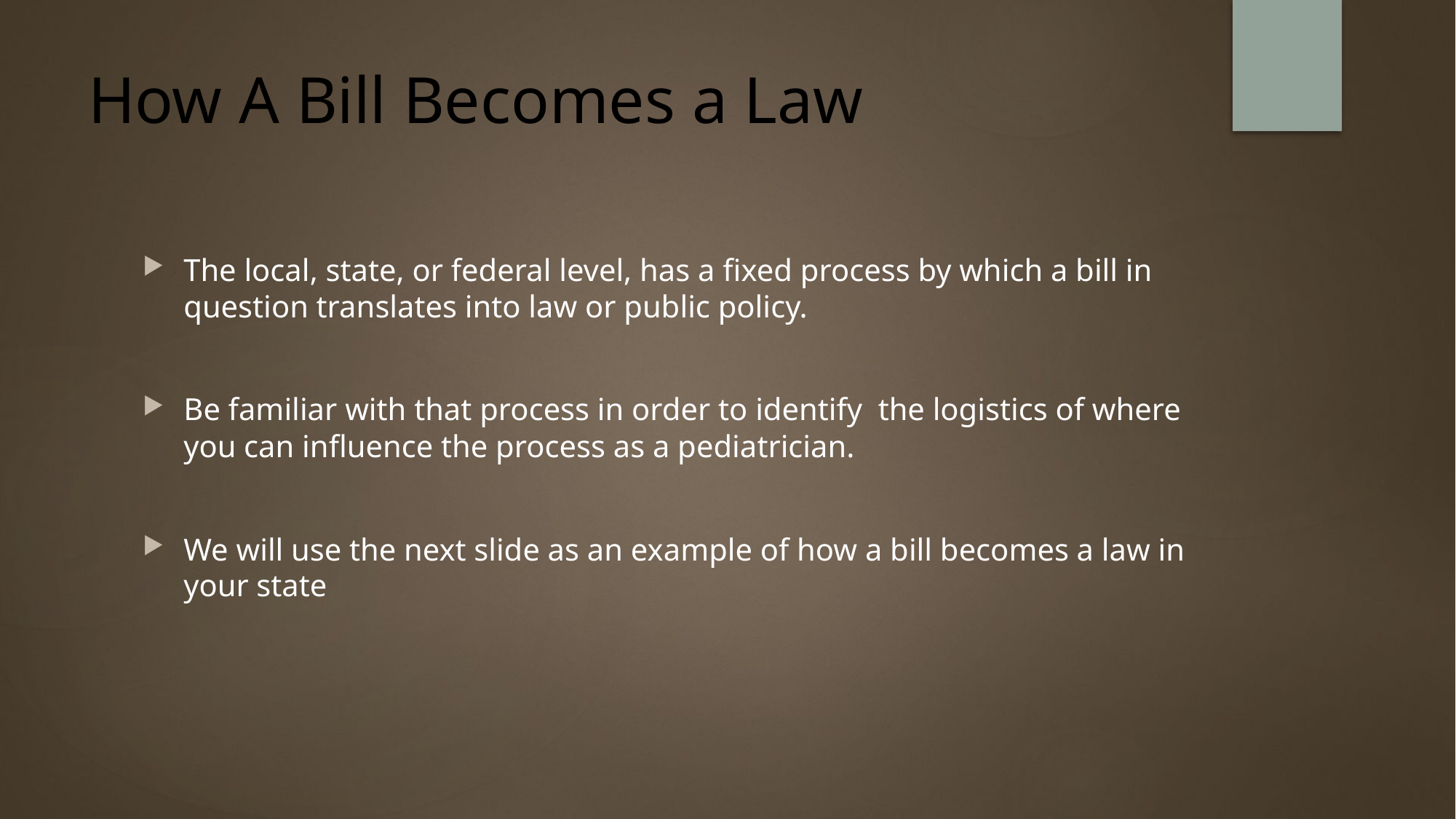

# How A Bill Becomes a Law
The local, state, or federal level, has a fixed process by which a bill in question translates into law or public policy.
Be familiar with that process in order to identify  the logistics of where you can influence the process as a pediatrician.
We will use the next slide as an example of how a bill becomes a law in your state

## Slide 11
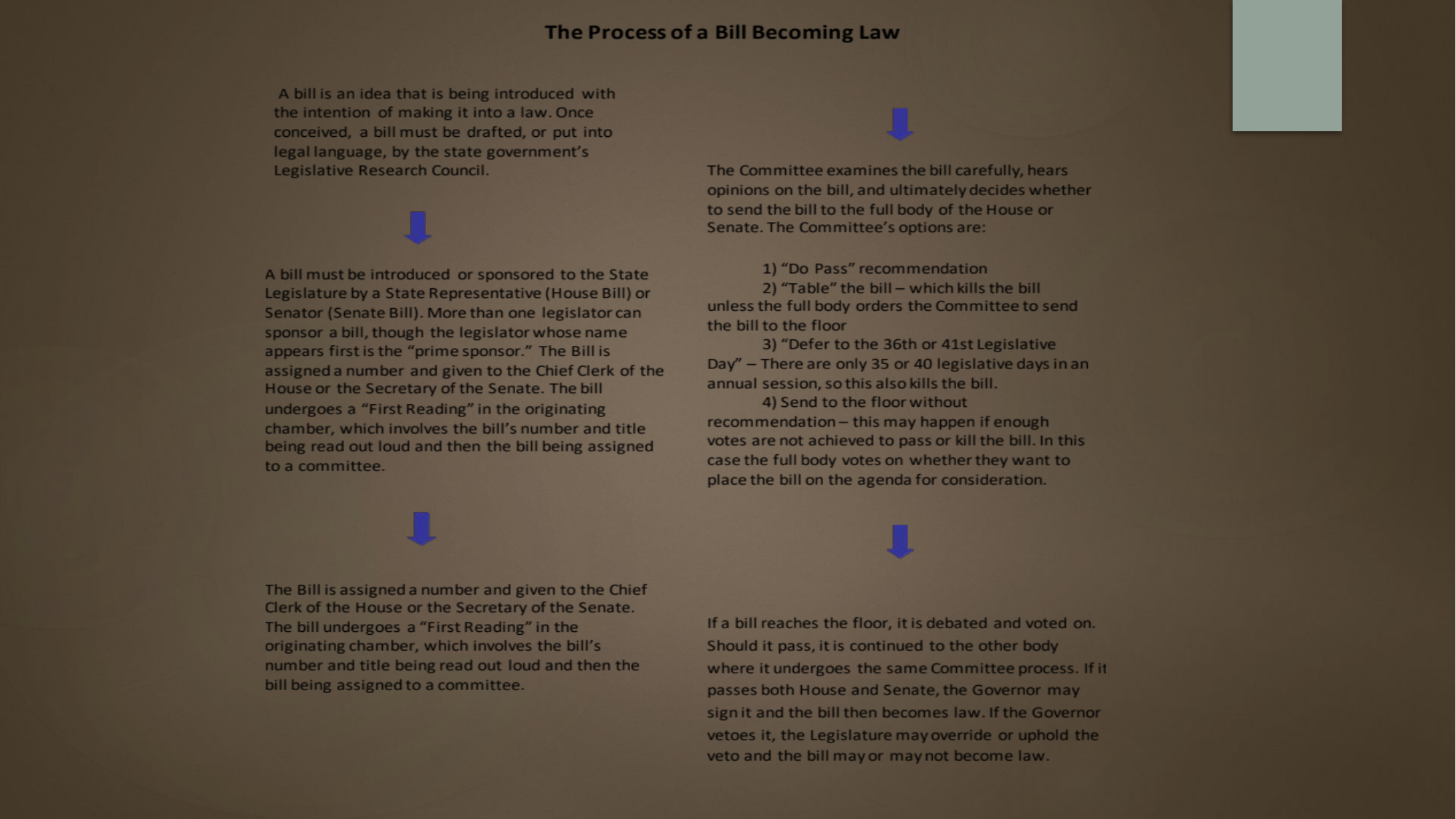

Supplement: Supplementary file 1 — A. Lecture 1.pptx B. Lecture 2.pptx C. Lecture 3.ppt D. Lecture 4.pptx E. Workshop 1.pptx F. Workshop 1 Skill Checklist.pdf G. Workshop 2.pptx H. Workshop 3.pptx I. Curriculum Survey.docx [file mep-16-10882-s001.zip › B. Lecture 2.pptx]
