# Supplementary material for: Advancing Advocacy: Implementation of a Child Health Advocacy Curriculum in a Pediatrics Residency Program
Source: MedEdPORTAL. 2020 Feb 14;16:10882. doi: 10.15766/mep_2374-8265.10882 (PMC7062538; doi:10.15766/mep_2374-8265.10882)
Supplement: Supplementary file 1 — A. Lecture 1.pptx B. Lecture 2.pptx C. Lecture 3.ppt D. Lecture 4.pptx E. Workshop 1.pptx F. Workshop 1 Skill Checklist.pdf G. Workshop 2.pptx H. Workshop 3.pptx I. Curriculum Survey.docx [file mep-16-10882-s001.zip › D. Lecture 4.pptx]

## Slide 1
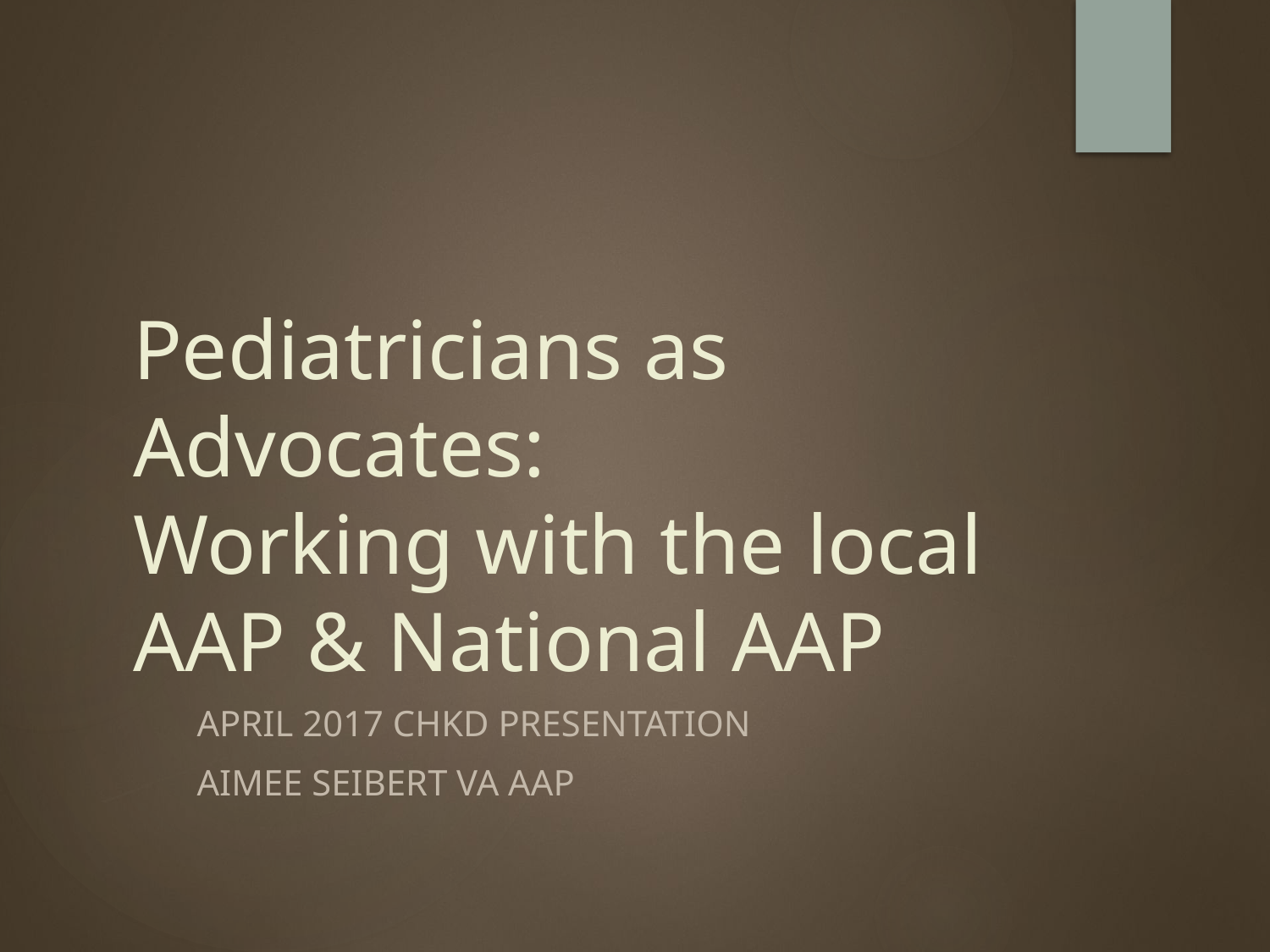

# Pediatricians as Advocates: Working with the local AAP & National AAP
April 2017 CHKD Presentation
Aimee Seibert VA AAP

## Slide 2
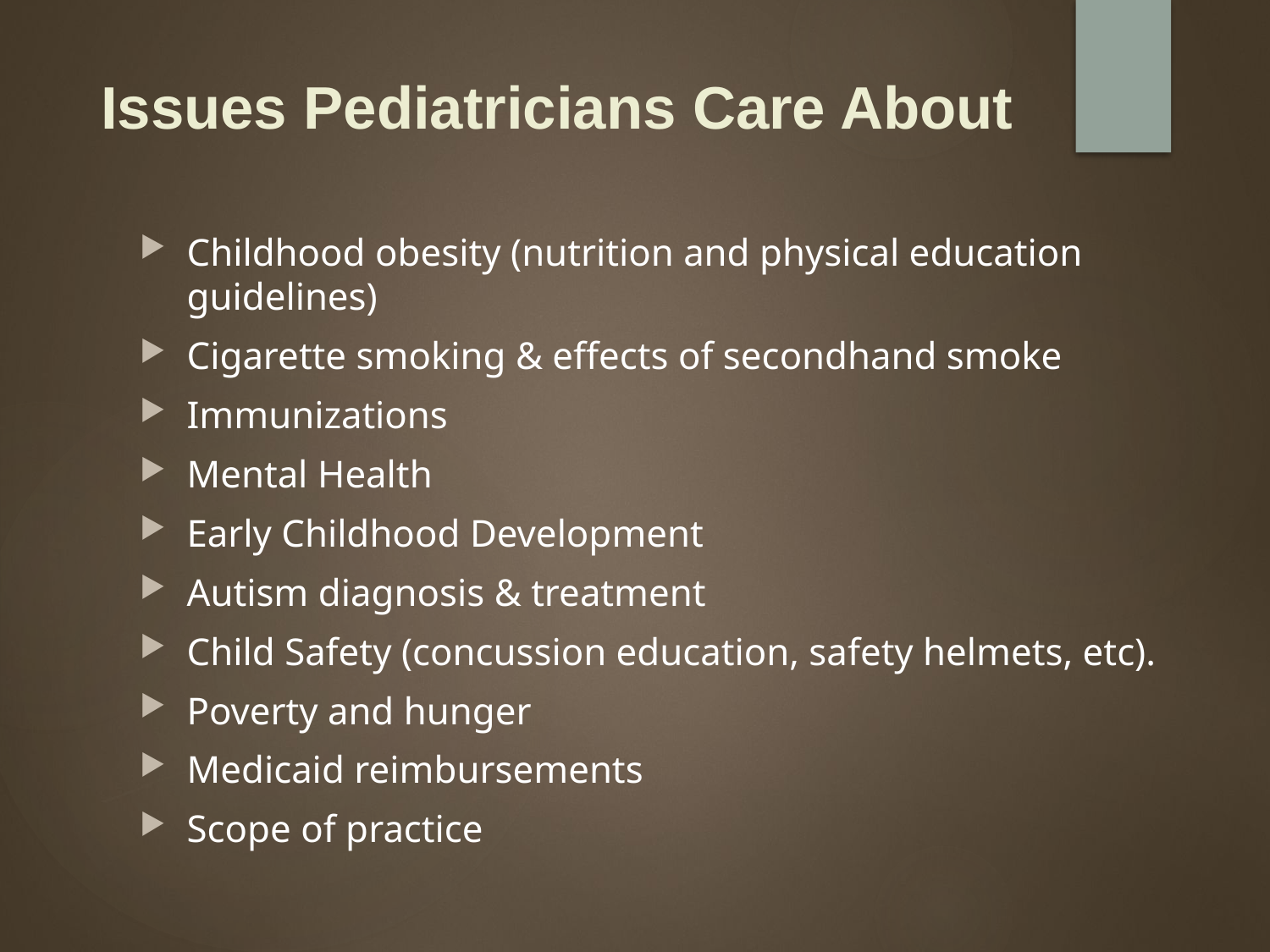

# Issues Pediatricians Care About
Childhood obesity (nutrition and physical education guidelines)
Cigarette smoking & effects of secondhand smoke
Immunizations
Mental Health
Early Childhood Development
Autism diagnosis & treatment
Child Safety (concussion education, safety helmets, etc).
Poverty and hunger
Medicaid reimbursements
Scope of practice

## Slide 3
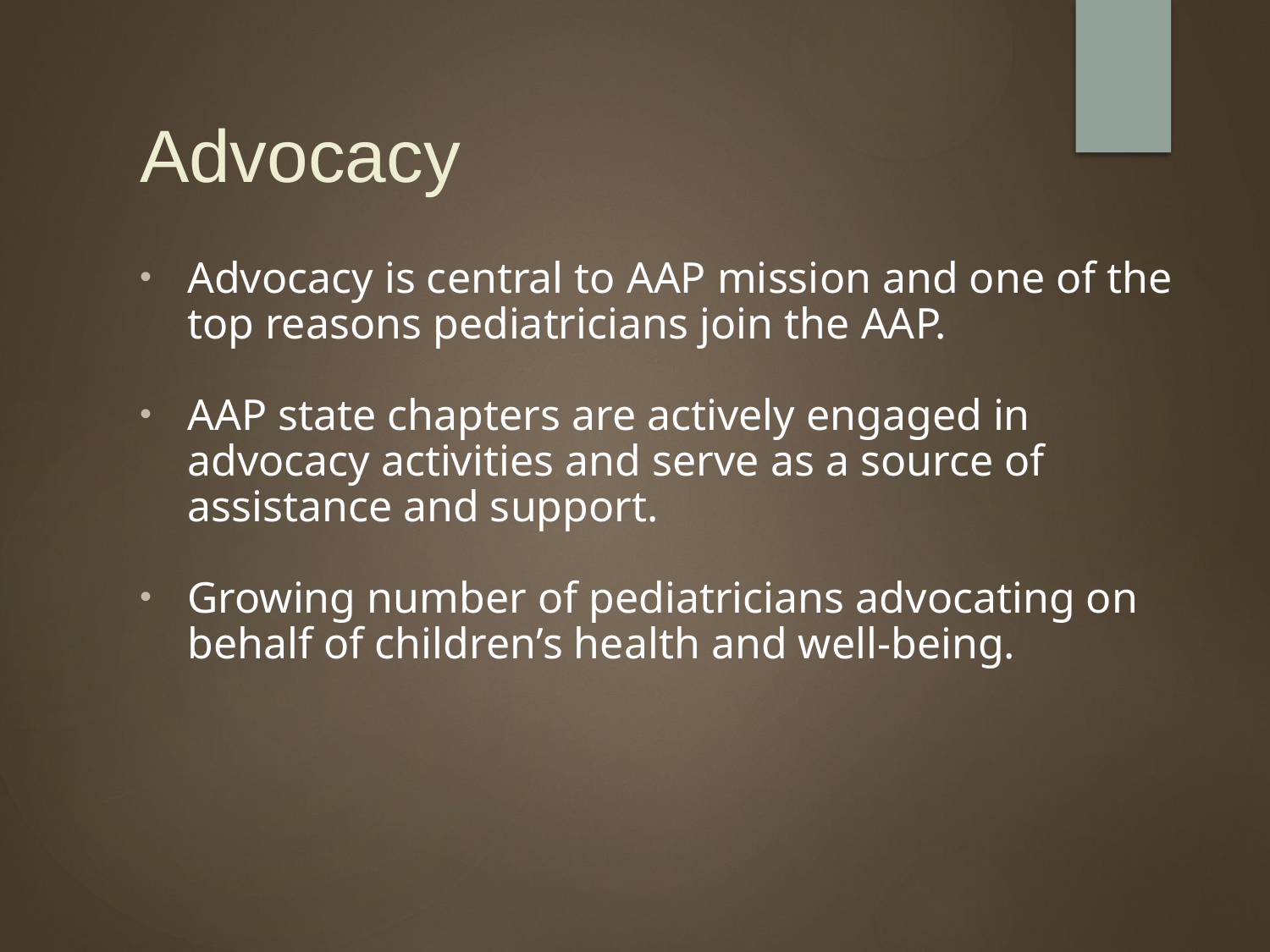

# Advocacy
Advocacy is central to AAP mission and one of the top reasons pediatricians join the AAP.
AAP state chapters are actively engaged in advocacy activities and serve as a source of assistance and support.
Growing number of pediatricians advocating on behalf of children’s health and well-being.

## Slide 4
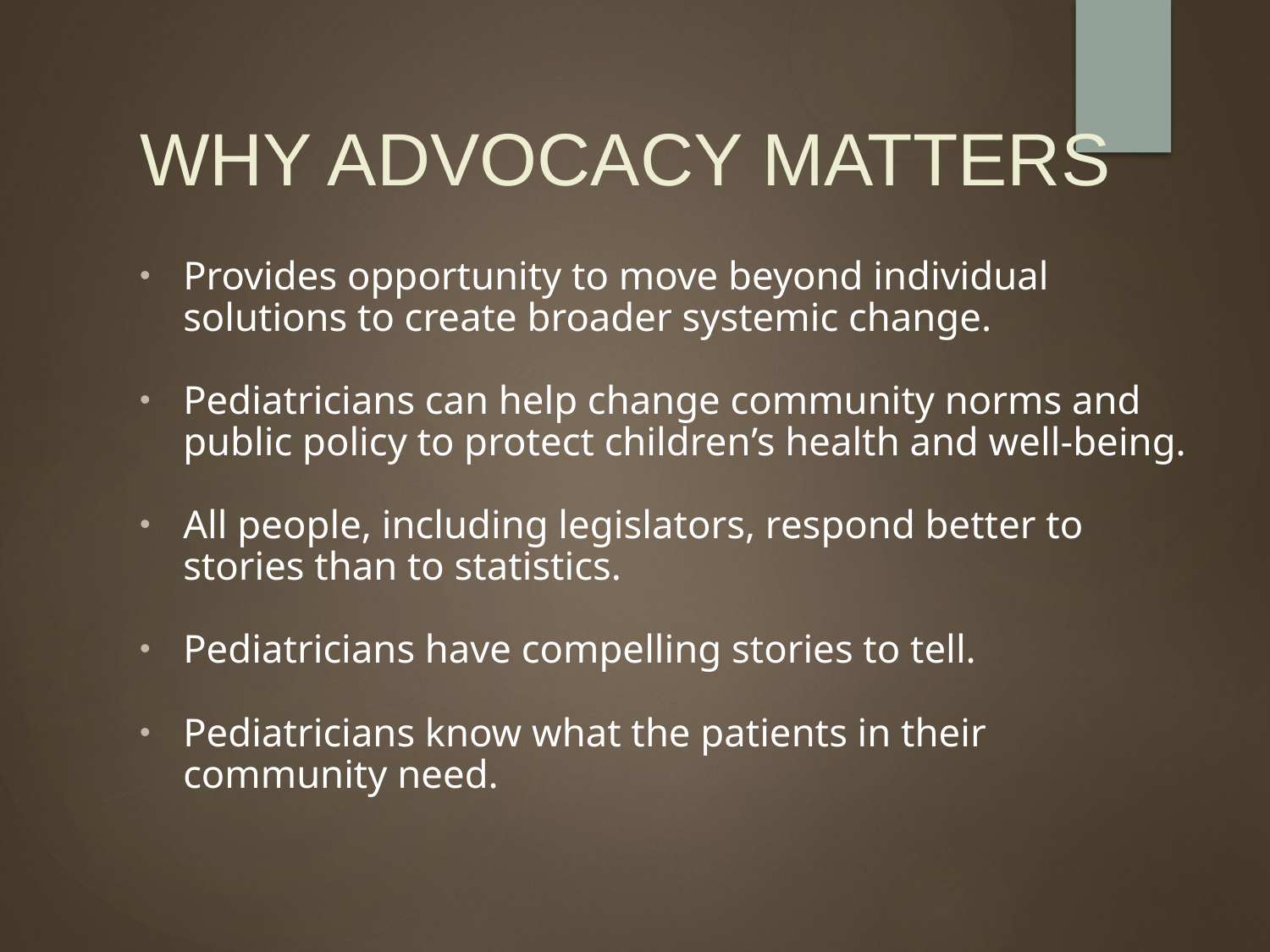

# WHY ADVOCACY MATTERS
Provides opportunity to move beyond individual solutions to create broader systemic change.
Pediatricians can help change community norms and public policy to protect children’s health and well-being.
All people, including legislators, respond better to stories than to statistics.
Pediatricians have compelling stories to tell.
Pediatricians know what the patients in their community need.

## Slide 5
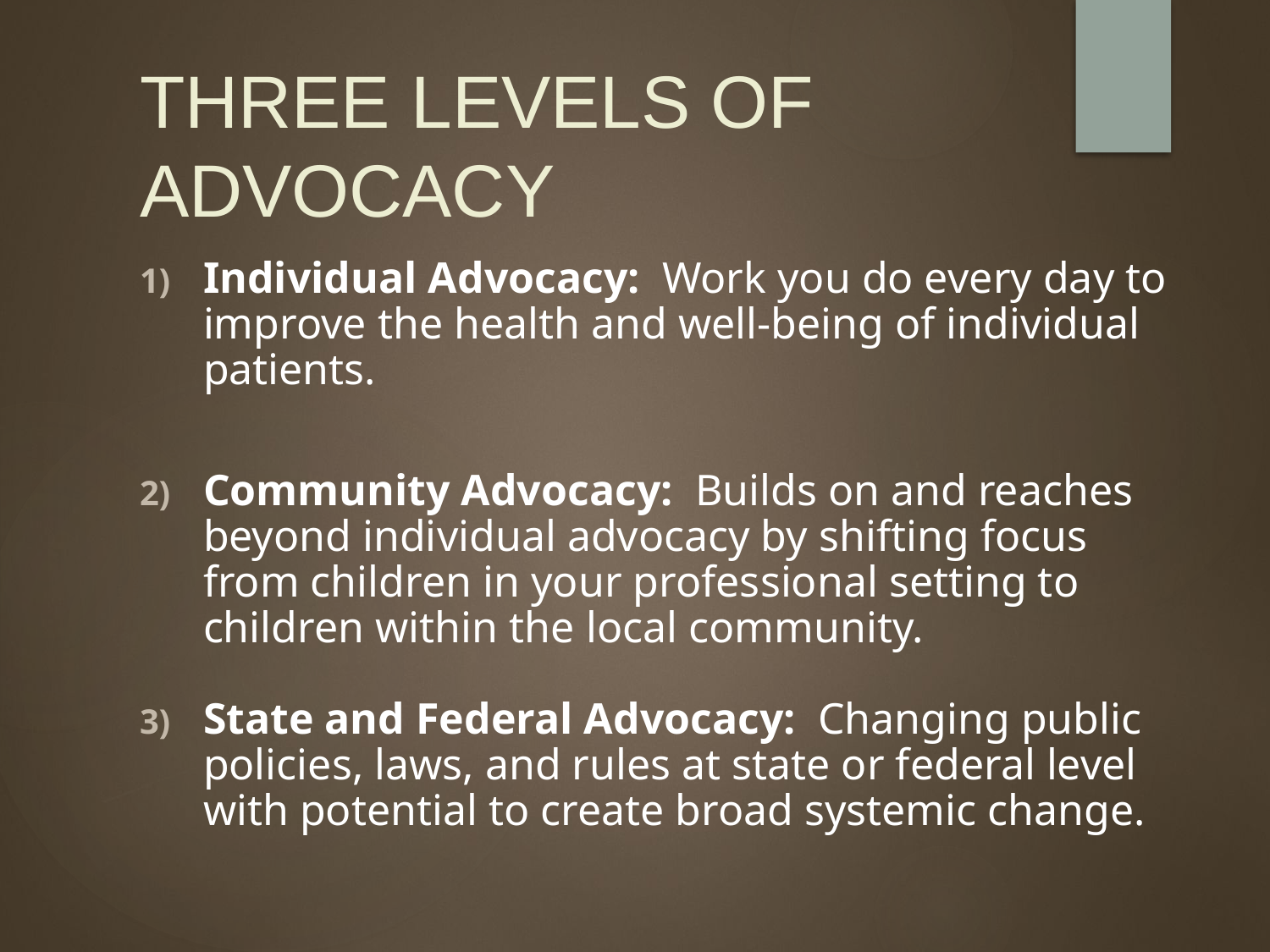

# THREE LEVELS OF ADVOCACY
Individual Advocacy: Work you do every day to improve the health and well-being of individual patients.
Community Advocacy: Builds on and reaches beyond individual advocacy by shifting focus from children in your professional setting to children within the local community.
State and Federal Advocacy: Changing public policies, laws, and rules at state or federal level with potential to create broad systemic change.

## Slide 6
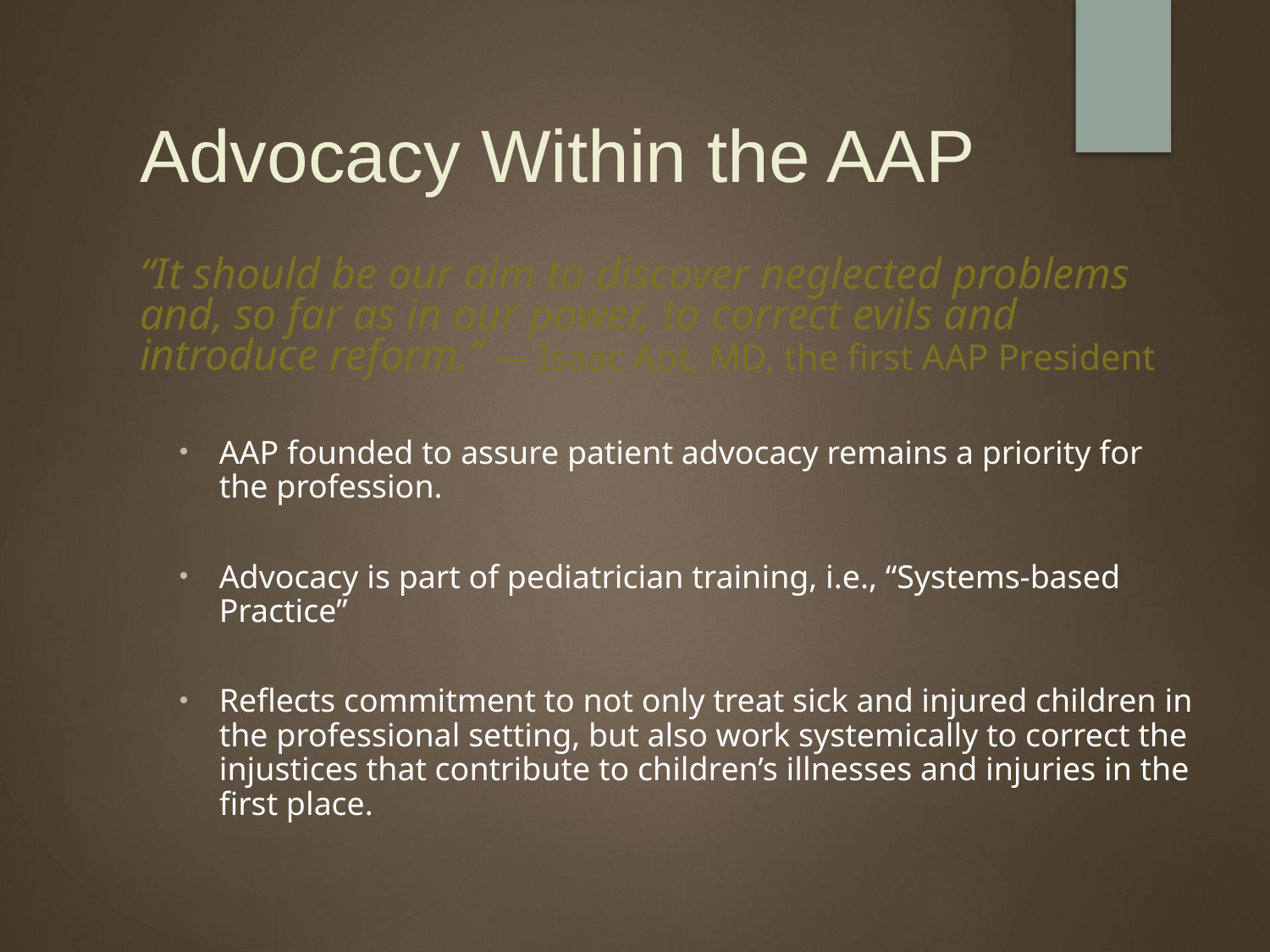

# Advocacy Within the AAP
“It should be our aim to discover neglected problems and, so far as in our power, to correct evils and introduce reform.” — Isaac Abt, MD, the first AAP President
AAP founded to assure patient advocacy remains a priority for the profession.
Advocacy is part of pediatrician training, i.e., “Systems-based Practice”
Reflects commitment to not only treat sick and injured children in the professional setting, but also work systemically to correct the injustices that contribute to children’s illnesses and injuries in the first place.

## Slide 7
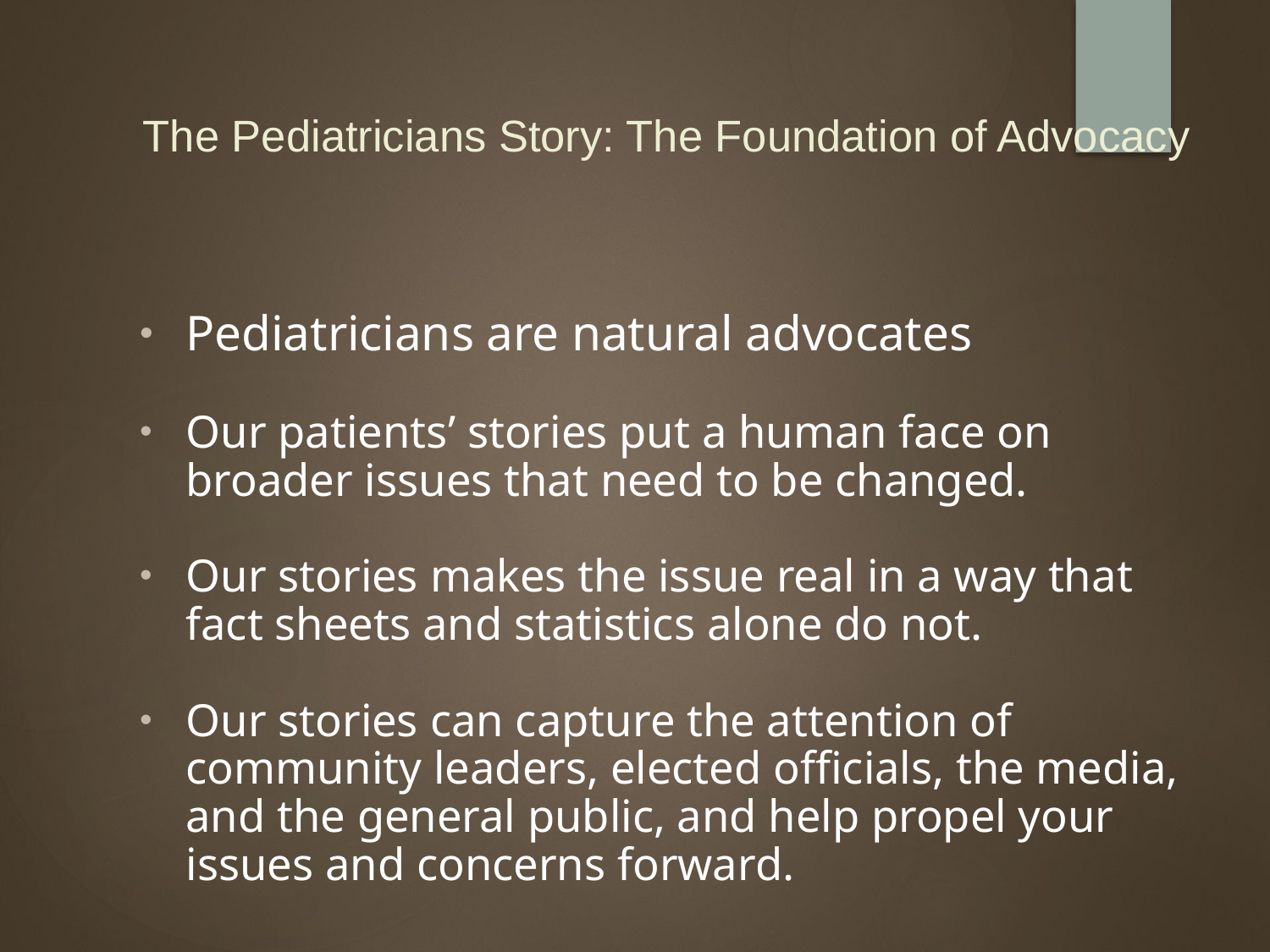

# The Pediatricians Story: The Foundation of Advocacy
Pediatricians are natural advocates
Our patients’ stories put a human face on broader issues that need to be changed.
Our stories makes the issue real in a way that fact sheets and statistics alone do not.
Our stories can capture the attention of community leaders, elected officials, the media, and the general public, and help propel your issues and concerns forward.

## Slide 8
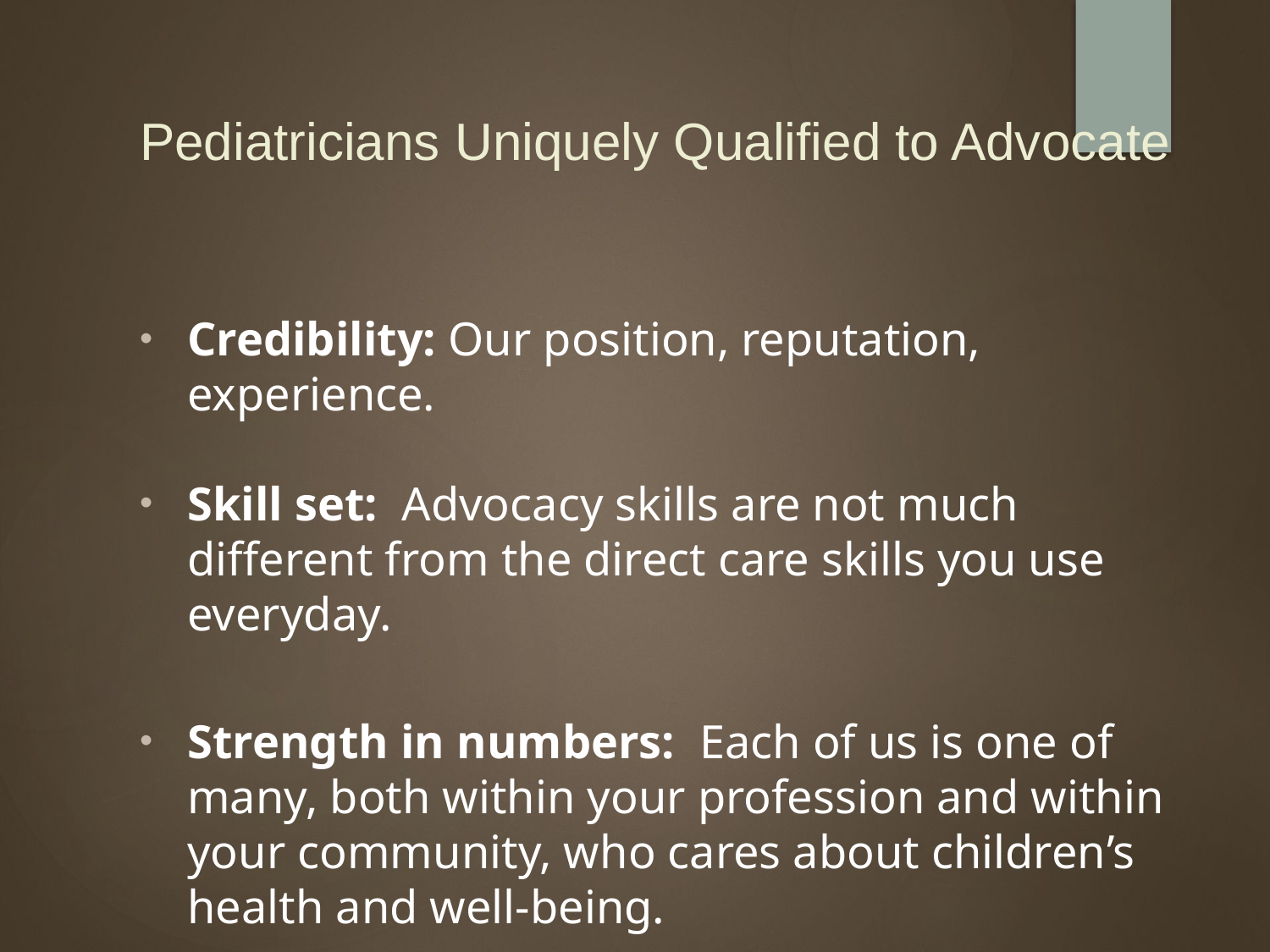

# Pediatricians Uniquely Qualified to Advocate
Credibility: Our position, reputation, experience.
Skill set: Advocacy skills are not much different from the direct care skills you use everyday.
Strength in numbers: Each of us is one of many, both within your profession and within your community, who cares about children’s health and well-being.

## Slide 9
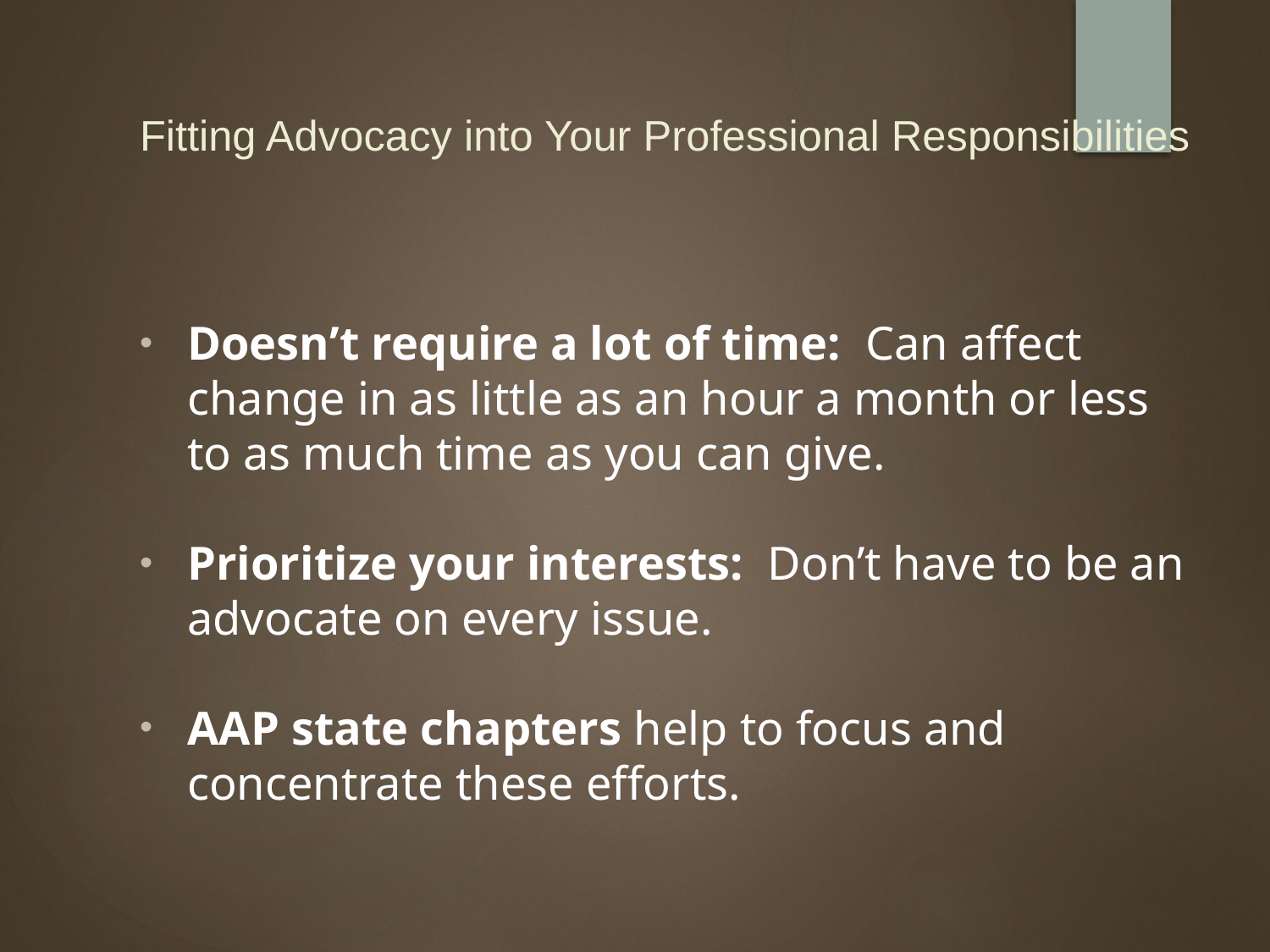

# Fitting Advocacy into Your Professional Responsibilities
Doesn’t require a lot of time: Can affect change in as little as an hour a month or less to as much time as you can give.
Prioritize your interests: Don’t have to be an advocate on every issue.
AAP state chapters help to focus and concentrate these efforts.

## Slide 10
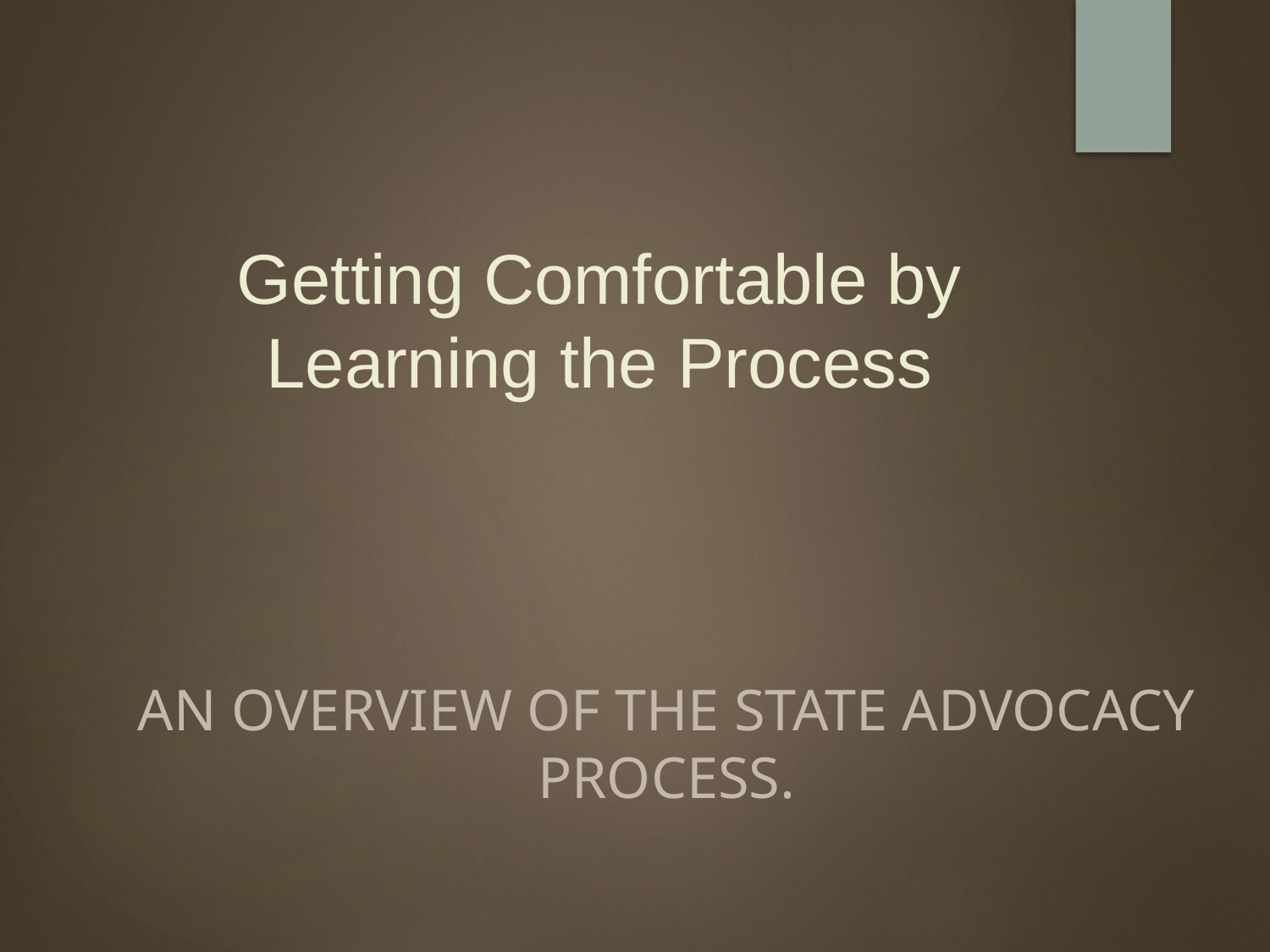

# Getting Comfortable by Learning the Process
An overview of the state advocacy process.

## Slide 11
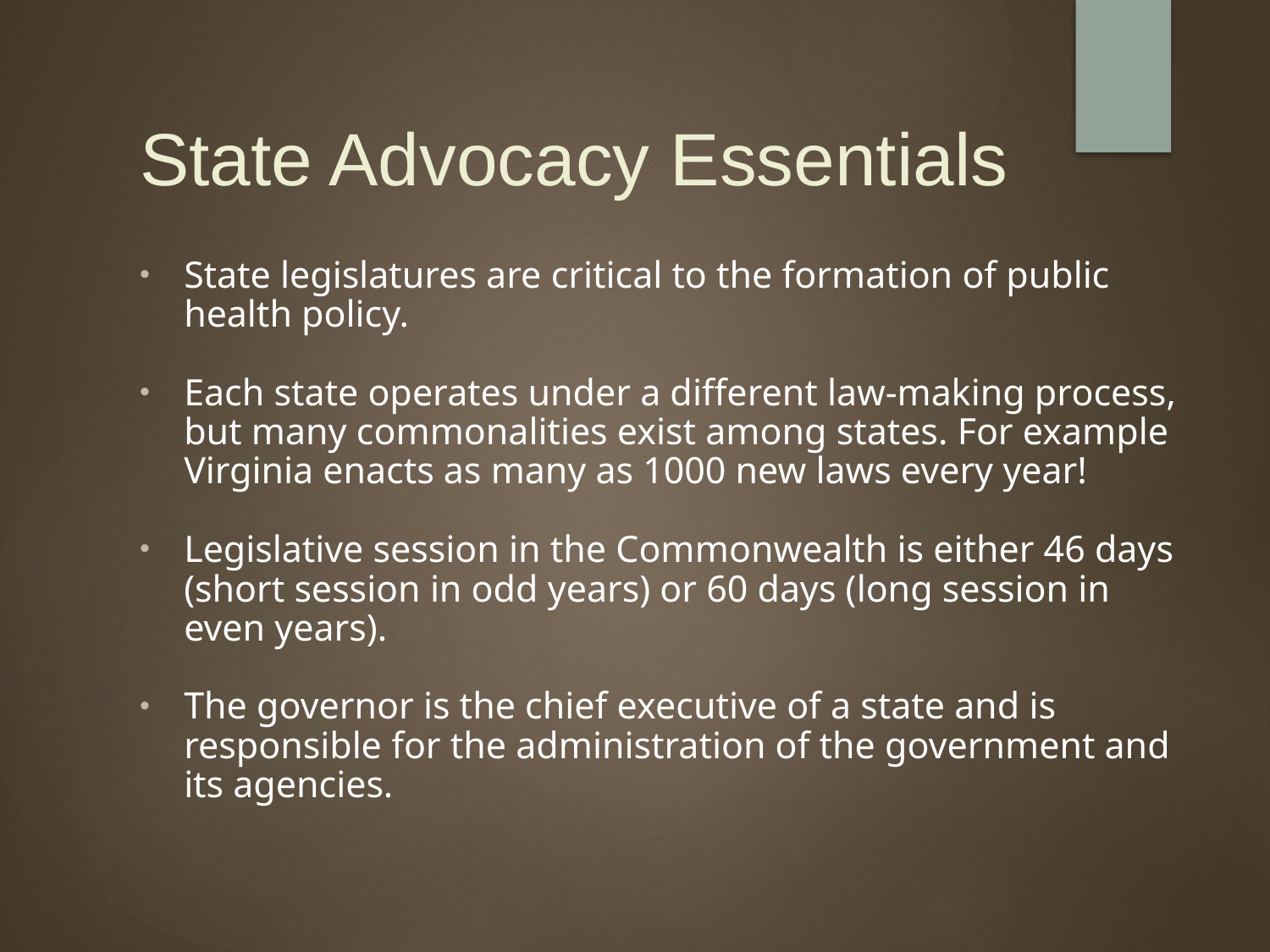

# State Advocacy Essentials
State legislatures are critical to the formation of public health policy.
Each state operates under a different law-making process, but many commonalities exist among states. For example Virginia enacts as many as 1000 new laws every year!
Legislative session in the Commonwealth is either 46 days (short session in odd years) or 60 days (long session in even years).
The governor is the chief executive of a state and is responsible for the administration of the government and its agencies.

## Slide 12
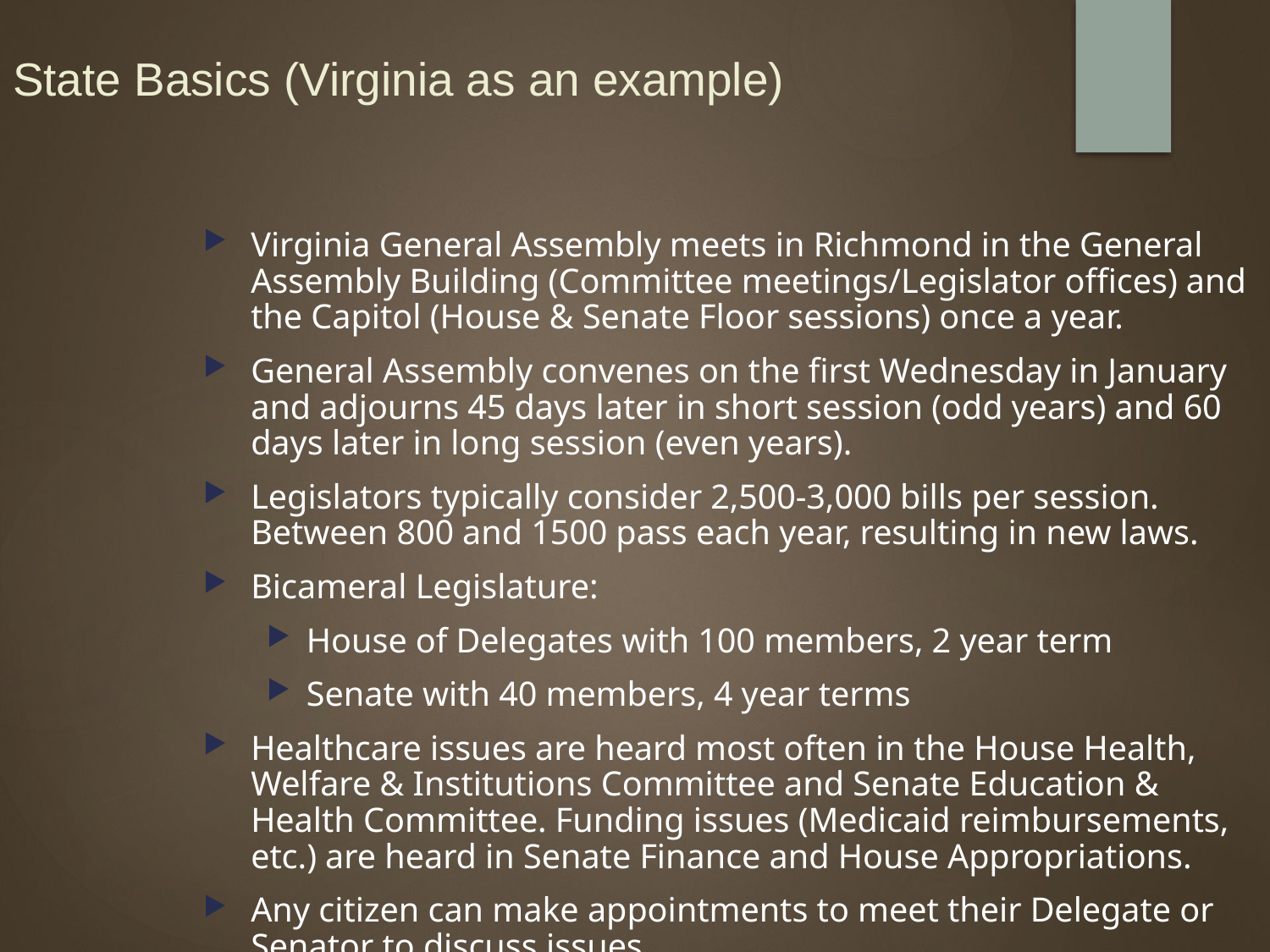

State Basics (Virginia as an example)
Virginia General Assembly meets in Richmond in the General Assembly Building (Committee meetings/Legislator offices) and the Capitol (House & Senate Floor sessions) once a year.
General Assembly convenes on the first Wednesday in January and adjourns 45 days later in short session (odd years) and 60 days later in long session (even years).
Legislators typically consider 2,500-3,000 bills per session. Between 800 and 1500 pass each year, resulting in new laws.
Bicameral Legislature:
House of Delegates with 100 members, 2 year term
Senate with 40 members, 4 year terms
Healthcare issues are heard most often in the House Health, Welfare & Institutions Committee and Senate Education & Health Committee. Funding issues (Medicaid reimbursements, etc.) are heard in Senate Finance and House Appropriations.
Any citizen can make appointments to meet their Delegate or Senator to discuss issues.

## Slide 13
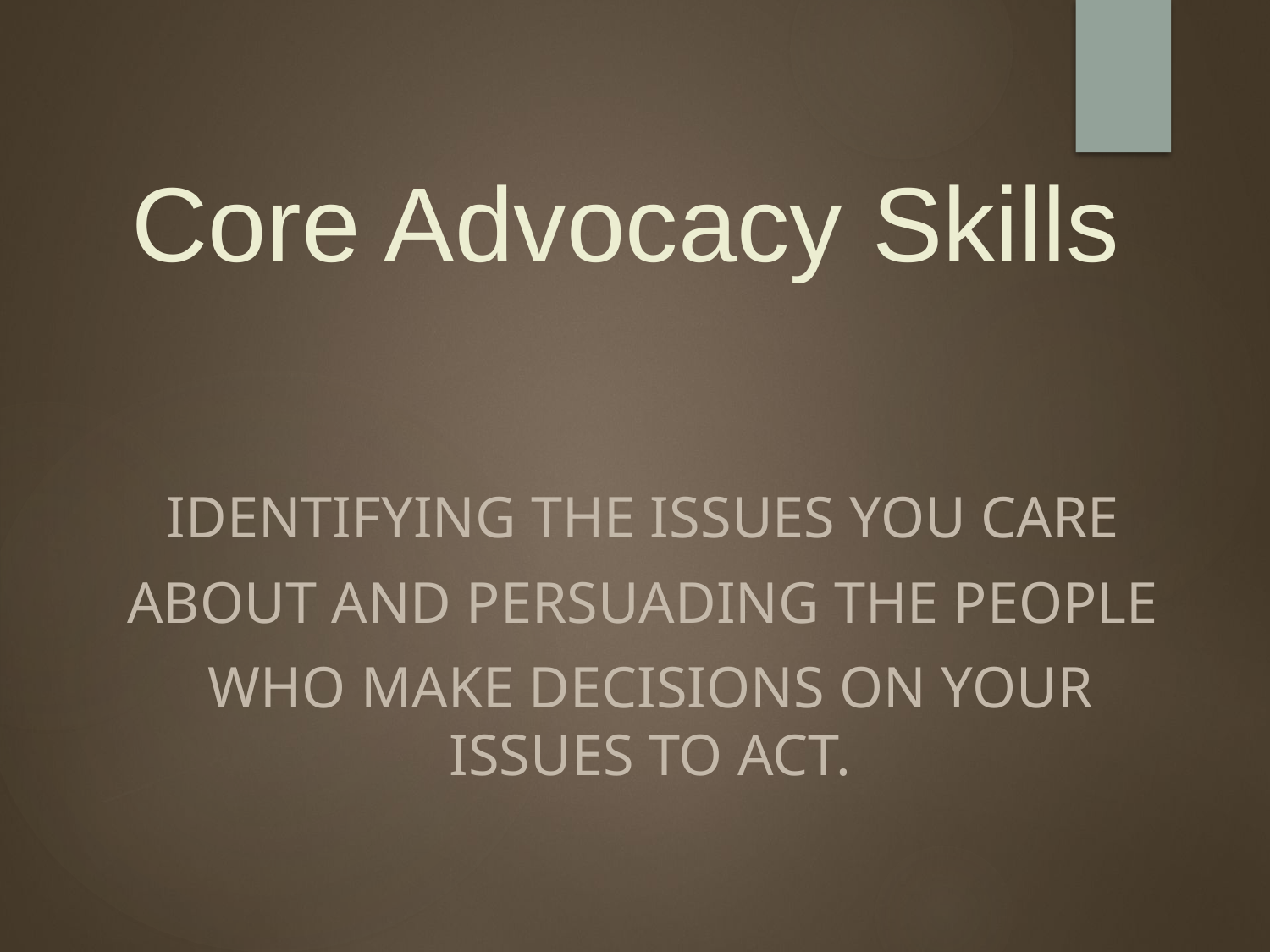

# Core Advocacy Skills
Identifying the issues you care
about and persuading the people
who make decisions on your issues to act.

## Slide 14
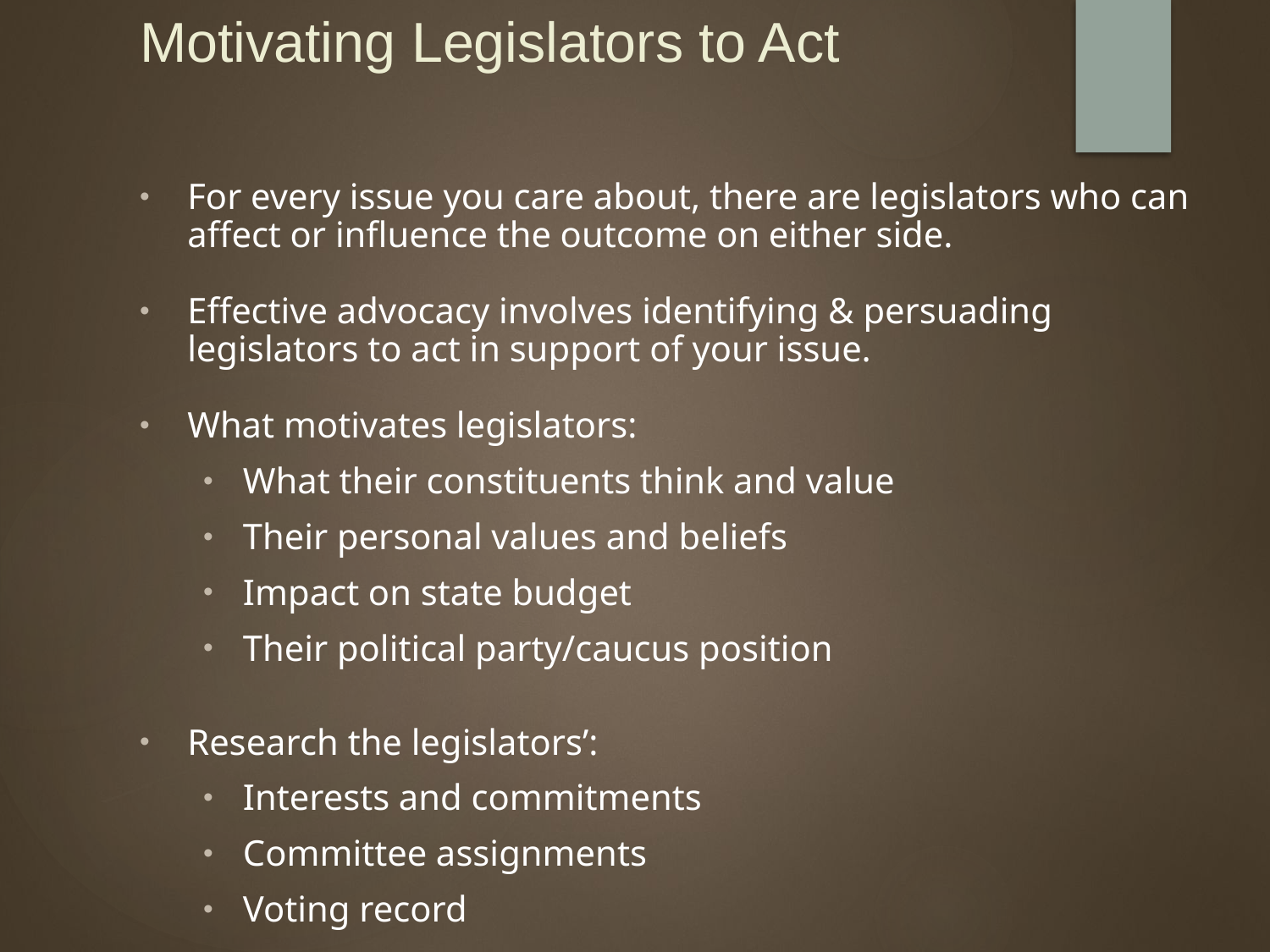

# Motivating Legislators to Act
For every issue you care about, there are legislators who can affect or influence the outcome on either side.
Effective advocacy involves identifying & persuading legislators to act in support of your issue.
What motivates legislators:
What their constituents think and value
Their personal values and beliefs
Impact on state budget
Their political party/caucus position
Research the legislators’:
Interests and commitments
Committee assignments
Voting record

## Slide 15
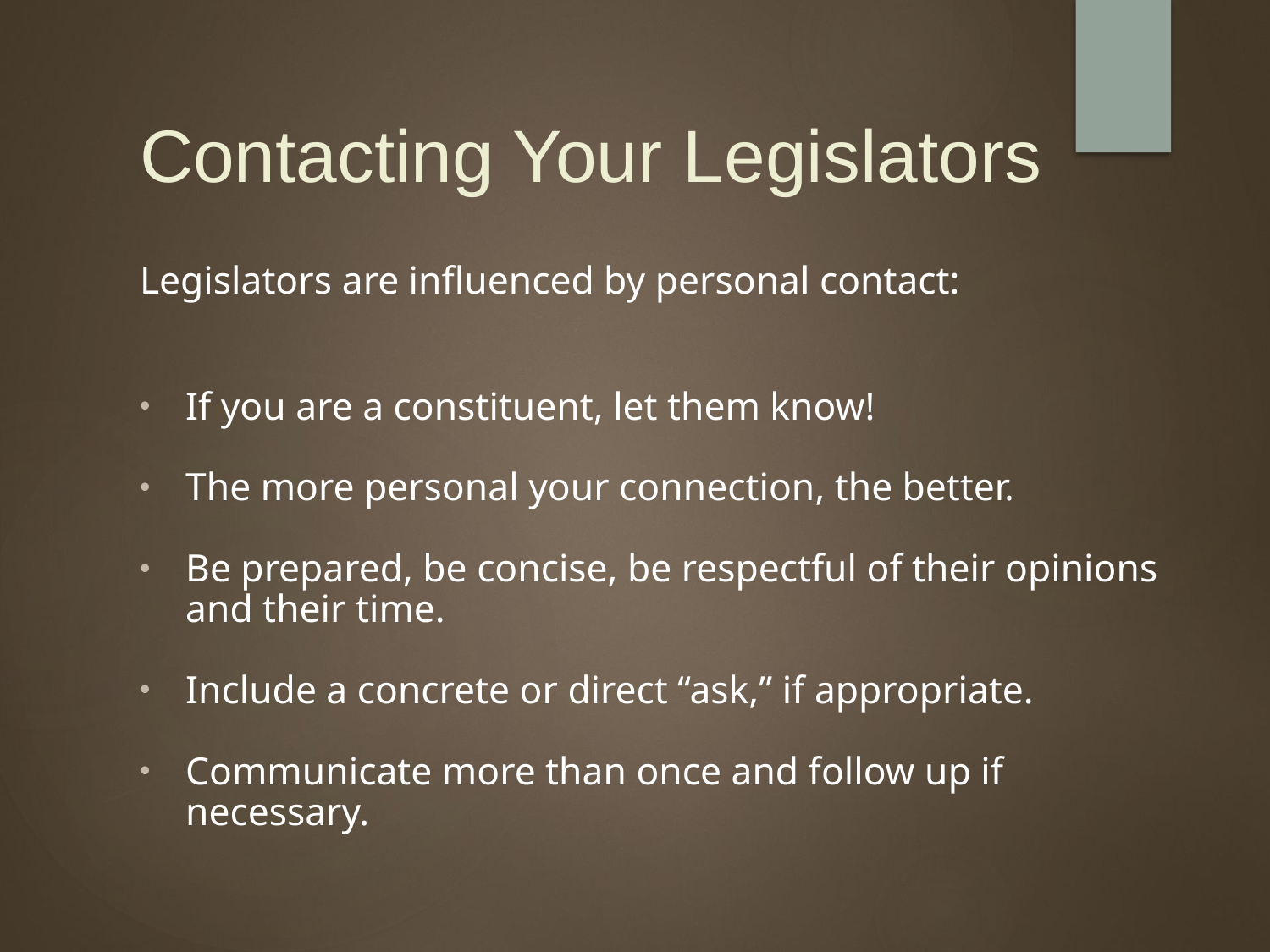

# Contacting Your Legislators
Legislators are influenced by personal contact:
If you are a constituent, let them know!
The more personal your connection, the better.
Be prepared, be concise, be respectful of their opinions and their time.
Include a concrete or direct “ask,” if appropriate.
Communicate more than once and follow up if necessary.

## Slide 16
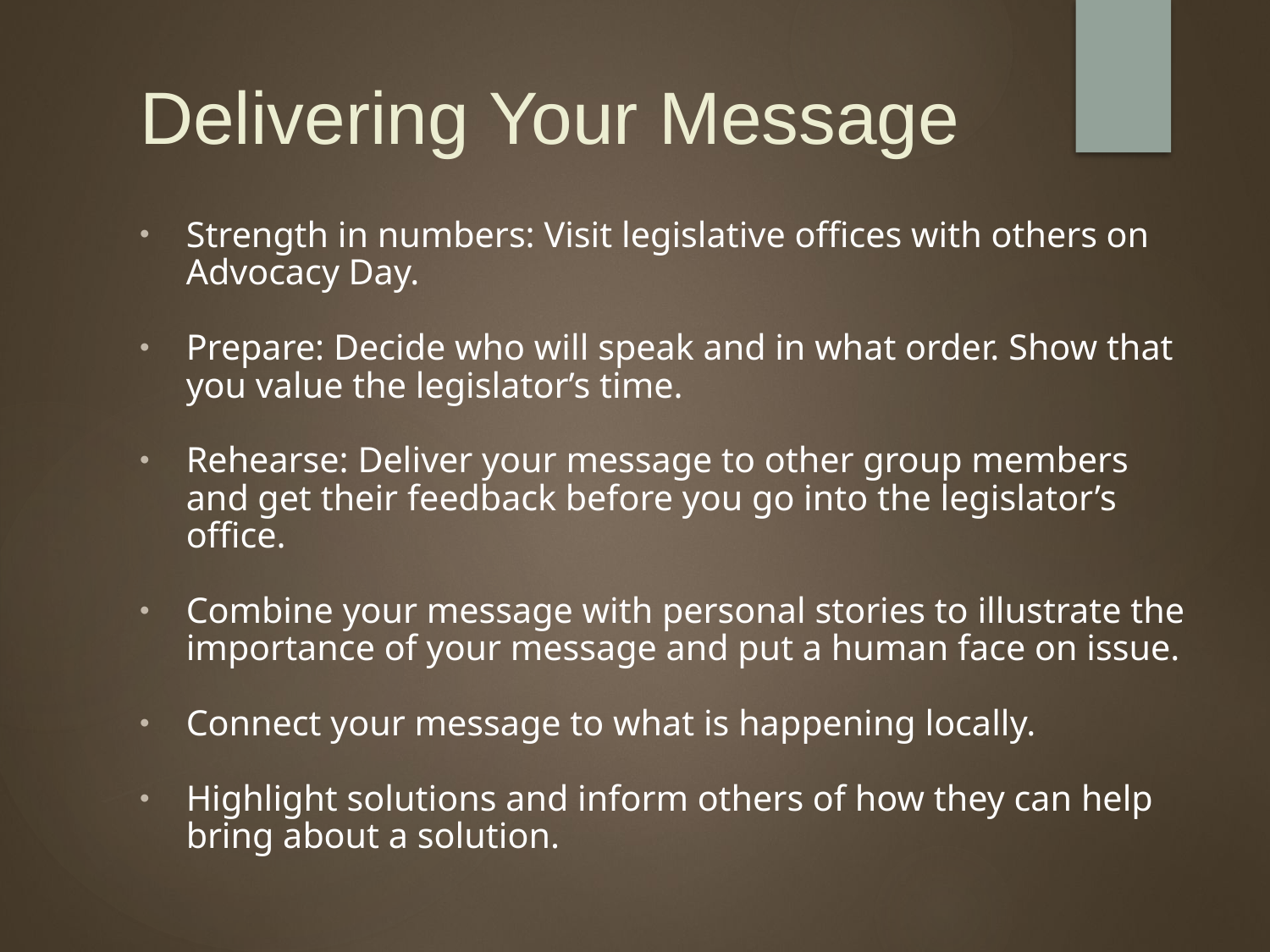

# Delivering Your Message
Strength in numbers: Visit legislative offices with others on Advocacy Day.
Prepare: Decide who will speak and in what order. Show that you value the legislator’s time.
Rehearse: Deliver your message to other group members and get their feedback before you go into the legislator’s office.
Combine your message with personal stories to illustrate the importance of your message and put a human face on issue.
Connect your message to what is happening locally.
Highlight solutions and inform others of how they can help bring about a solution.

## Slide 17
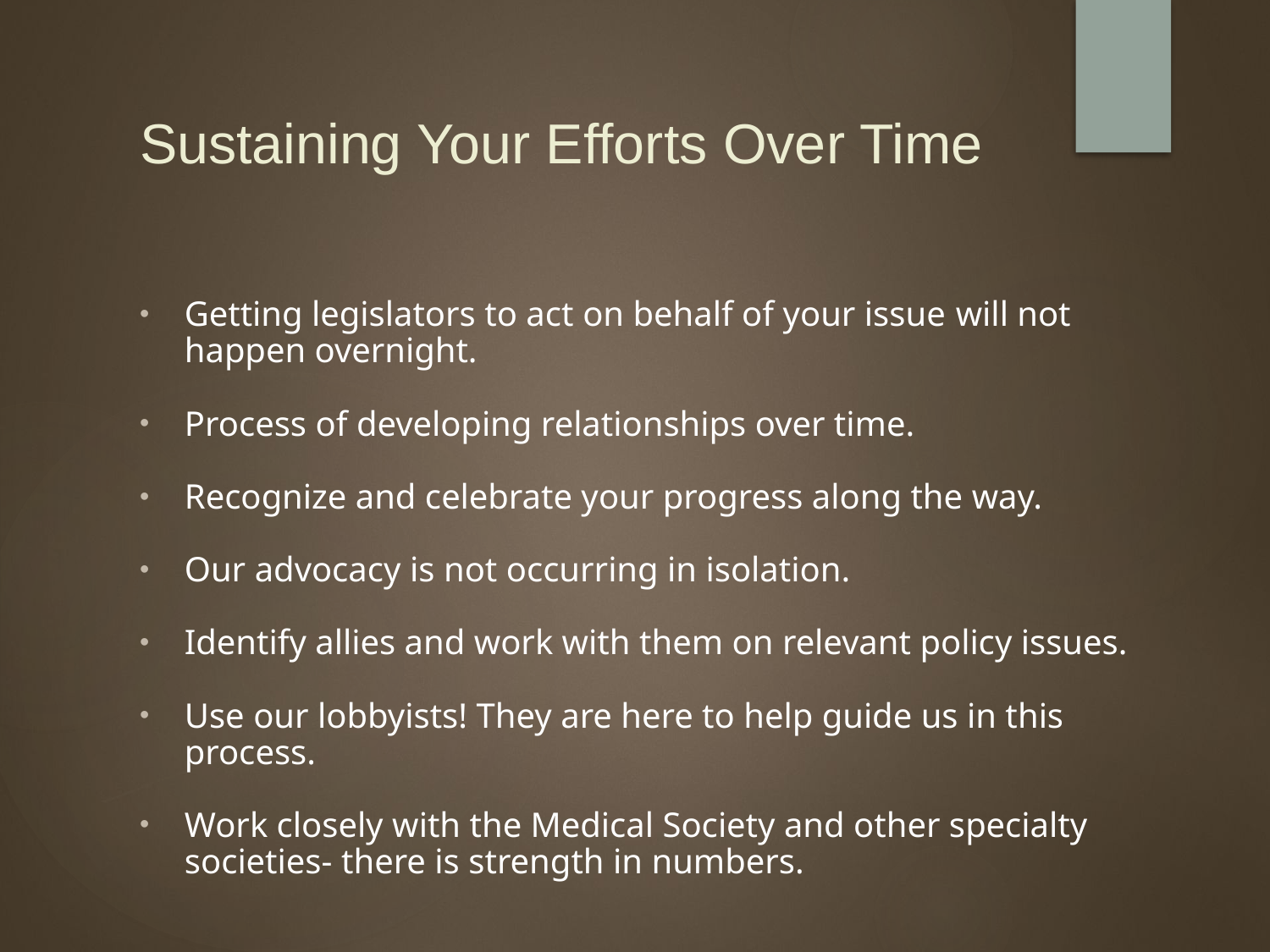

# Sustaining Your Efforts Over Time
Getting legislators to act on behalf of your issue will not happen overnight.
Process of developing relationships over time.
Recognize and celebrate your progress along the way.
Our advocacy is not occurring in isolation.
Identify allies and work with them on relevant policy issues.
Use our lobbyists! They are here to help guide us in this process.
Work closely with the Medical Society and other specialty societies- there is strength in numbers.

## Slide 18
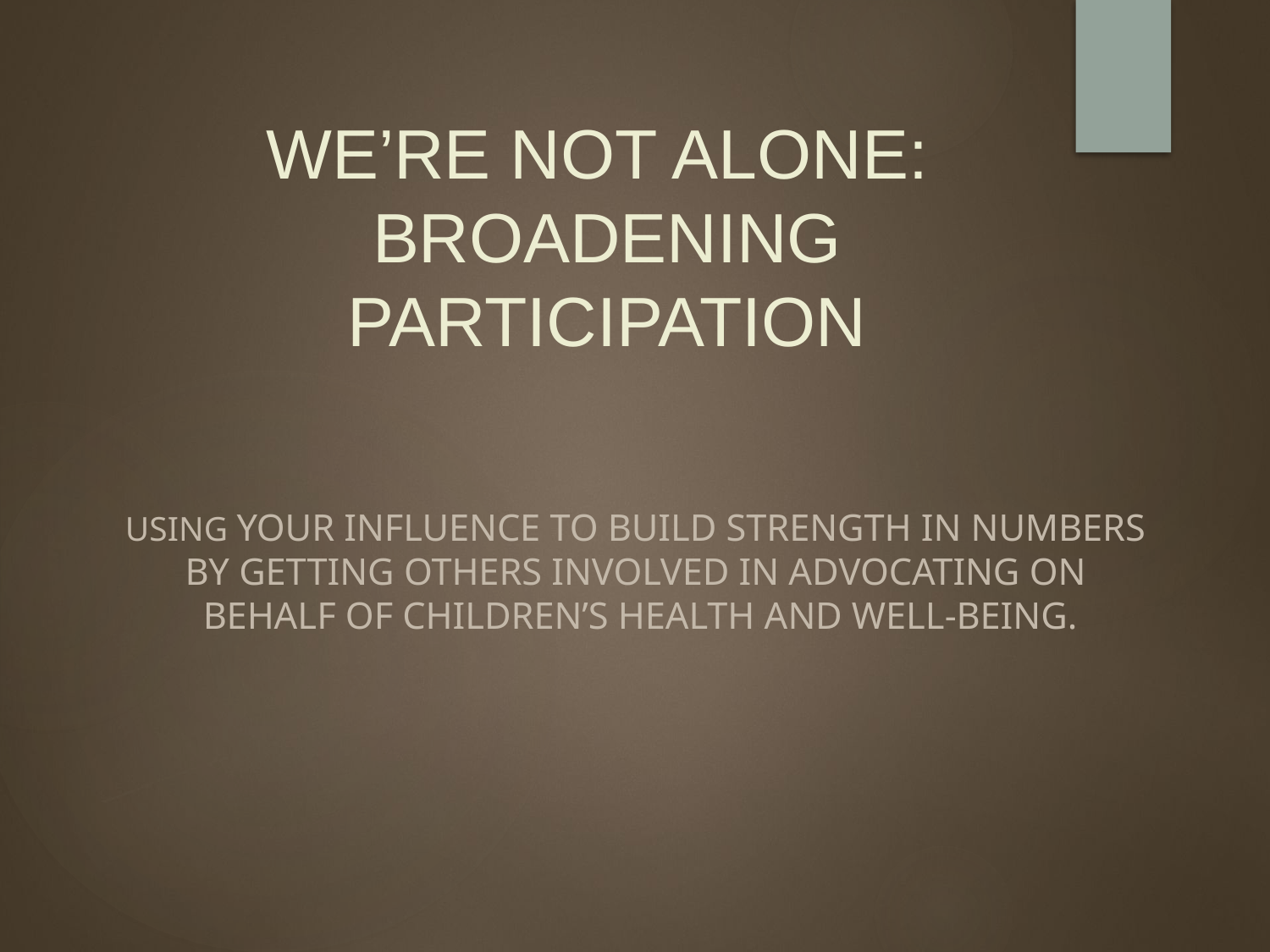

# WE’RE NOT ALONE: BROADENING PARTICIPATION
Using your influence to build strength in numbers
by getting others involved in advocating on
behalf of children’s health and well-being.

## Slide 19
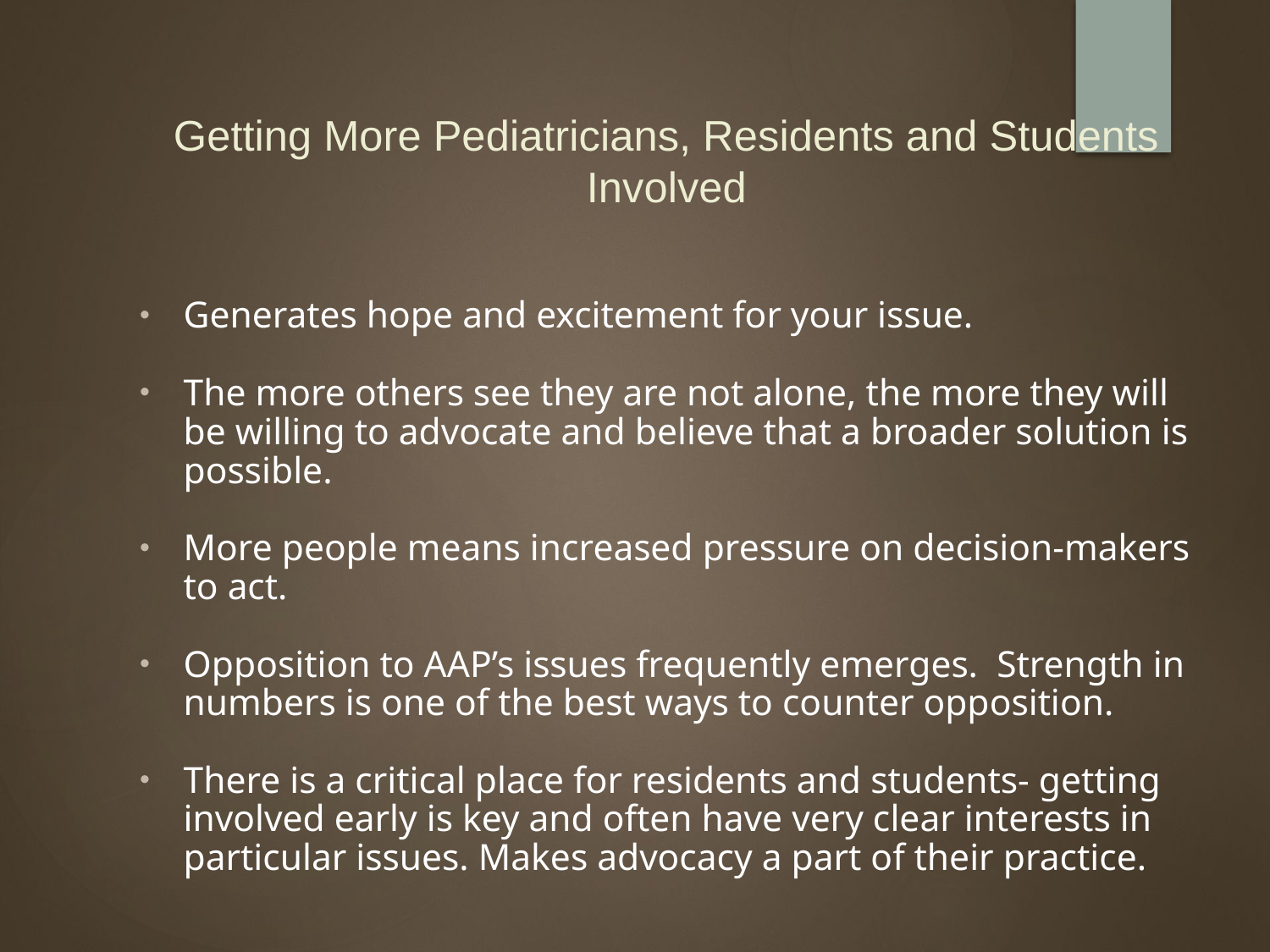

# Getting More Pediatricians, Residents and Students Involved
Generates hope and excitement for your issue.
The more others see they are not alone, the more they will be willing to advocate and believe that a broader solution is possible.
More people means increased pressure on decision-makers to act.
Opposition to AAP’s issues frequently emerges. Strength in numbers is one of the best ways to counter opposition.
There is a critical place for residents and students- getting involved early is key and often have very clear interests in particular issues. Makes advocacy a part of their practice.

## Slide 20
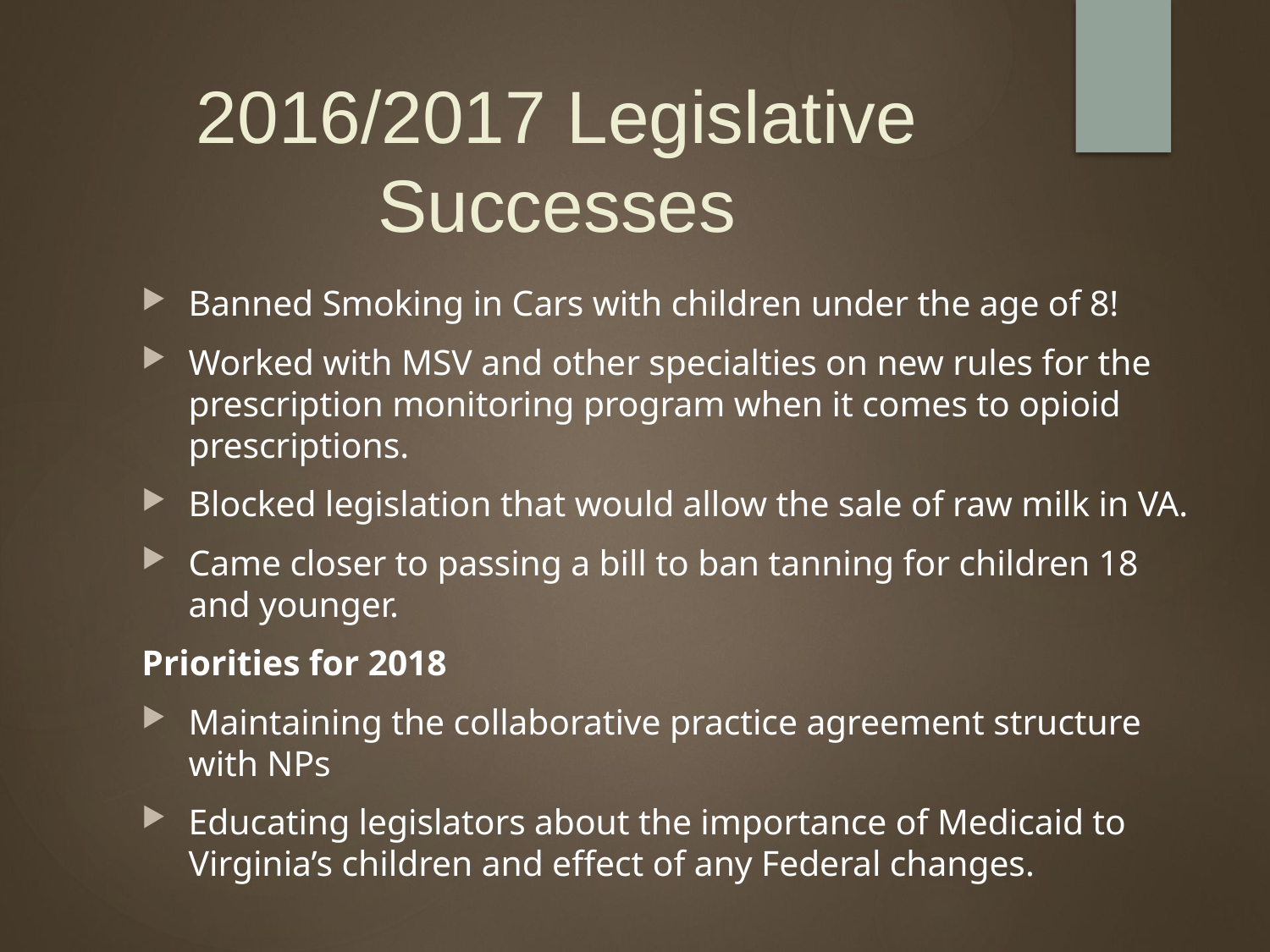

# 2016/2017 Legislative Successes
Banned Smoking in Cars with children under the age of 8!
Worked with MSV and other specialties on new rules for the prescription monitoring program when it comes to opioid prescriptions.
Blocked legislation that would allow the sale of raw milk in VA.
Came closer to passing a bill to ban tanning for children 18 and younger.
Priorities for 2018
Maintaining the collaborative practice agreement structure with NPs
Educating legislators about the importance of Medicaid to Virginia’s children and effect of any Federal changes.
