# Supplementary material for: Advancing Advocacy: Implementation of a Child Health Advocacy Curriculum in a Pediatrics Residency Program
Source: MedEdPORTAL. 2020 Feb 14;16:10882. doi: 10.15766/mep_2374-8265.10882 (PMC7062538; doi:10.15766/mep_2374-8265.10882)
Supplement: Supplementary file 1 — A. Lecture 1.pptx B. Lecture 2.pptx C. Lecture 3.ppt D. Lecture 4.pptx E. Workshop 1.pptx F. Workshop 1 Skill Checklist.pdf G. Workshop 2.pptx H. Workshop 3.pptx I. Curriculum Survey.docx [file mep-16-10882-s001.zip › E. Workshop 1.pptx]

## Slide 1
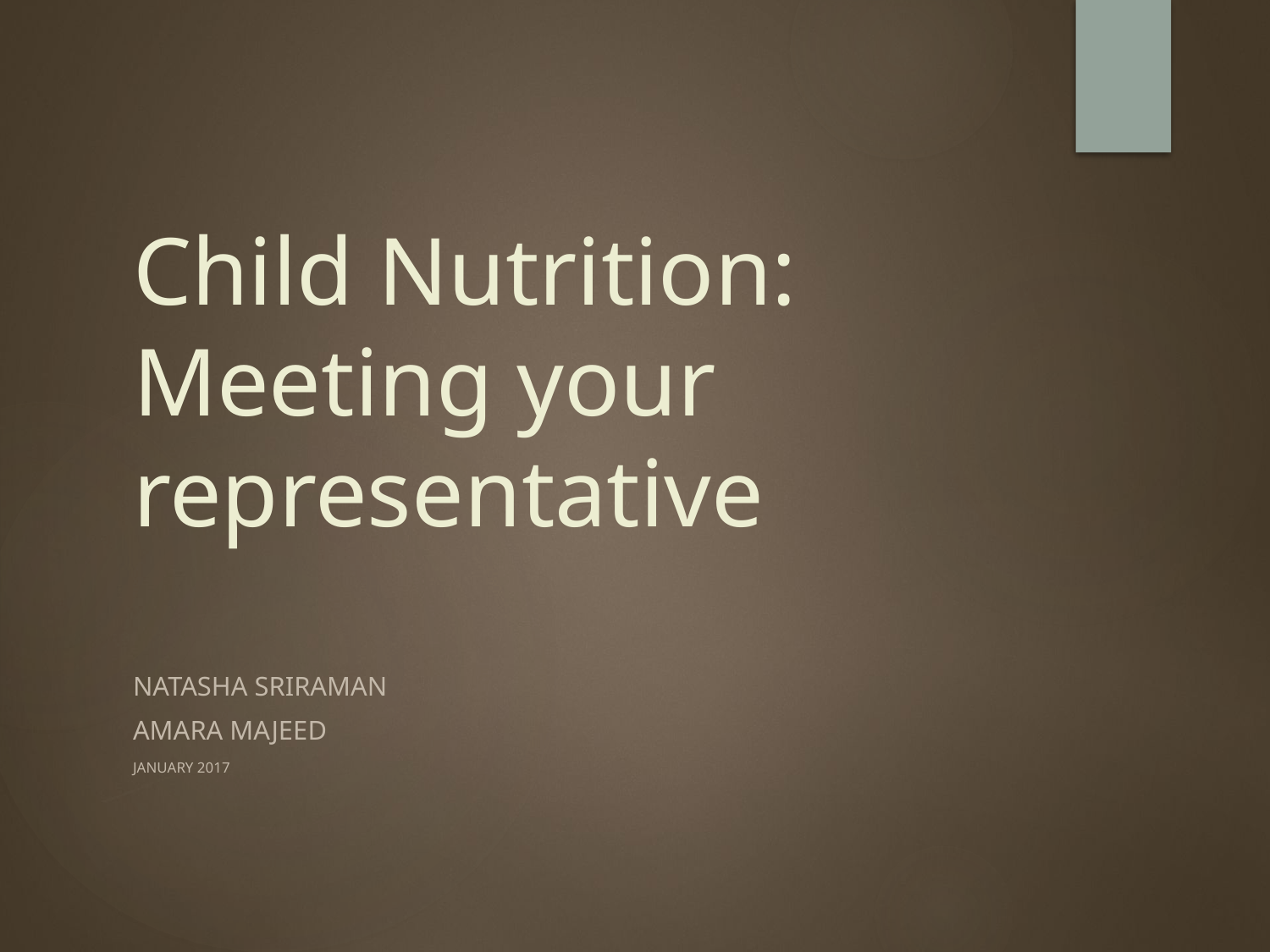

# Child Nutrition: Meeting your representative
Natasha Sriraman
Amara Majeed
January 2017

## Slide 2
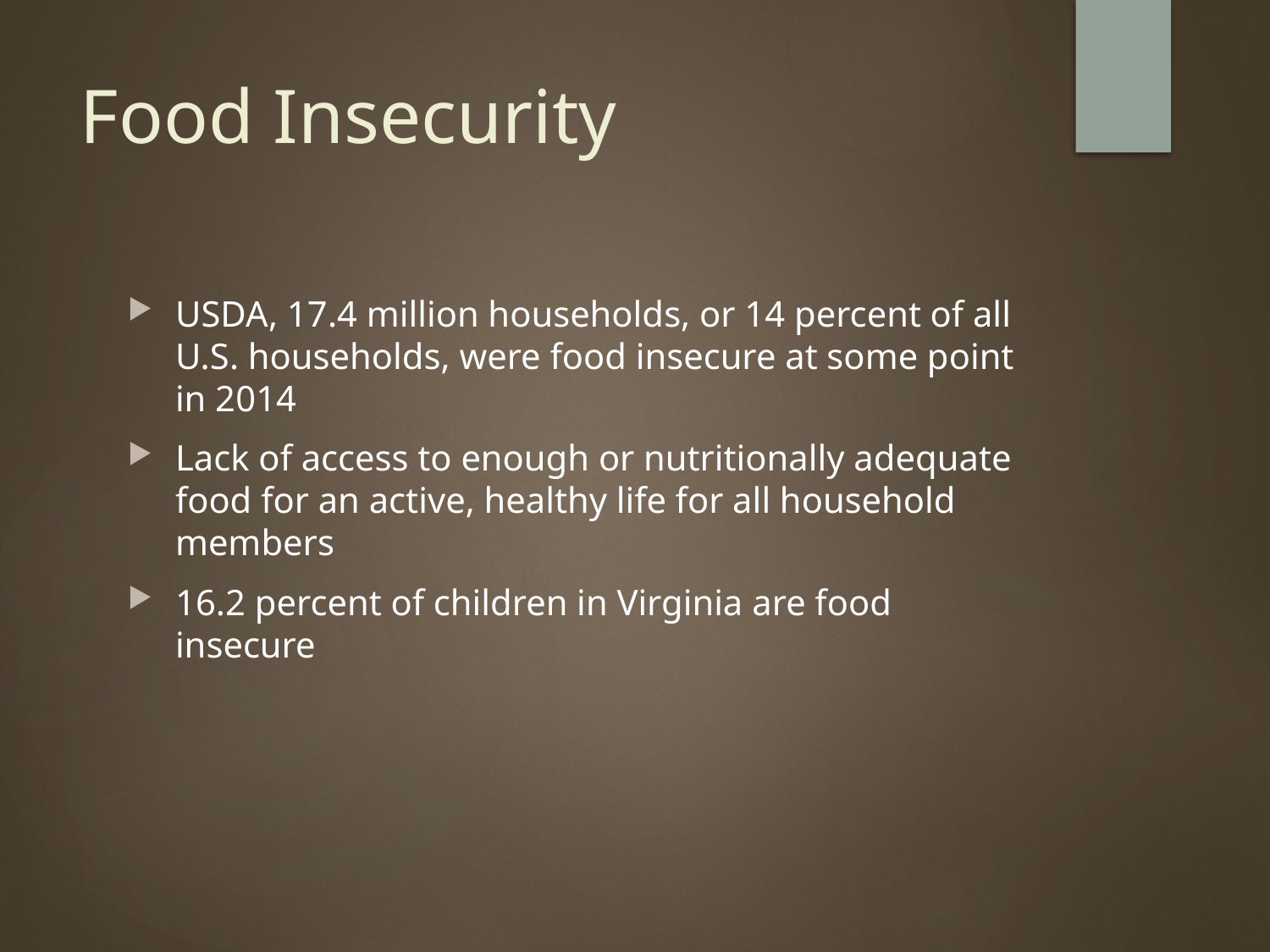

# Food Insecurity
USDA, 17.4 million households, or 14 percent of all U.S. households, were food insecure at some point in 2014
Lack of access to enough or nutritionally adequate food for an active, healthy life for all household members
16.2 percent of children in Virginia are food insecure

## Slide 3
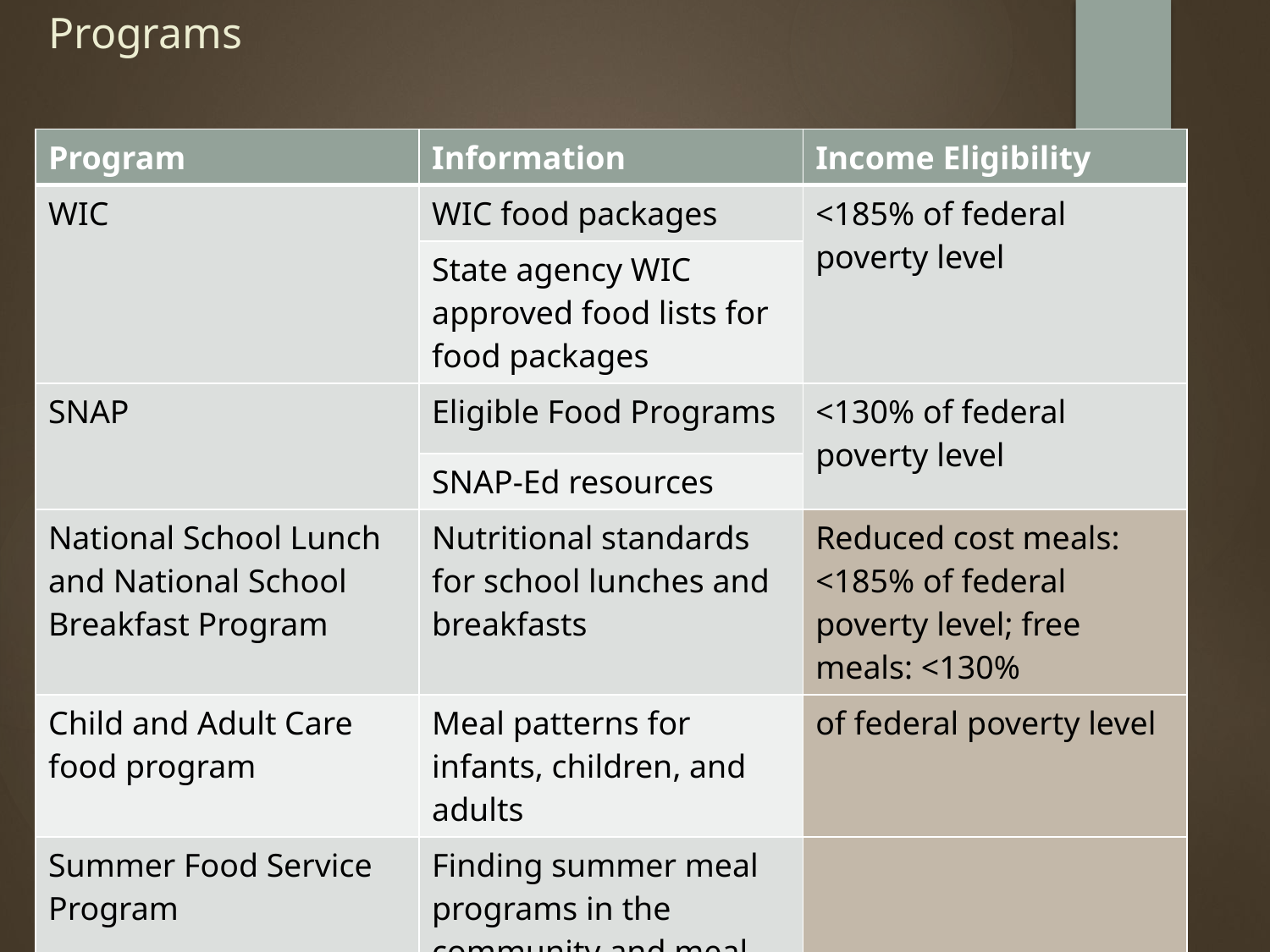

# Programs
| Program | Information | Income Eligibility |
| --- | --- | --- |
| WIC | WIC food packages | <185% of federal poverty level |
| | State agency WIC approved food lists for food packages | |
| SNAP | Eligible Food Programs | <130% of federal poverty level |
| | SNAP-Ed resources | |
| National School Lunch and National School Breakfast Program | Nutritional standards for school lunches and breakfasts | Reduced cost meals: <185% of federal poverty level; free meals: <130% |
| Child and Adult Care food program | Meal patterns for infants, children, and adults | of federal poverty level |
| Summer Food Service Program | Finding summer meal programs in the community and meal content | |

## Slide 4
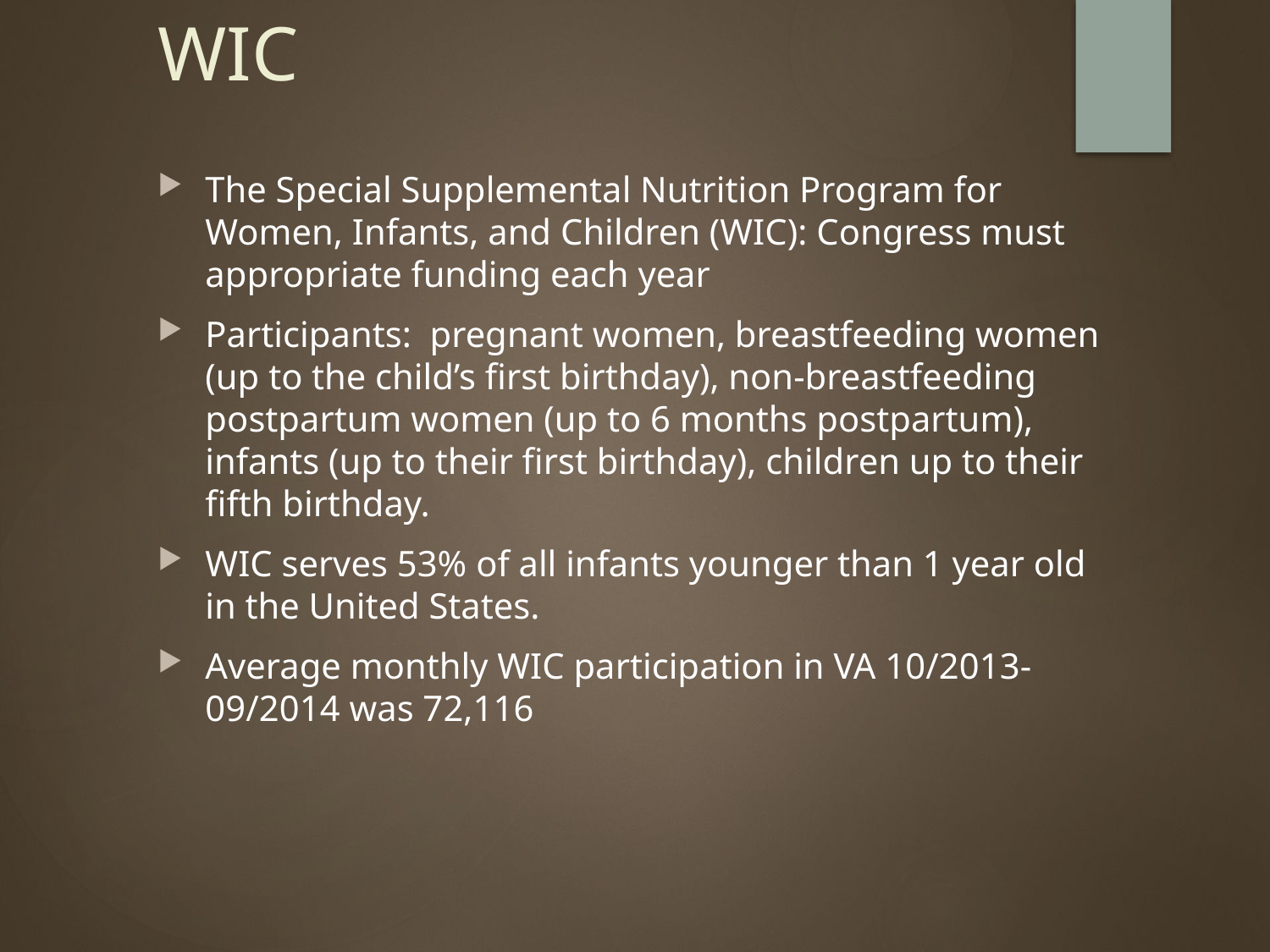

# WIC
The Special Supplemental Nutrition Program for Women, Infants, and Children (WIC): Congress must appropriate funding each year
Participants: pregnant women, breastfeeding women (up to the child’s first birthday), non-breastfeeding postpartum women (up to 6 months postpartum), infants (up to their first birthday), children up to their fifth birthday.
WIC serves 53% of all infants younger than 1 year old in the United States.
Average monthly WIC participation in VA 10/2013-09/2014 was 72,116

## Slide 5
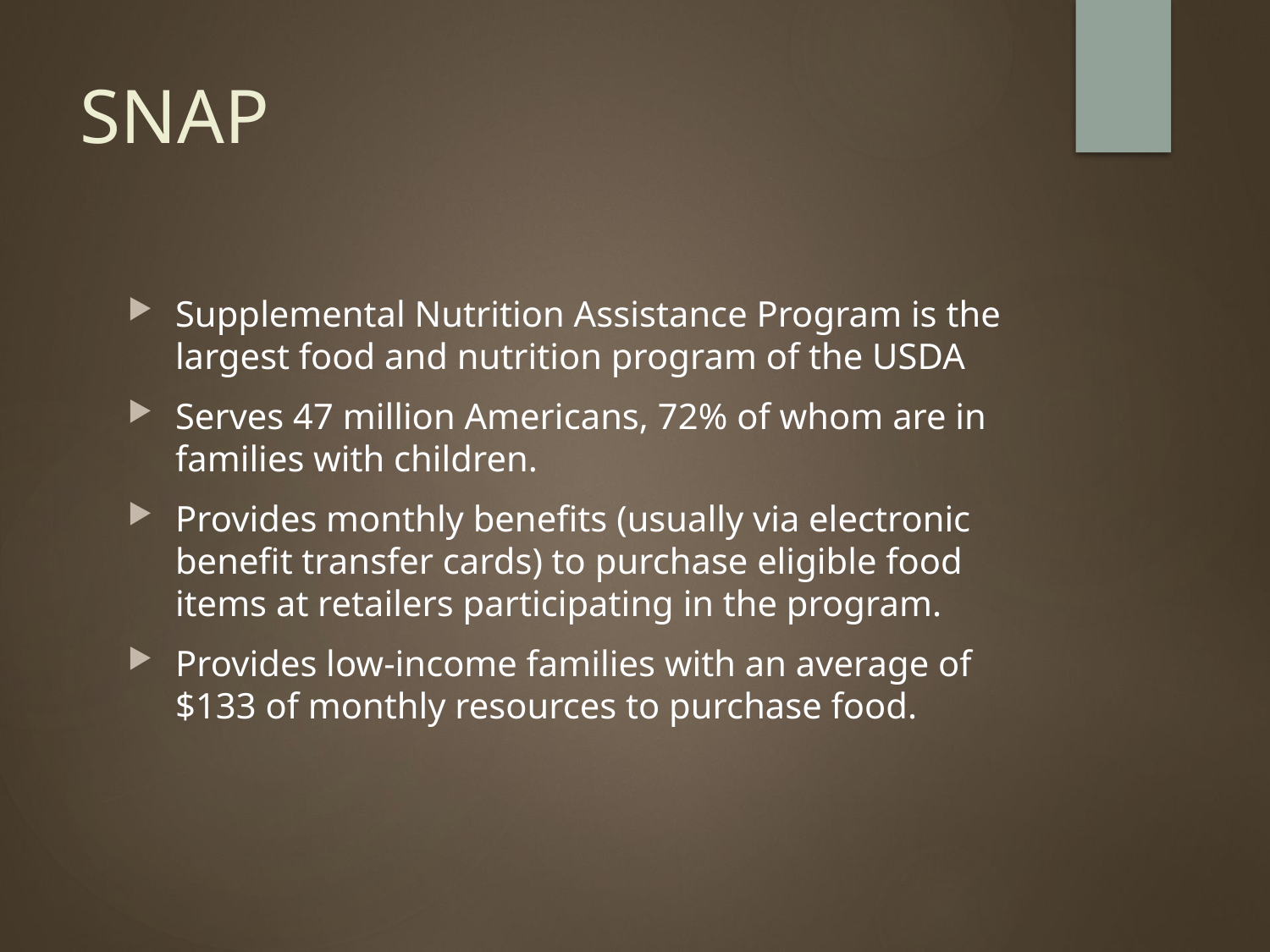

# SNAP
Supplemental Nutrition Assistance Program is the largest food and nutrition program of the USDA
Serves 47 million Americans, 72% of whom are in families with children.
Provides monthly benefits (usually via electronic benefit transfer cards) to purchase eligible food items at retailers participating in the program.
Provides low-income families with an average of $133 of monthly resources to purchase food.

## Slide 6
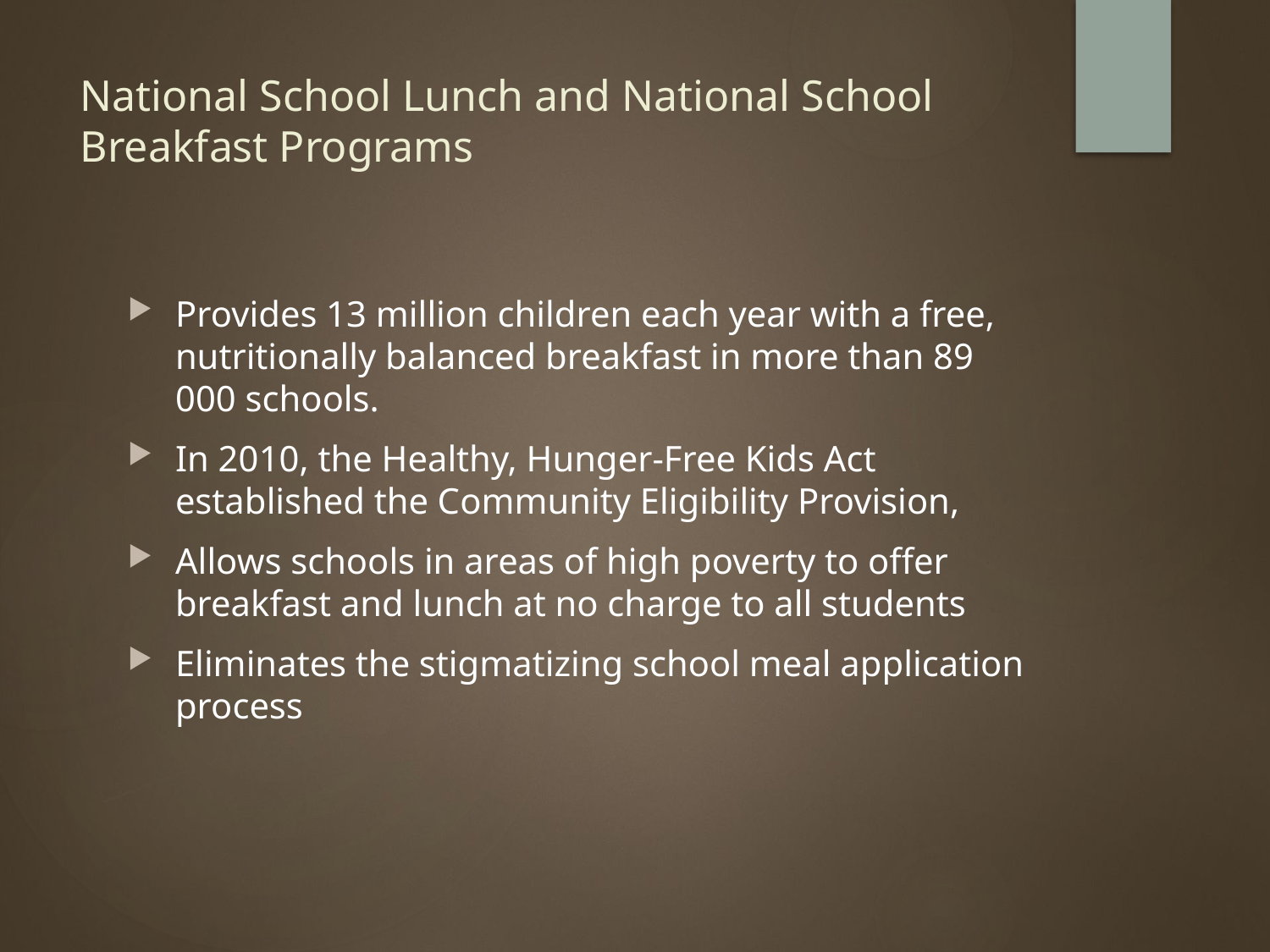

# National School Lunch and National School Breakfast Programs
Provides 13 million children each year with a free, nutritionally balanced breakfast in more than 89 000 schools.
In 2010, the Healthy, Hunger-Free Kids Act established the Community Eligibility Provision,
Allows schools in areas of high poverty to offer breakfast and lunch at no charge to all students
Eliminates the stigmatizing school meal application process

## Slide 7
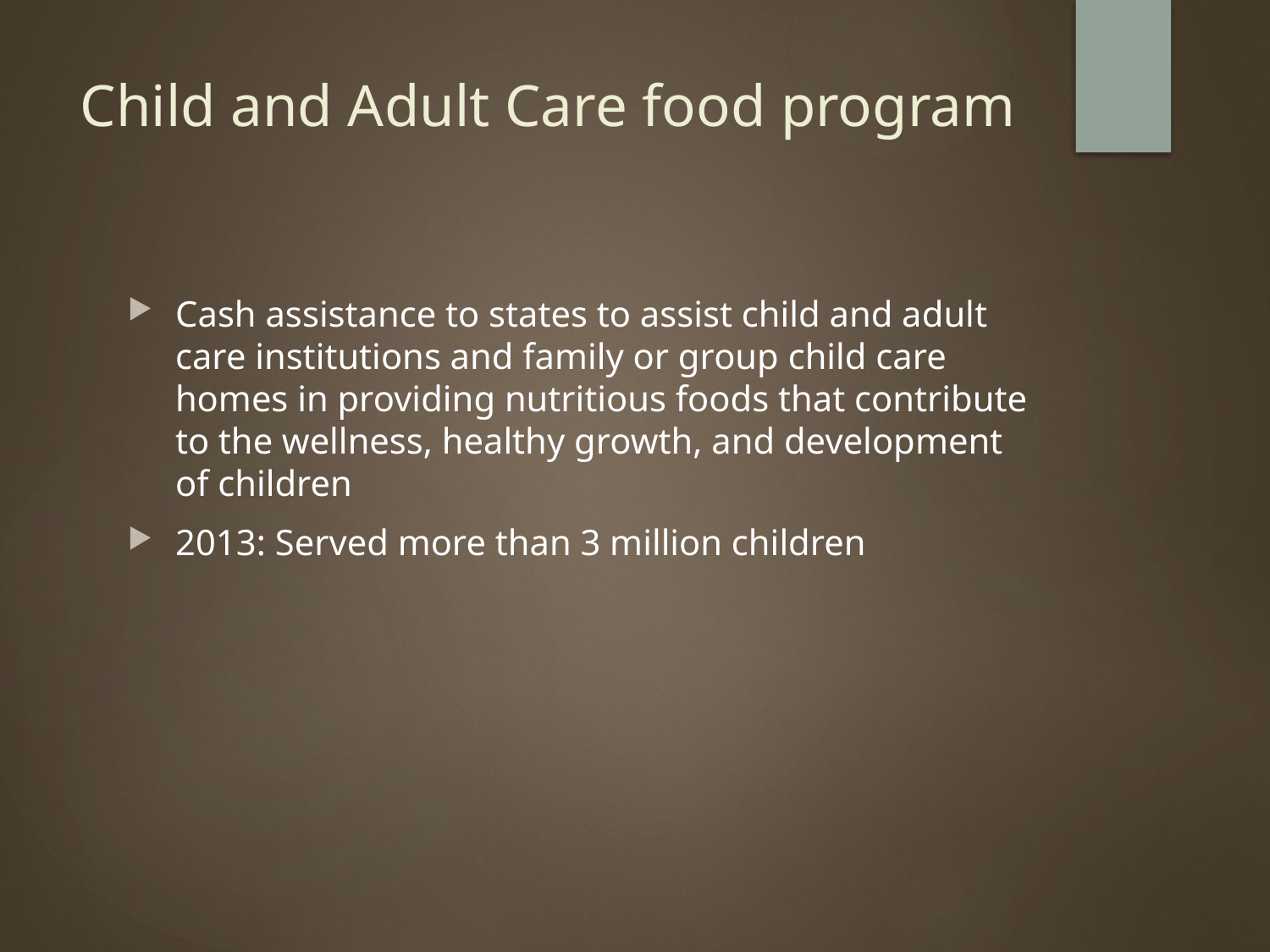

# Child and Adult Care food program
Cash assistance to states to assist child and adult care institutions and family or group child care homes in providing nutritious foods that contribute to the wellness, healthy growth, and development of children
2013: Served more than 3 million children

## Slide 8
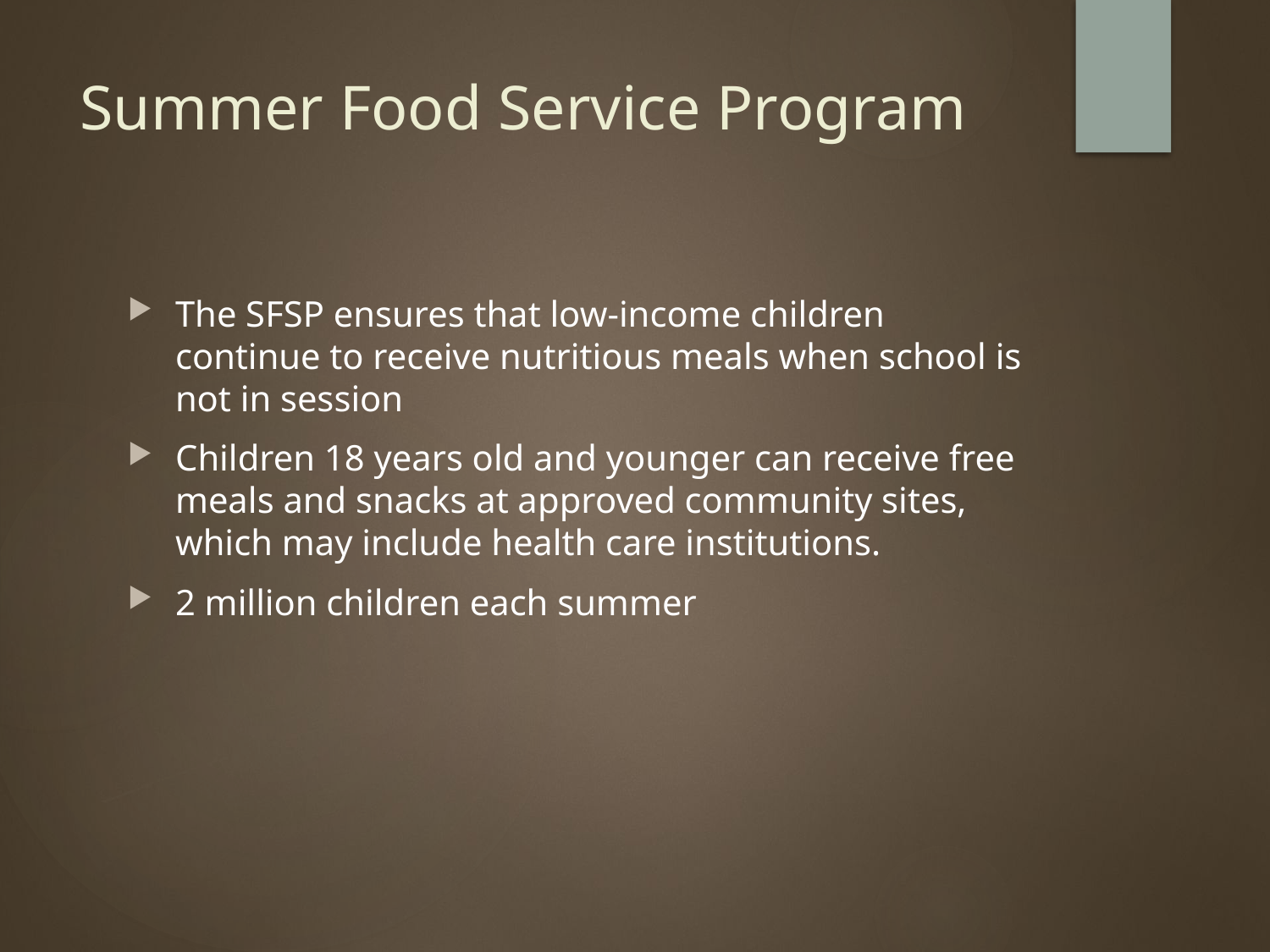

# Summer Food Service Program
The SFSP ensures that low-income children continue to receive nutritious meals when school is not in session
Children 18 years old and younger can receive free meals and snacks at approved community sites, which may include health care institutions.
2 million children each summer

## Slide 9
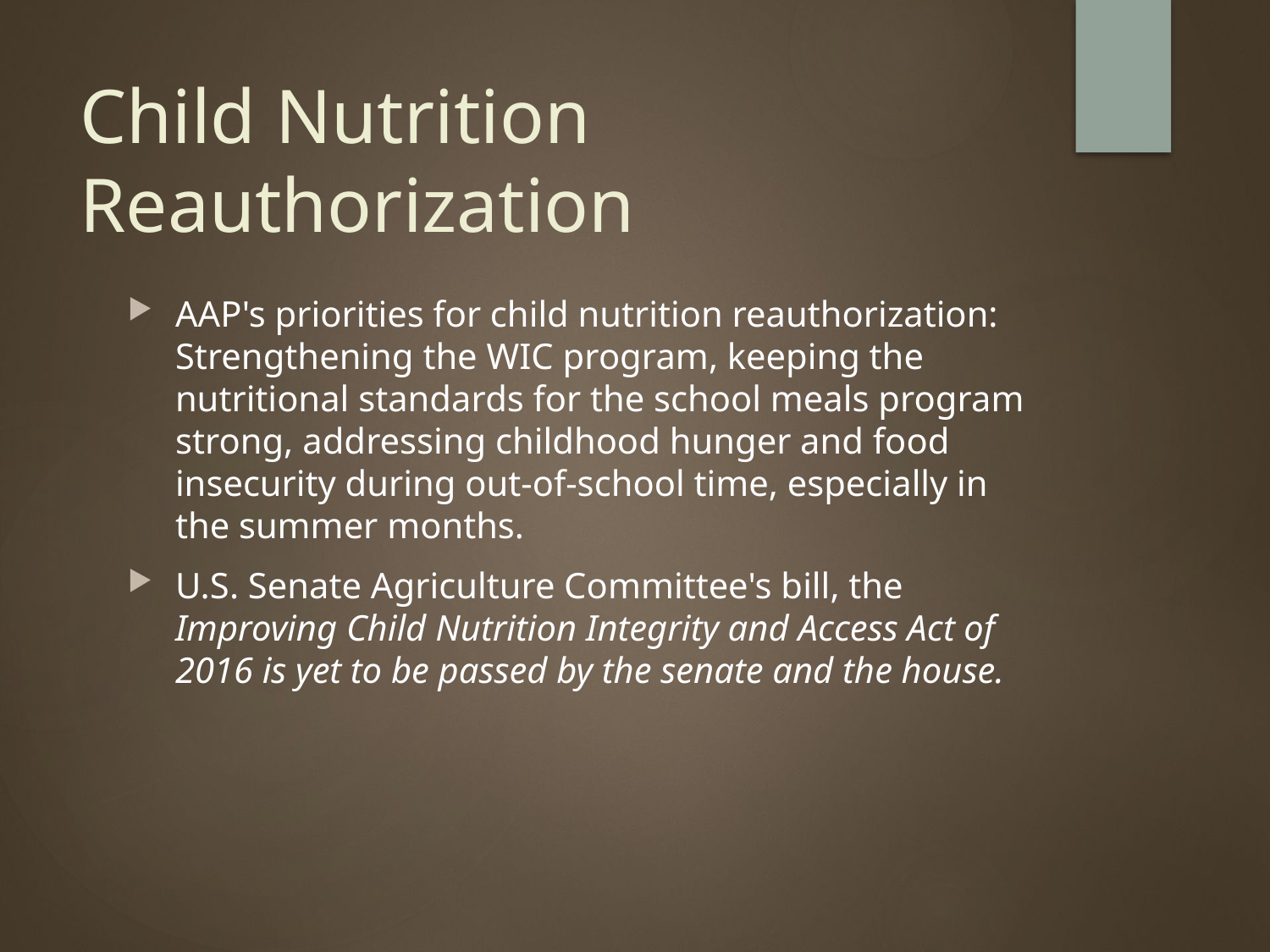

# Child Nutrition Reauthorization
AAP's priorities for child nutrition reauthorization: Strengthening the WIC program, keeping the nutritional standards for the school meals program strong, addressing childhood hunger and food insecurity during out-of-school time, especially in the summer months.
U.S. Senate Agriculture Committee's bill, the Improving Child Nutrition Integrity and Access Act of 2016 is yet to be passed by the senate and the house.

## Slide 10
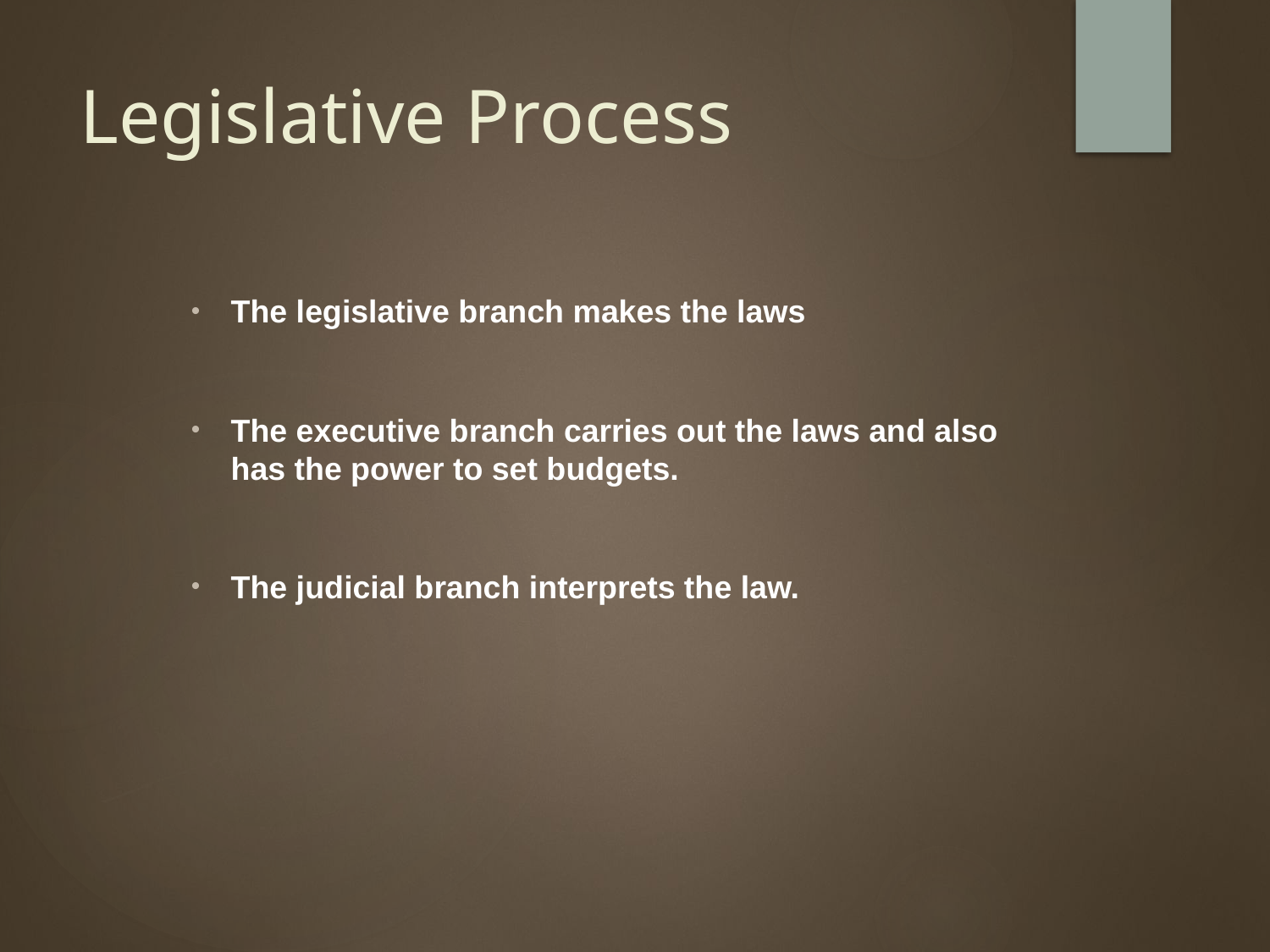

# Legislative Process
The legislative branch makes the laws
The executive branch carries out the laws and also has the power to set budgets.
The judicial branch interprets the law.

## Slide 11
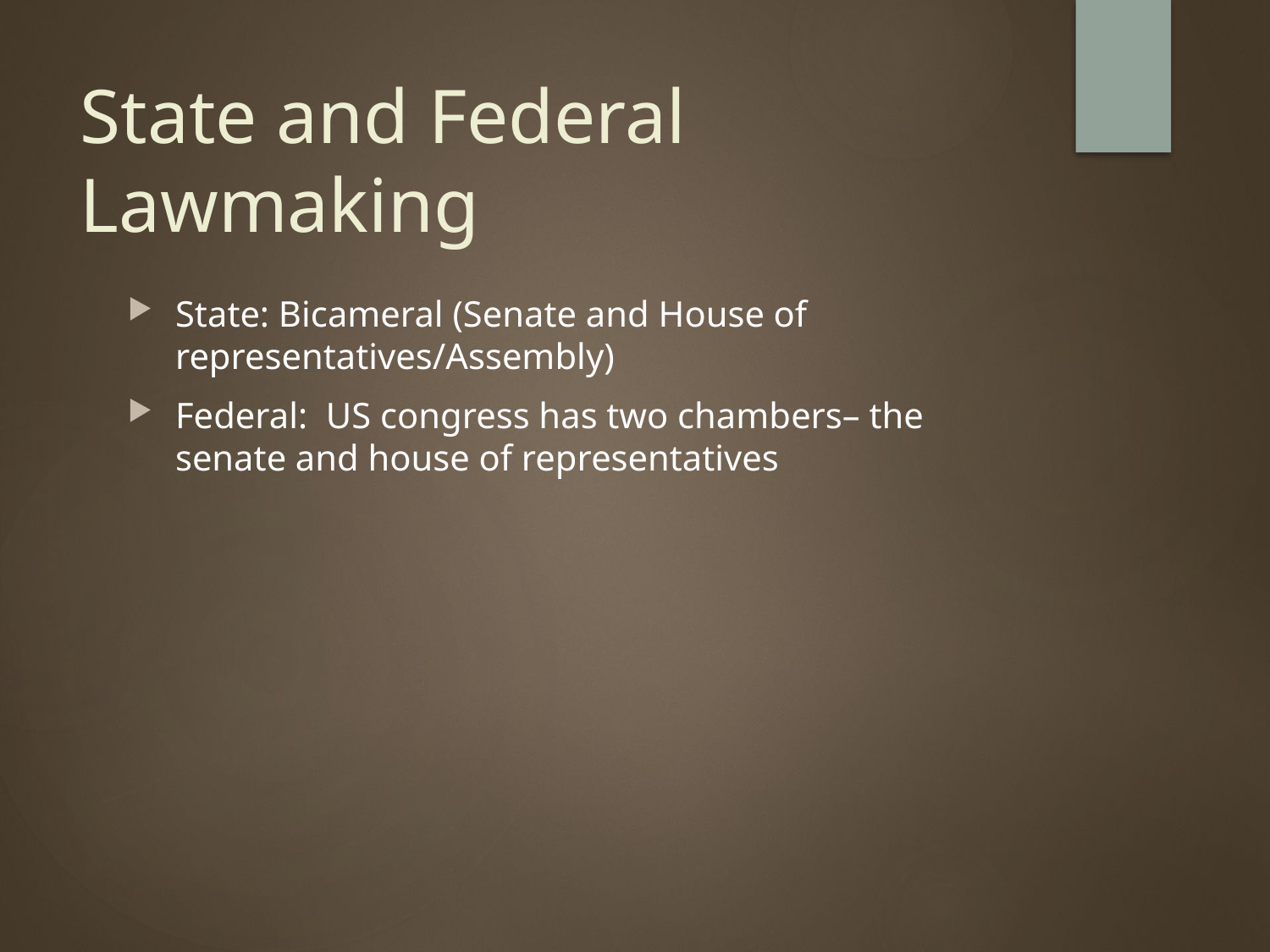

# State and Federal Lawmaking
State: Bicameral (Senate and House of representatives/Assembly)
Federal: US congress has two chambers– the senate and house of representatives

## Slide 12
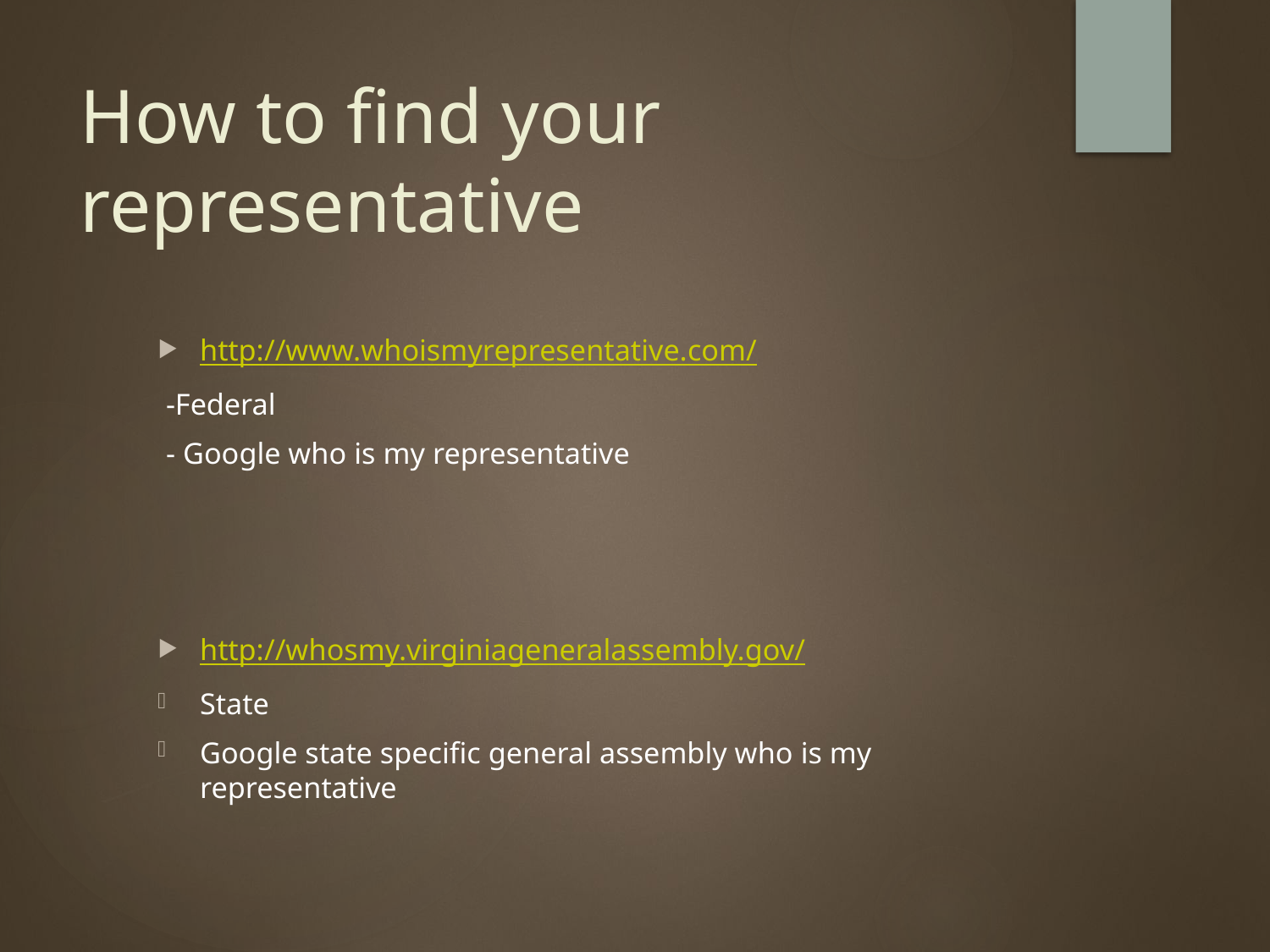

# How to find your representative
http://www.whoismyrepresentative.com/
-Federal
- Google who is my representative
http://whosmy.virginiageneralassembly.gov/
State
Google state specific general assembly who is my representative

## Slide 13
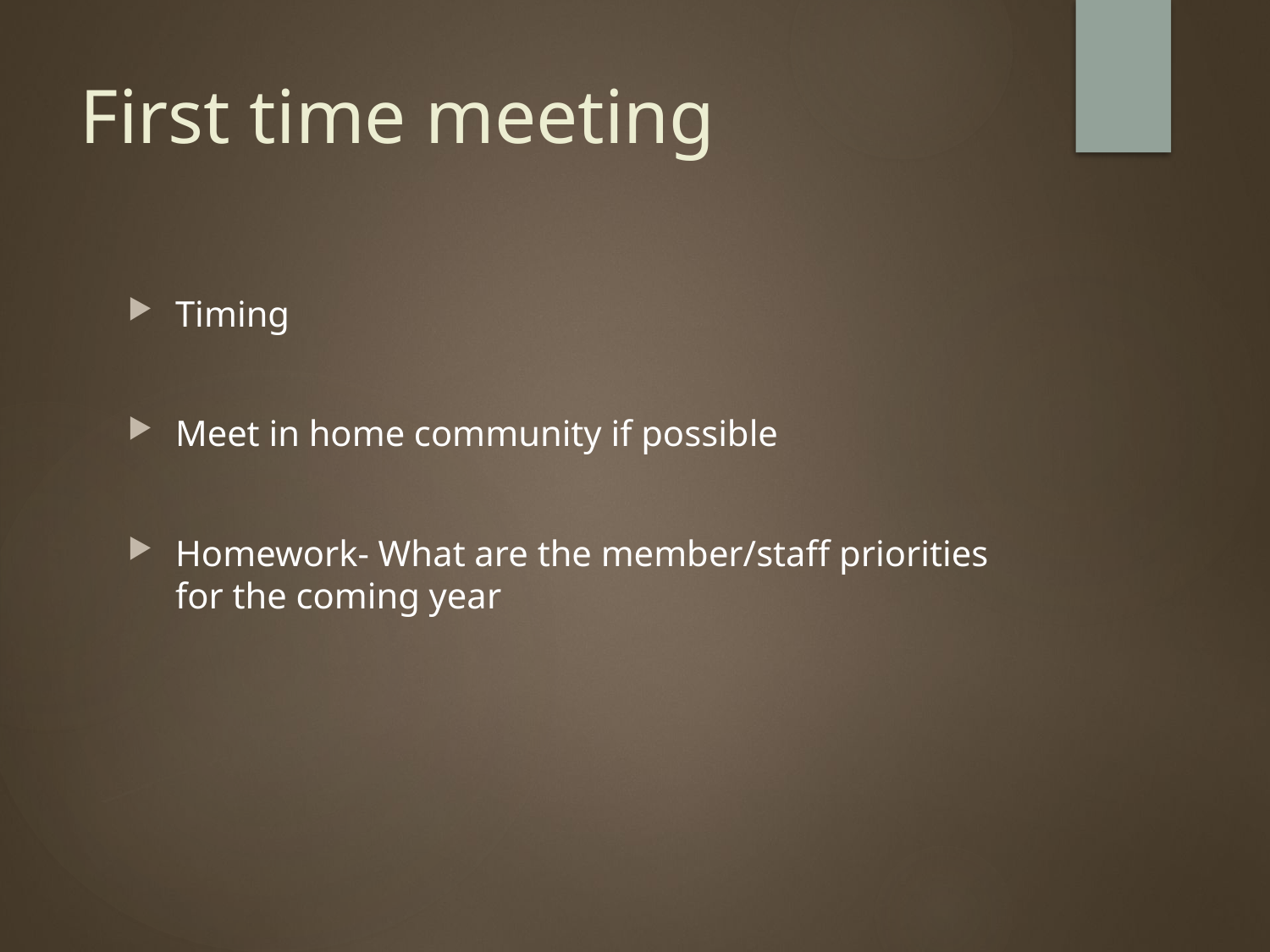

# First time meeting
Timing
Meet in home community if possible
Homework- What are the member/staff priorities for the coming year

## Slide 14
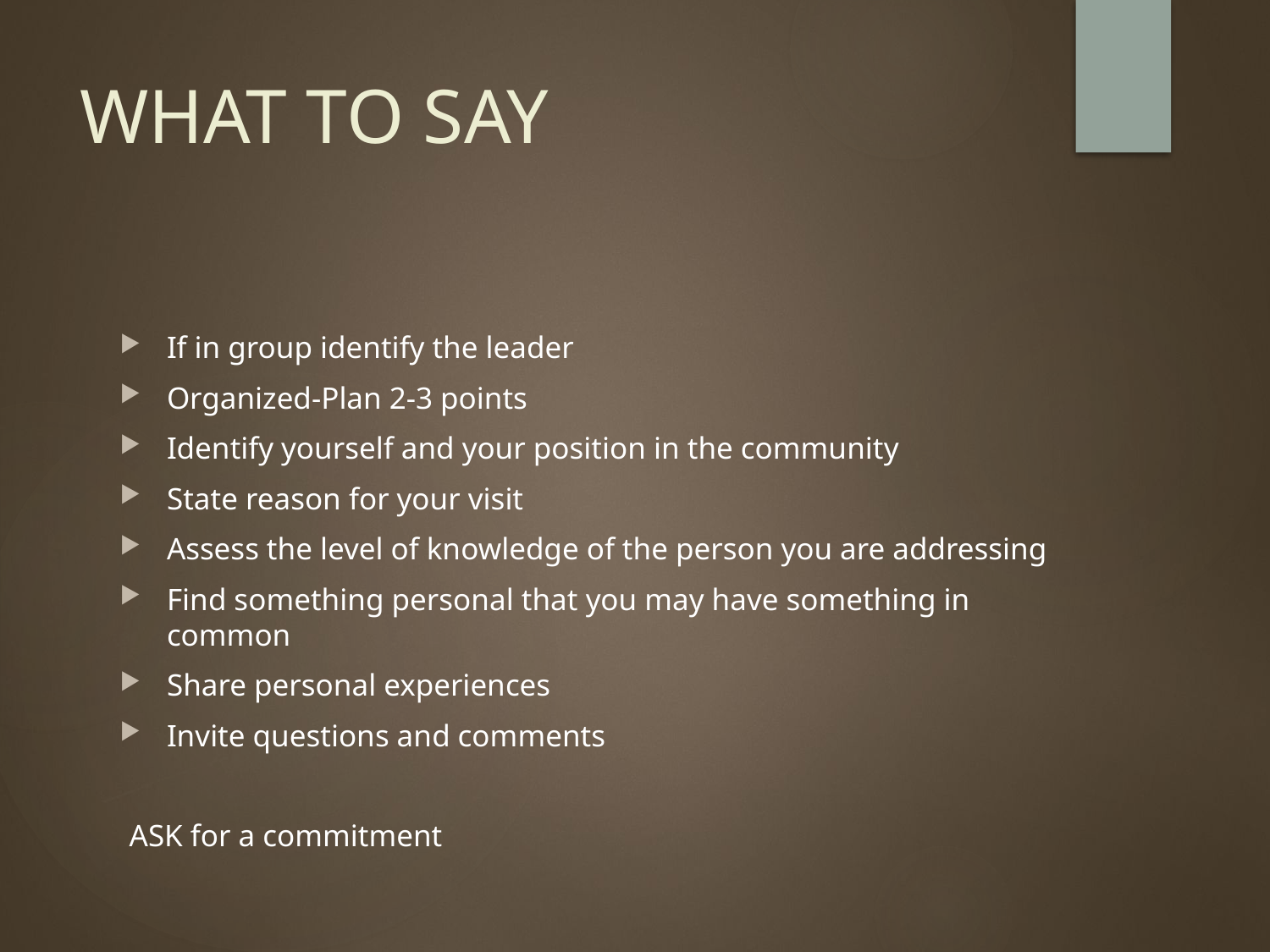

# WHAT TO SAY
If in group identify the leader
Organized-Plan 2-3 points
Identify yourself and your position in the community
State reason for your visit
Assess the level of knowledge of the person you are addressing
Find something personal that you may have something in common
Share personal experiences
Invite questions and comments
ASK for a commitment

## Slide 15
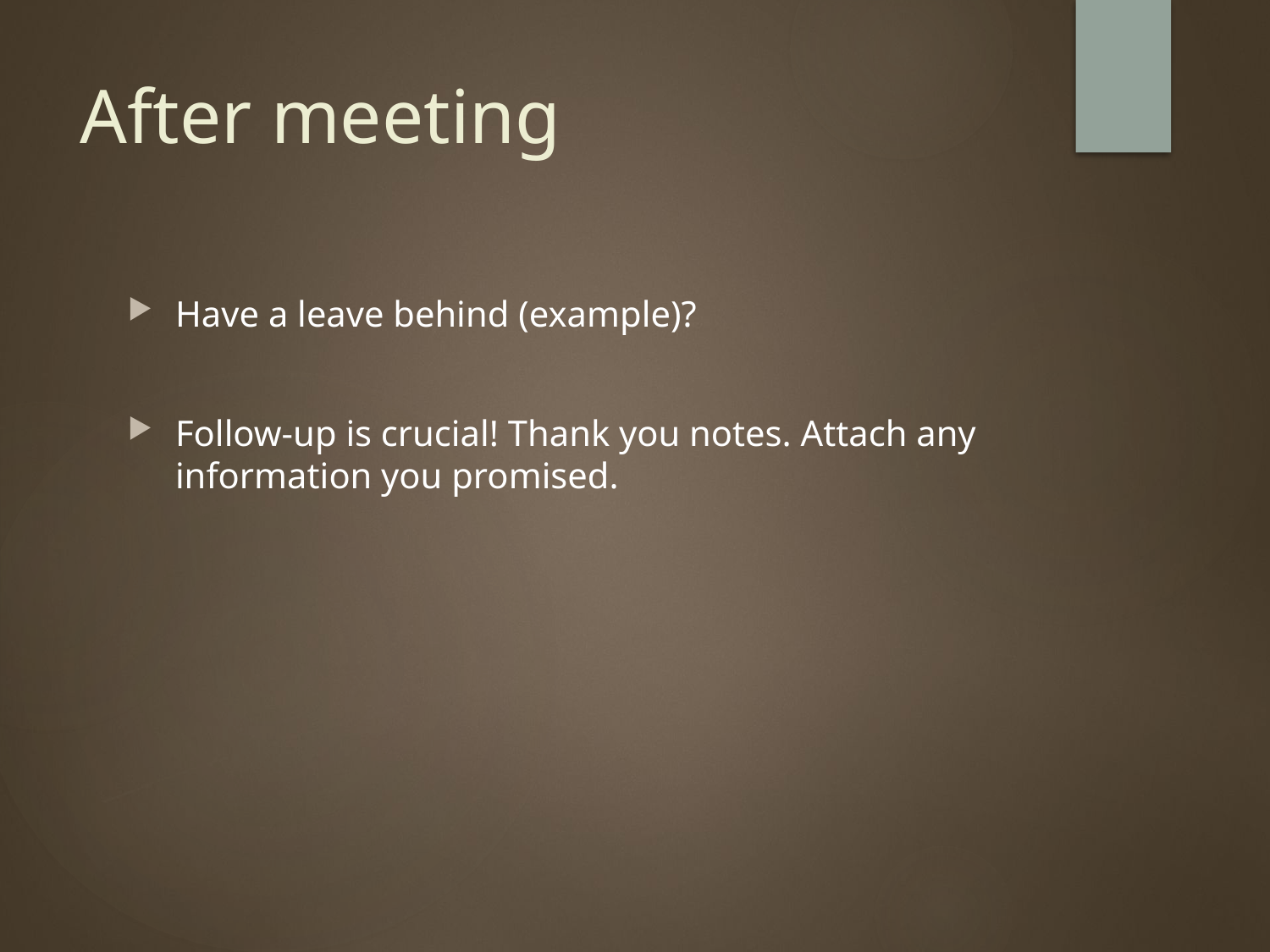

# After meeting
Have a leave behind (example)?
Follow-up is crucial! Thank you notes. Attach any information you promised.

## Slide 16
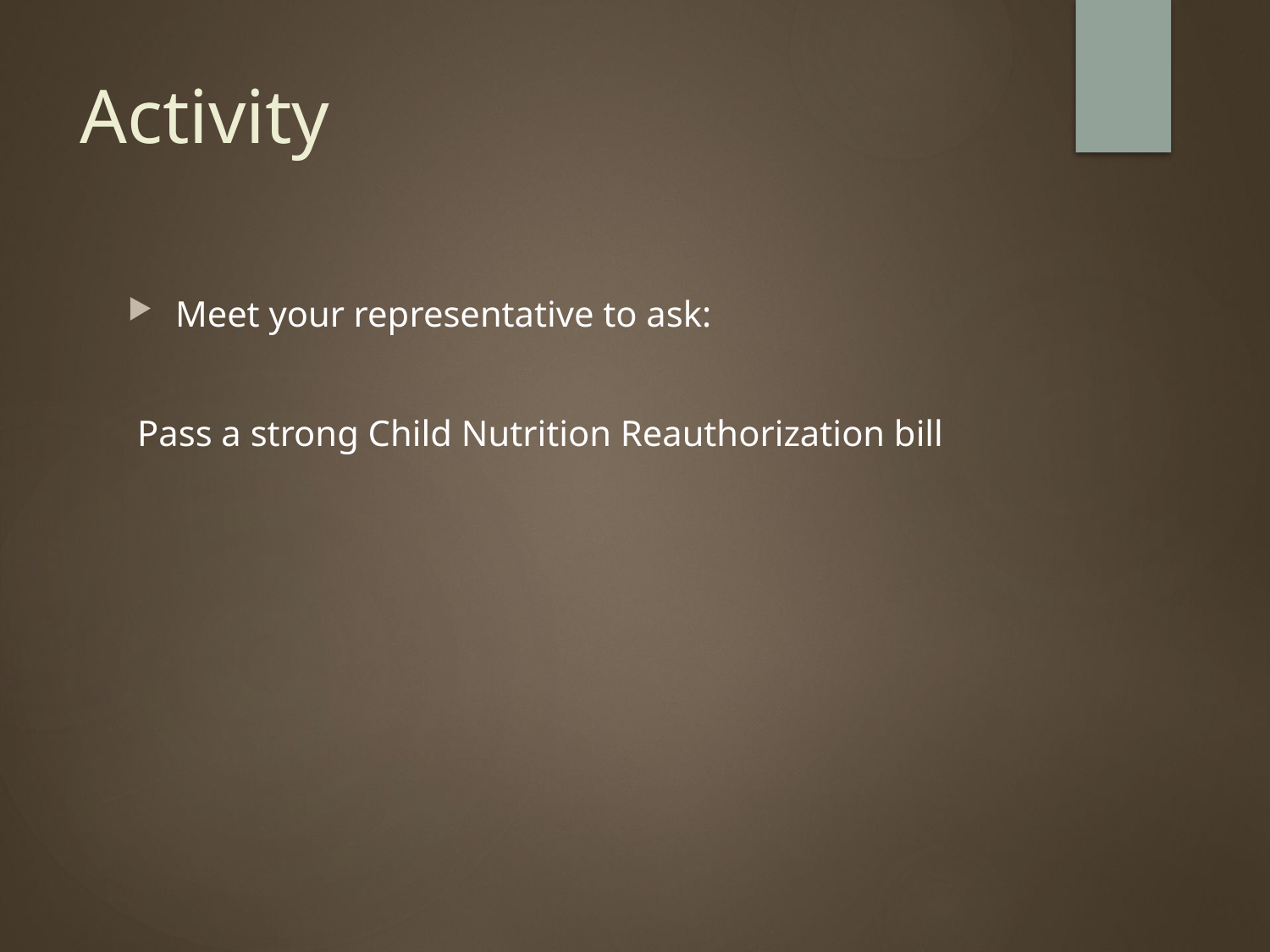

# Activity
Meet your representative to ask:
Pass a strong Child Nutrition Reauthorization bill
