# Supplementary material for: Advancing Advocacy: Implementation of a Child Health Advocacy Curriculum in a Pediatrics Residency Program
Source: MedEdPORTAL. 2020 Feb 14;16:10882. doi: 10.15766/mep_2374-8265.10882 (PMC7062538; doi:10.15766/mep_2374-8265.10882)
Supplement: Supplementary file 1 — A. Lecture 1.pptx B. Lecture 2.pptx C. Lecture 3.ppt D. Lecture 4.pptx E. Workshop 1.pptx F. Workshop 1 Skill Checklist.pdf G. Workshop 2.pptx H. Workshop 3.pptx I. Curriculum Survey.docx [file mep-16-10882-s001.zip › F. Workshop 1 Skill Checklist.pdf]

Meeting Your Representative
First Time Encounter Checklist

☐ Do your research before the 1st meeting.
Notes:

☐ What are the member/staff priorities for the coming year?
Notes:

☐ What committees does the Representative / Senator sit on?
Notes:

☐ Decide what each of you will say.
Notes:

☐ Identify yourself and your position in the community.
Notes:

☐ State the reason for your visit.
Notes:

☐ Assess the level of knowledge of the person you are addressing.
Notes:

☐ Share personal experiences.
Notes:

☐ Invite questions and comments.
Notes:

☐ Ask for a commitment.
Notes:

☐ Leave behind a business card.
Notes:

☐ Thank you and follow-up.
Notes:
