# Supplementary material for: Advancing Advocacy: Implementation of a Child Health Advocacy Curriculum in a Pediatrics Residency Program
Source: MedEdPORTAL. 2020 Feb 14;16:10882. doi: 10.15766/mep_2374-8265.10882 (PMC7062538; doi:10.15766/mep_2374-8265.10882)
Supplement: Supplementary file 1 — A. Lecture 1.pptx B. Lecture 2.pptx C. Lecture 3.ppt D. Lecture 4.pptx E. Workshop 1.pptx F. Workshop 1 Skill Checklist.pdf G. Workshop 2.pptx H. Workshop 3.pptx I. Curriculum Survey.docx [file mep-16-10882-s001.zip › G. Workshop 2.pptx]

## Slide 1
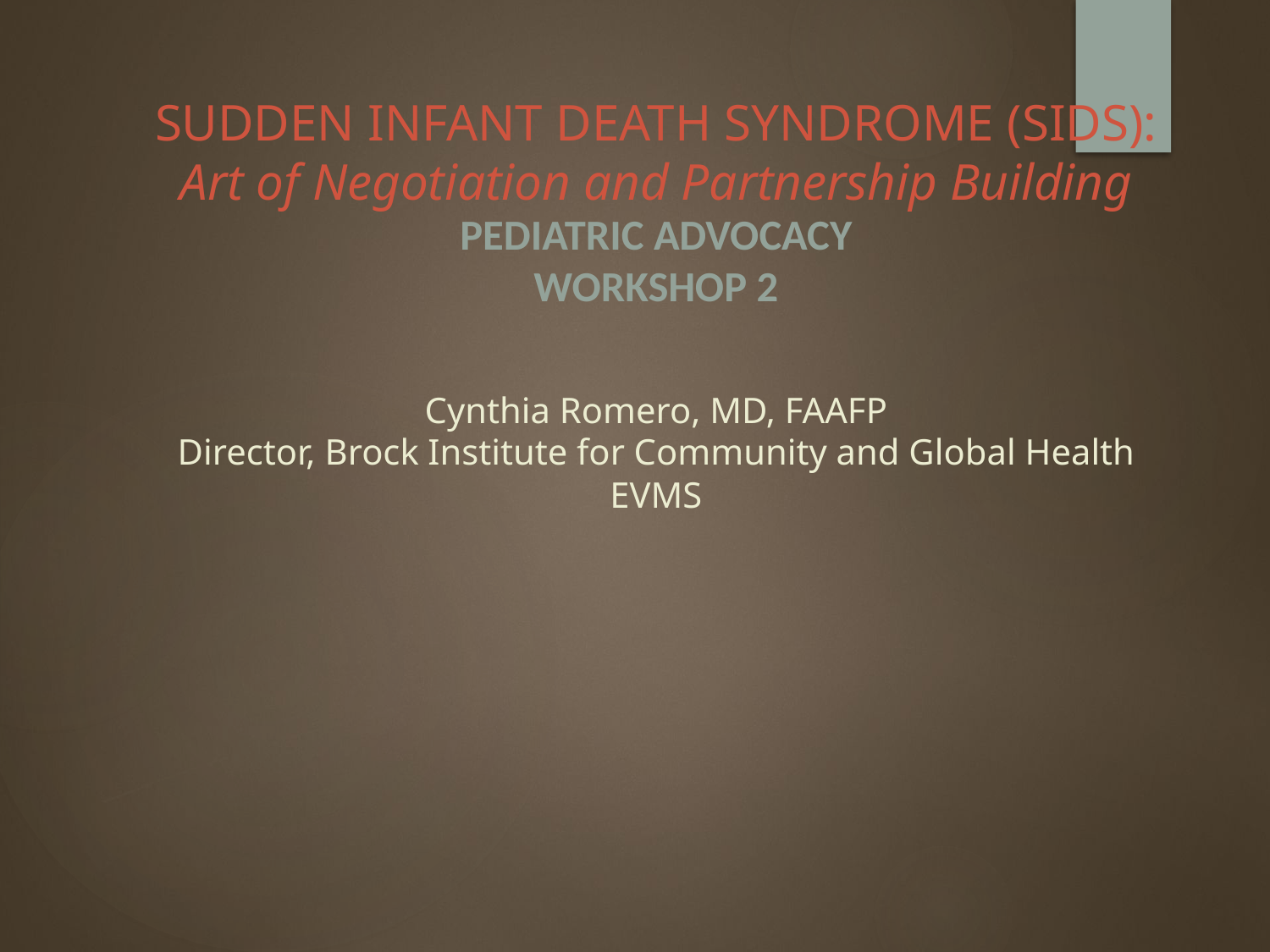

Pediatric Advocacy
Workshop 2
# SUDDEN INFANT DEATH SYNDROME (SIDS): Art of Negotiation and Partnership Building Cynthia Romero, MD, FAAFP Director, Brock Institute for Community and Global Health EVMS

## Slide 2
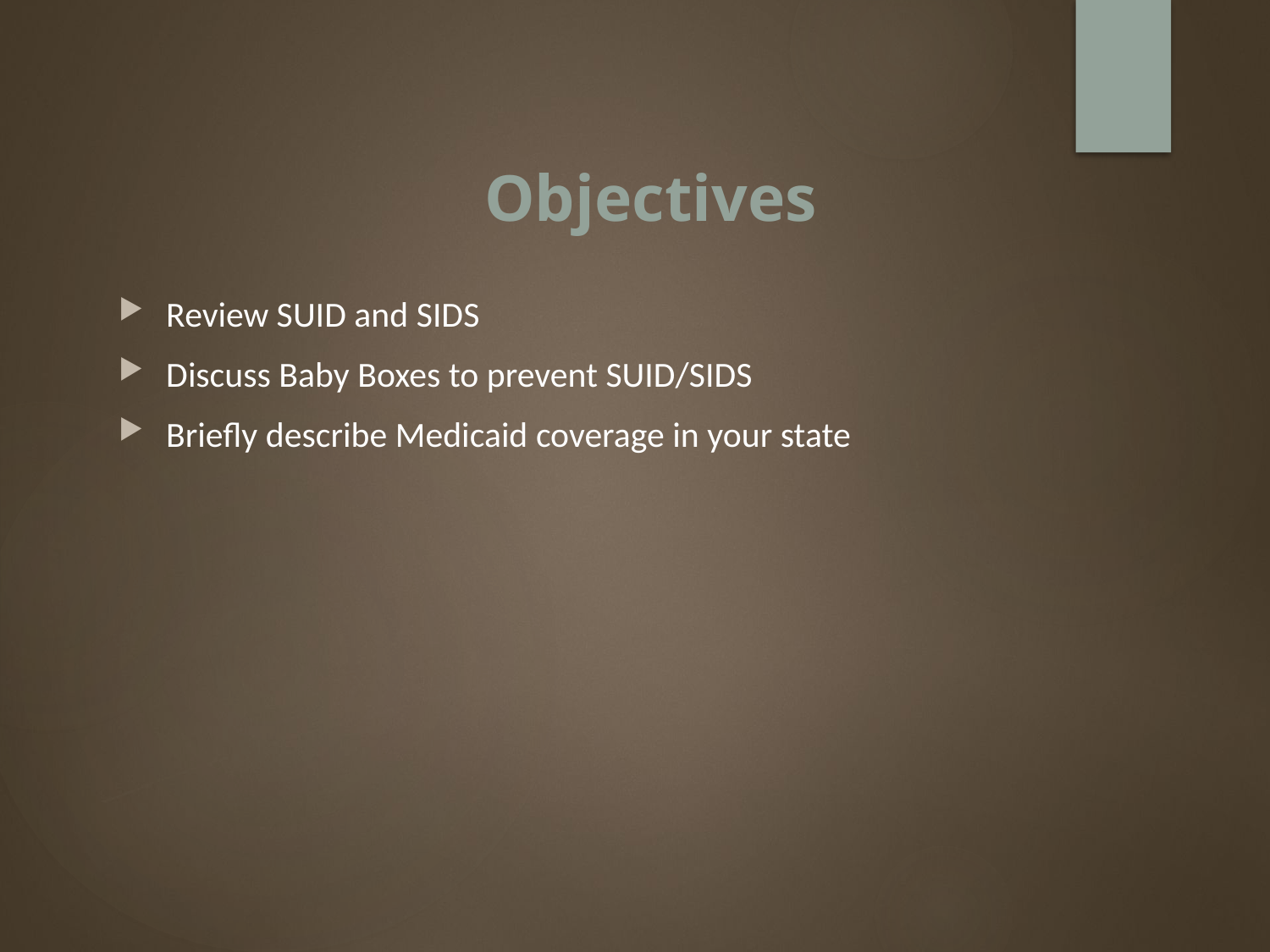

Objectives
Review SUID and SIDS
Discuss Baby Boxes to prevent SUID/SIDS
Briefly describe Medicaid coverage in your state

## Slide 3
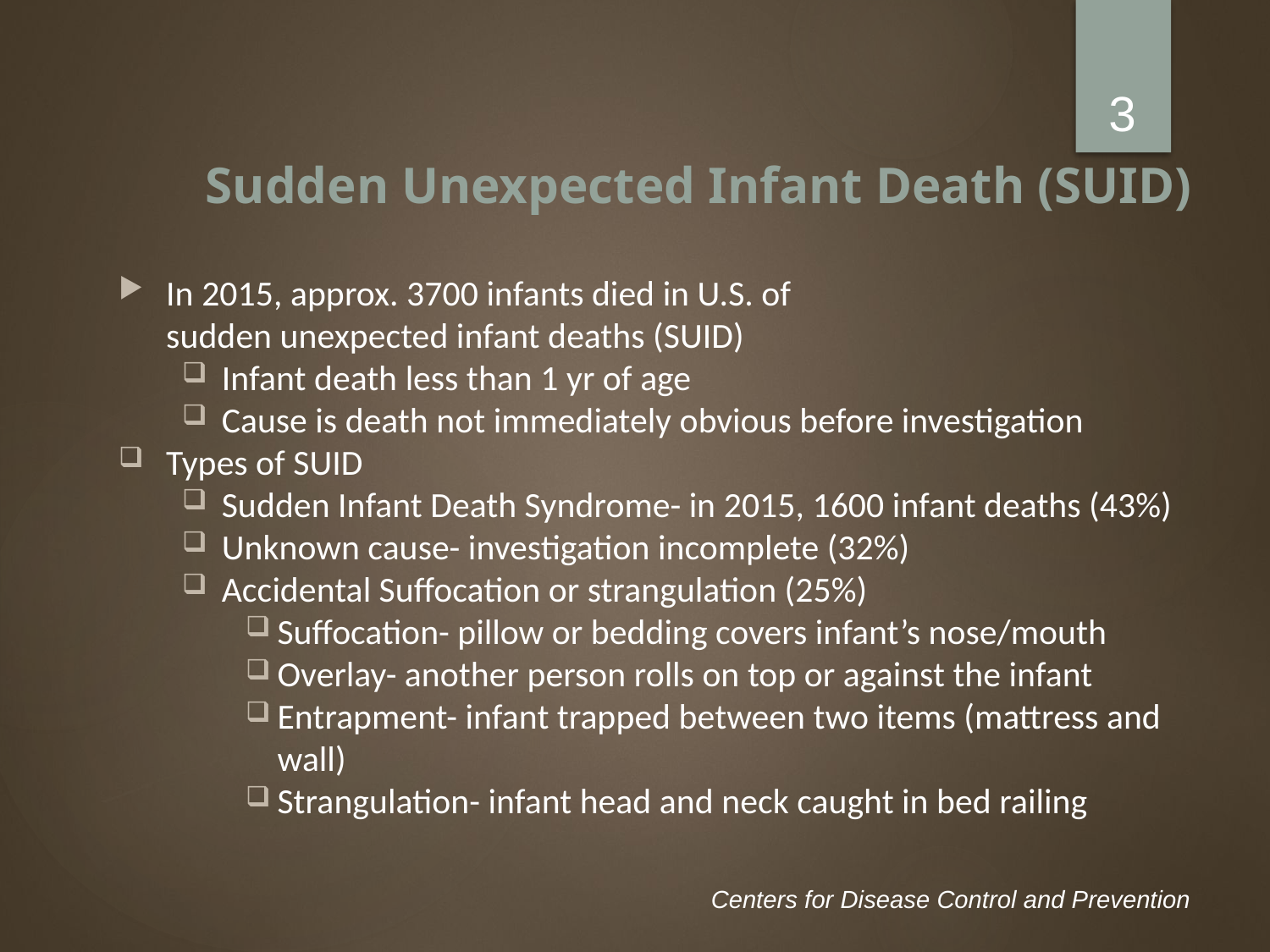

3
Sudden Unexpected Infant Death (SUID)
In 2015, approx. 3700 infants died in U.S. of
	sudden unexpected infant deaths (SUID)
Infant death less than 1 yr of age
Cause is death not immediately obvious before investigation
Types of SUID
Sudden Infant Death Syndrome- in 2015, 1600 infant deaths (43%)
Unknown cause- investigation incomplete (32%)
Accidental Suffocation or strangulation (25%)
Suffocation- pillow or bedding covers infant’s nose/mouth
Overlay- another person rolls on top or against the infant
Entrapment- infant trapped between two items (mattress and wall)
Strangulation- infant head and neck caught in bed railing
Centers for Disease Control and Prevention

## Slide 4
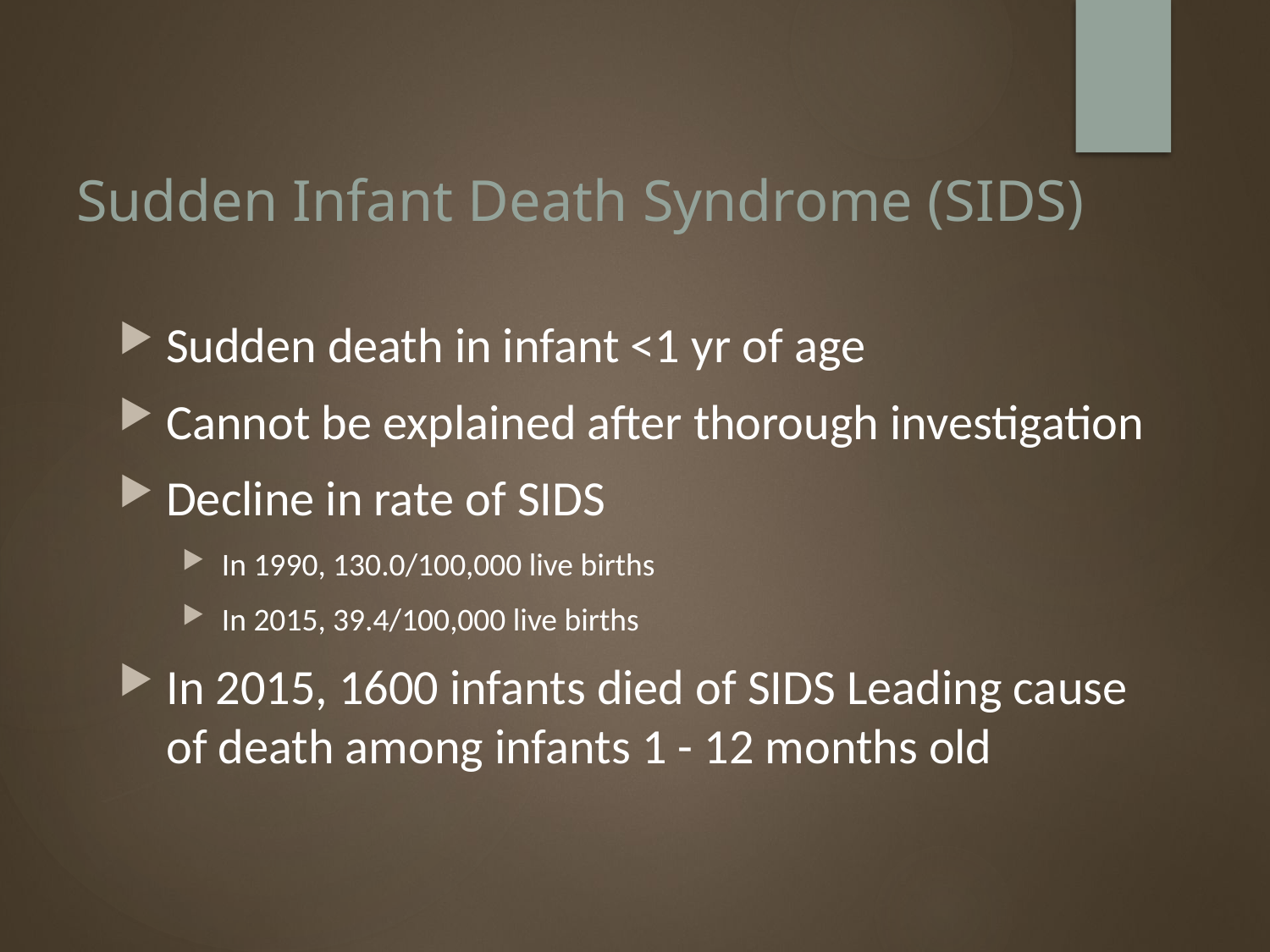

# Sudden Infant Death Syndrome (SIDS)
Sudden death in infant <1 yr of age
Cannot be explained after thorough investigation
Decline in rate of SIDS
In 1990, 130.0/100,000 live births
In 2015, 39.4/100,000 live births
In 2015, 1600 infants died of SIDS Leading cause of death among infants 1 - 12 months old

## Slide 5
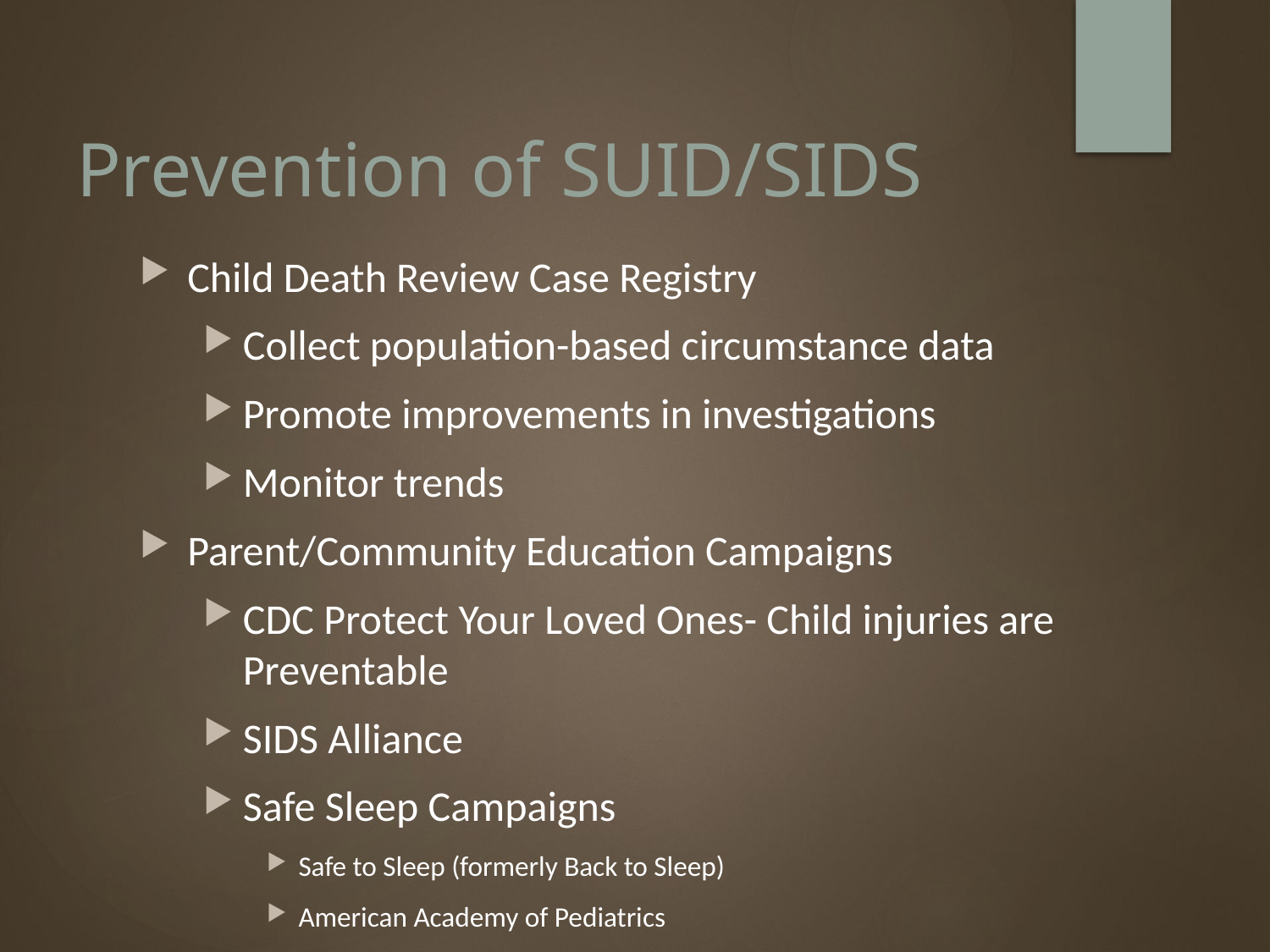

# Prevention of SUID/SIDS
Child Death Review Case Registry
Collect population-based circumstance data
Promote improvements in investigations
Monitor trends
Parent/Community Education Campaigns
CDC Protect Your Loved Ones- Child injuries are Preventable
SIDS Alliance
Safe Sleep Campaigns
Safe to Sleep (formerly Back to Sleep)
American Academy of Pediatrics

## Slide 6
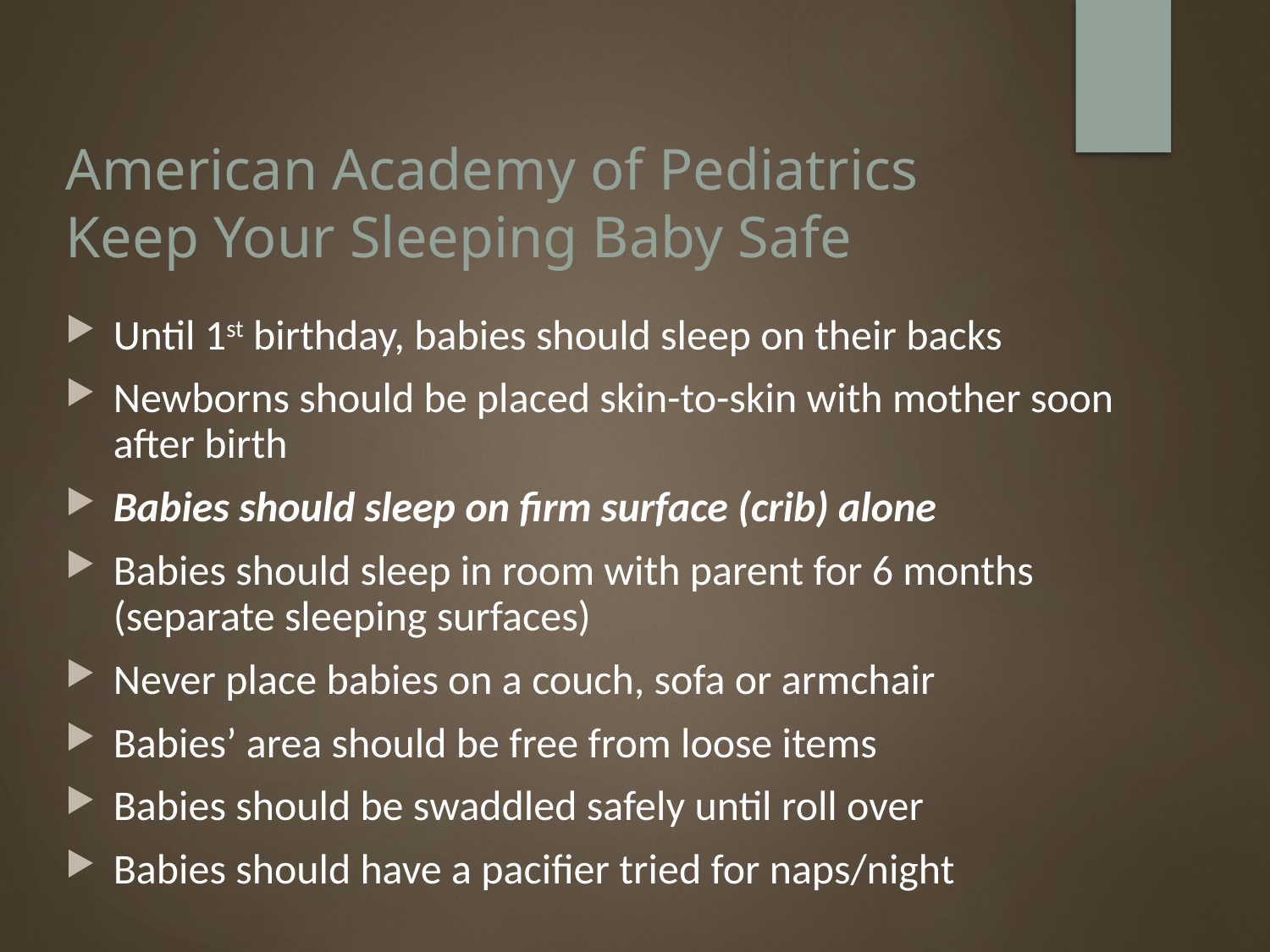

# American Academy of Pediatrics Keep Your Sleeping Baby Safe
Until 1st birthday, babies should sleep on their backs
Newborns should be placed skin-to-skin with mother soon after birth
Babies should sleep on firm surface (crib) alone
Babies should sleep in room with parent for 6 months (separate sleeping surfaces)
Never place babies on a couch, sofa or armchair
Babies’ area should be free from loose items
Babies should be swaddled safely until roll over
Babies should have a pacifier tried for naps/night

## Slide 7
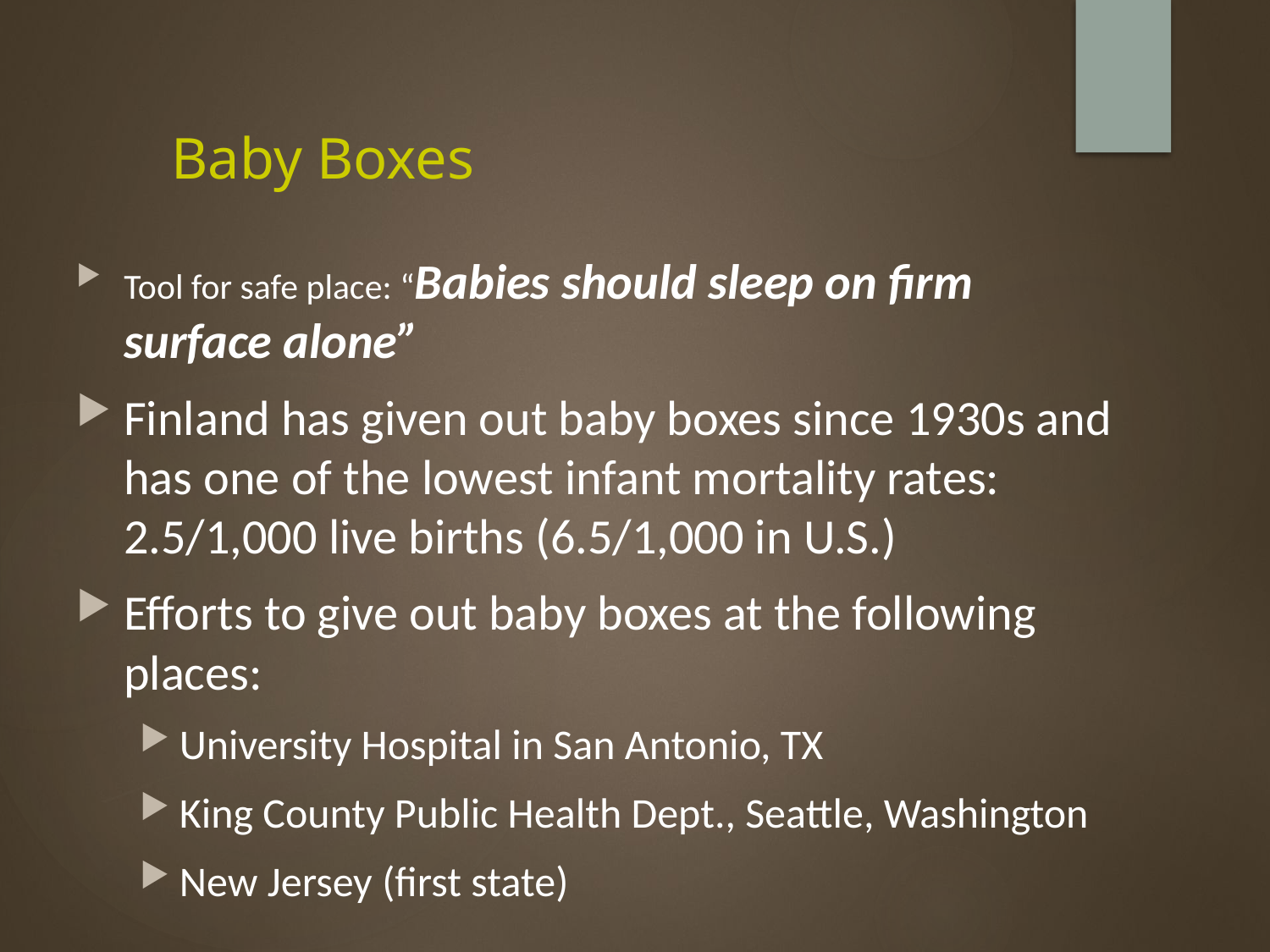

# Baby Boxes
Tool for safe place: “Babies should sleep on firm surface alone”
Finland has given out baby boxes since 1930s and has one of the lowest infant mortality rates: 2.5/1,000 live births (6.5/1,000 in U.S.)
Efforts to give out baby boxes at the following places:
University Hospital in San Antonio, TX
King County Public Health Dept., Seattle, Washington
New Jersey (first state)

## Slide 8
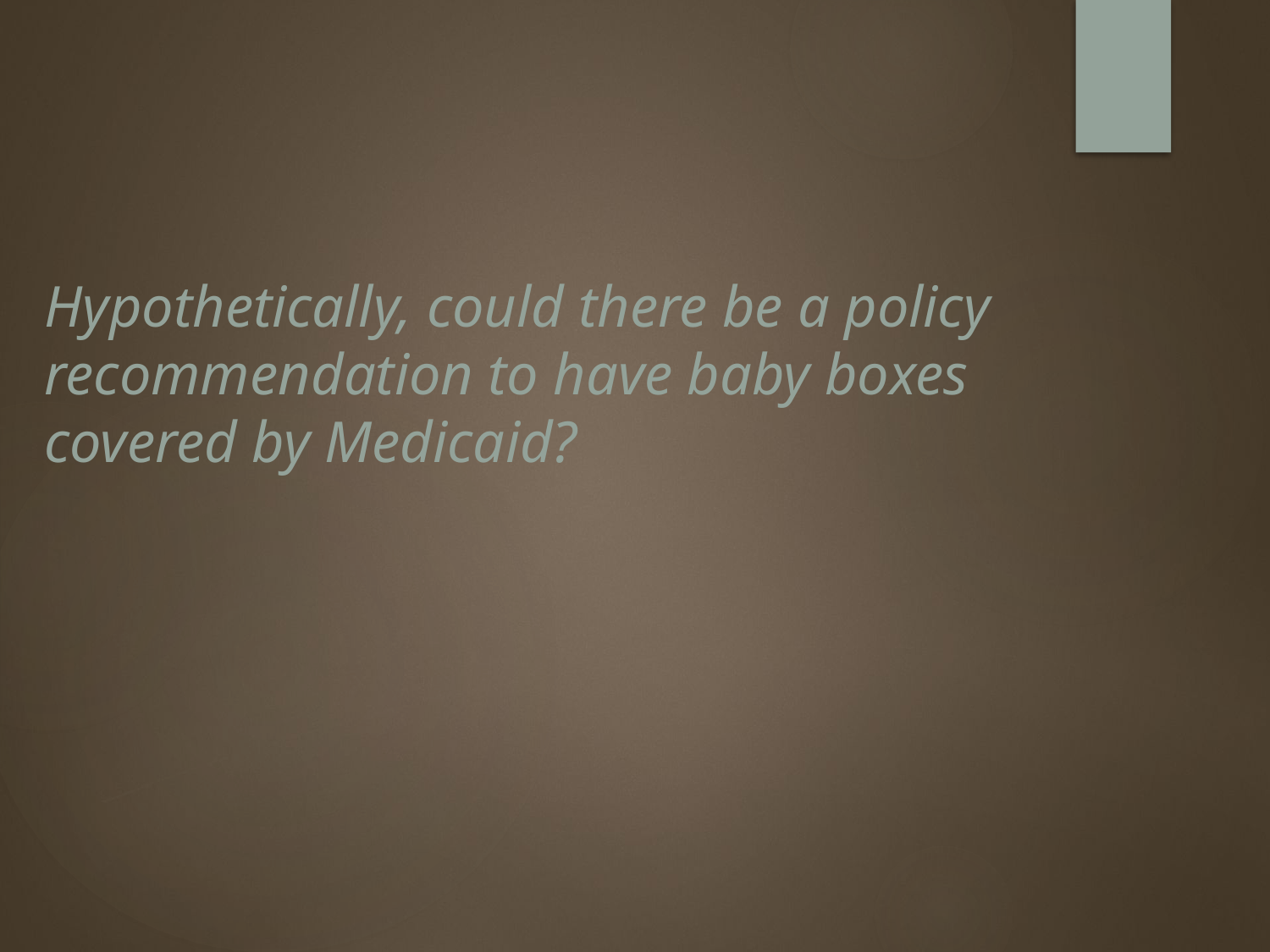

# Hypothetically, could there be a policy recommendation to have baby boxes covered by Medicaid?

## Slide 9
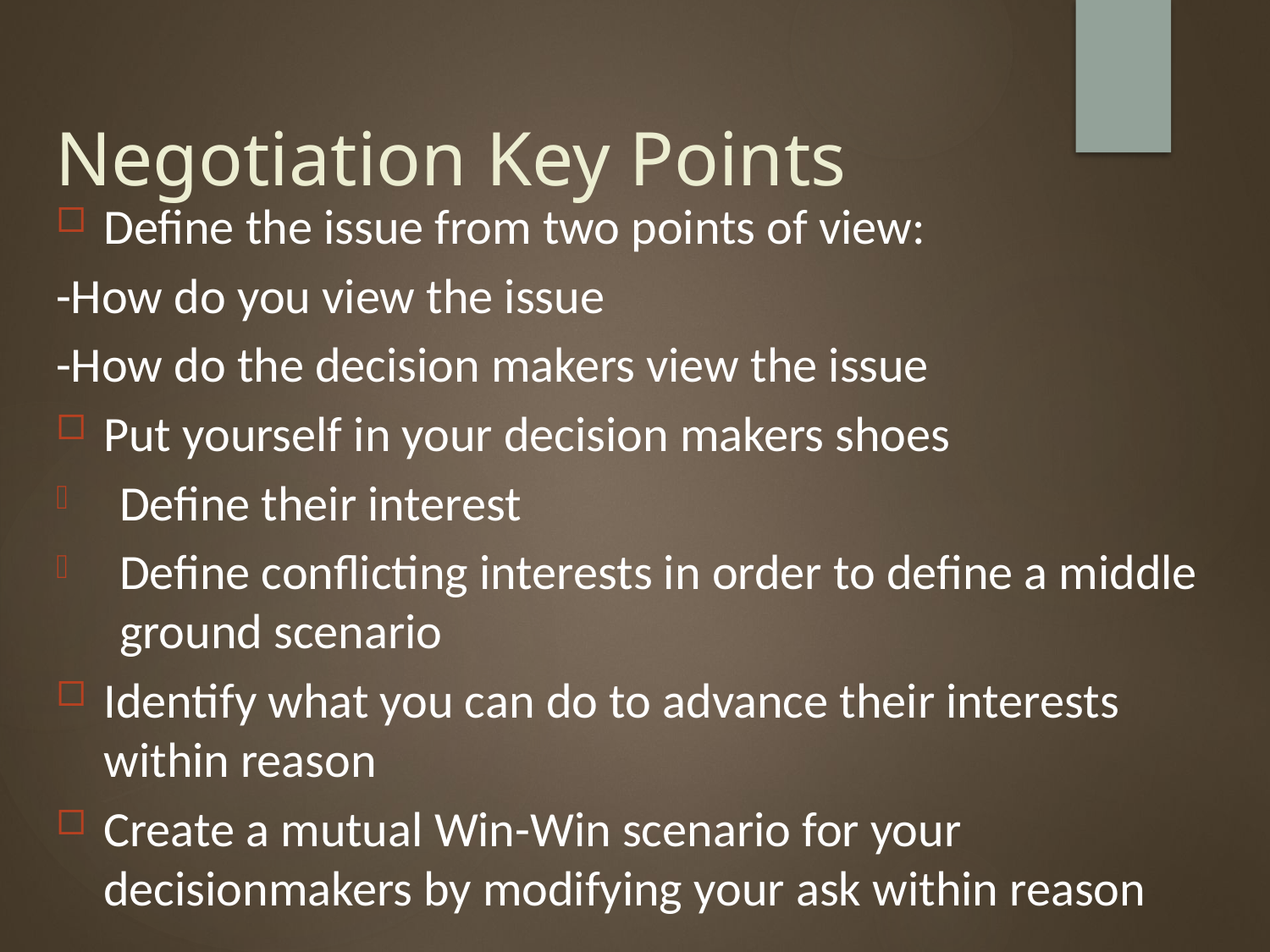

# Negotiation Key Points
Define the issue from two points of view:
-How do you view the issue
-How do the decision makers view the issue
Put yourself in your decision makers shoes
Define their interest
Define conflicting interests in order to define a middle ground scenario
Identify what you can do to advance their interests within reason
Create a mutual Win-Win scenario for your decisionmakers by modifying your ask within reason
