# Supplementary material for: Advancing Advocacy: Implementation of a Child Health Advocacy Curriculum in a Pediatrics Residency Program
Source: MedEdPORTAL. 2020 Feb 14;16:10882. doi: 10.15766/mep_2374-8265.10882 (PMC7062538; doi:10.15766/mep_2374-8265.10882)
Supplement: Supplementary file 1 — A. Lecture 1.pptx B. Lecture 2.pptx C. Lecture 3.ppt D. Lecture 4.pptx E. Workshop 1.pptx F. Workshop 1 Skill Checklist.pdf G. Workshop 2.pptx H. Workshop 3.pptx I. Curriculum Survey.docx [file mep-16-10882-s001.zip › H. Workshop 3.pptx]

## Slide 1
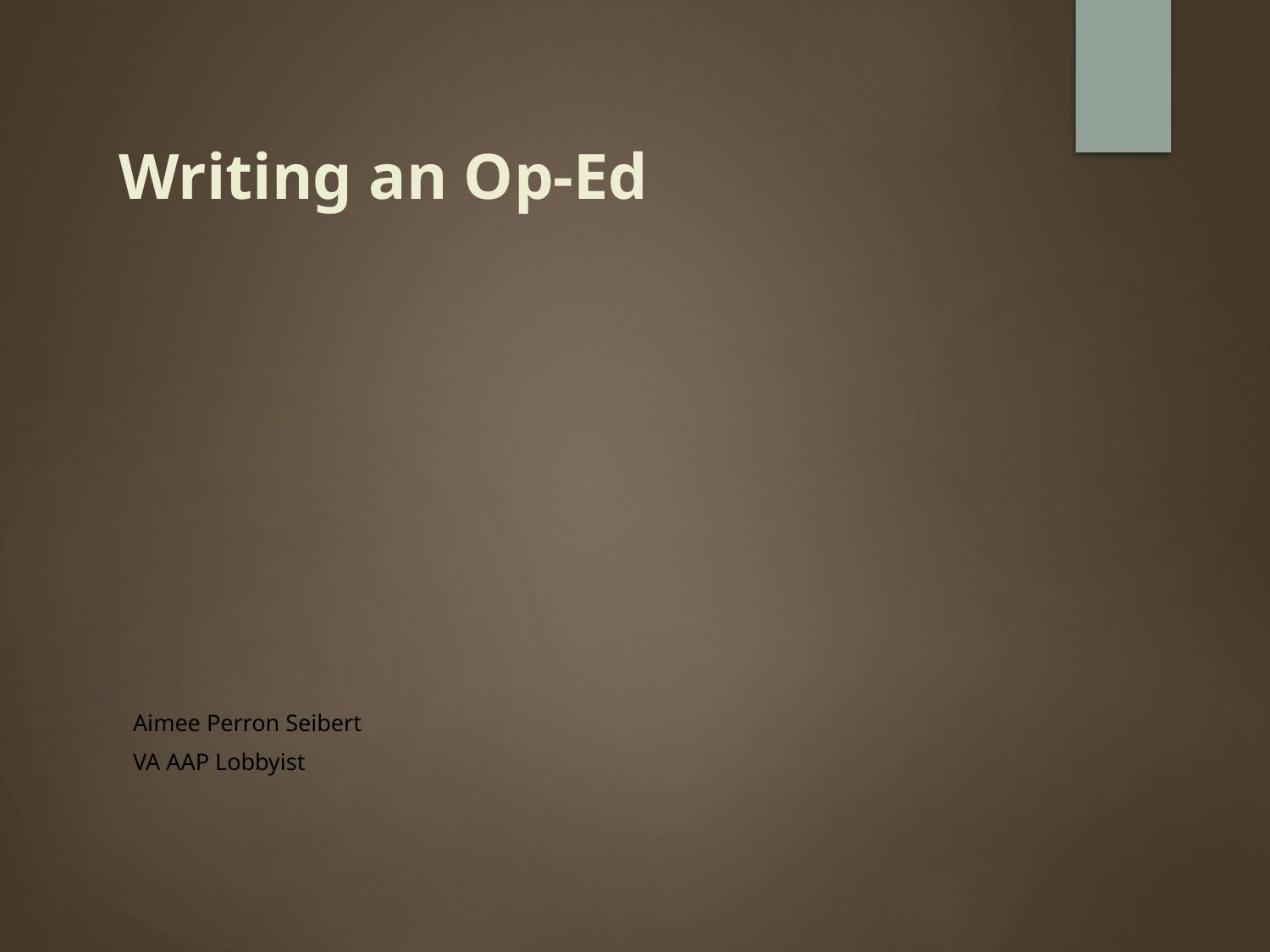

# Writing an Op-Ed
Aimee Perron Seibert
VA AAP Lobbyist

## Slide 2
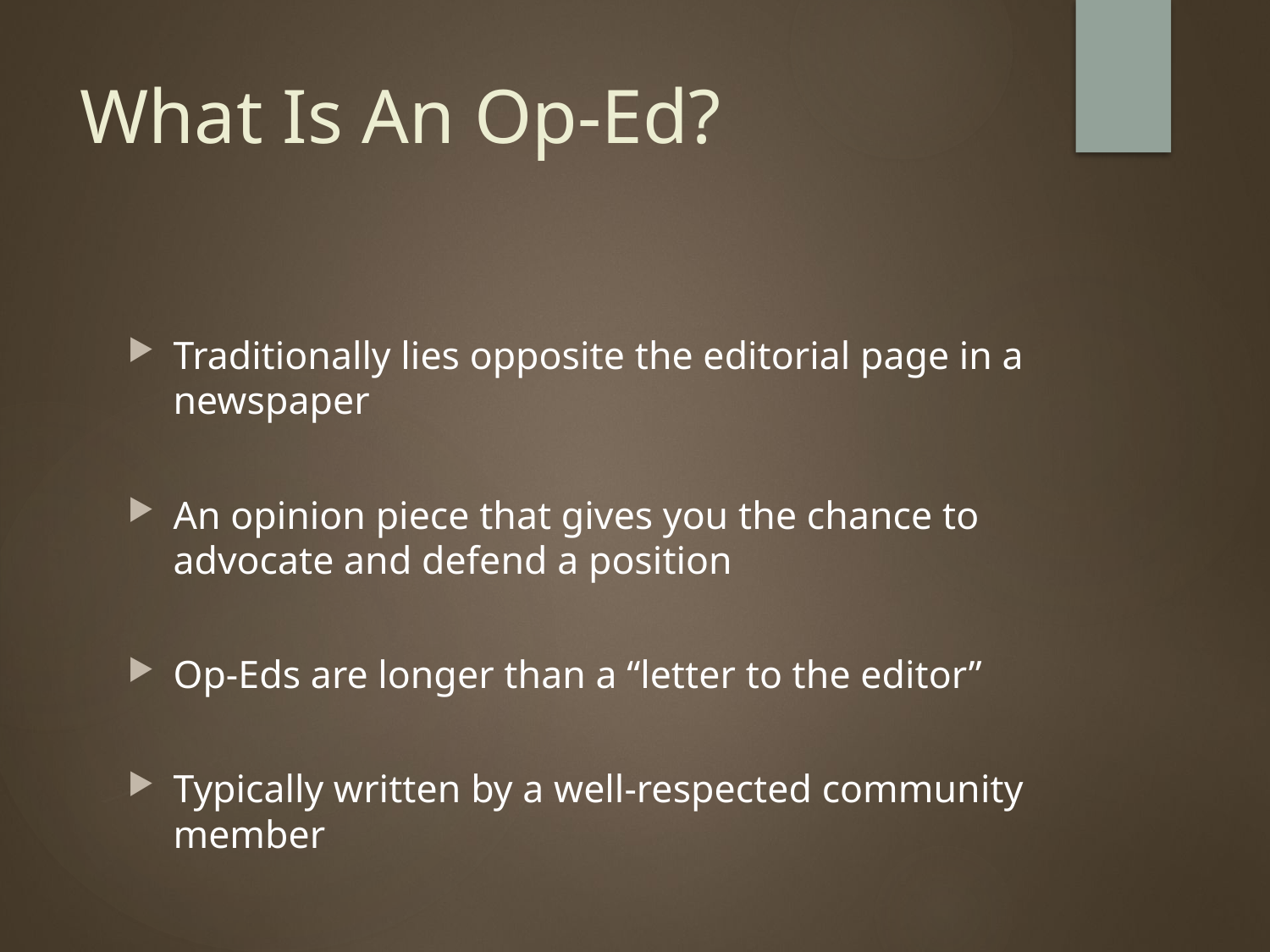

# What Is An Op-Ed?
Traditionally lies opposite the editorial page in a newspaper
An opinion piece that gives you the chance to advocate and defend a position
Op-Eds are longer than a “letter to the editor”
Typically written by a well-respected community member

## Slide 3
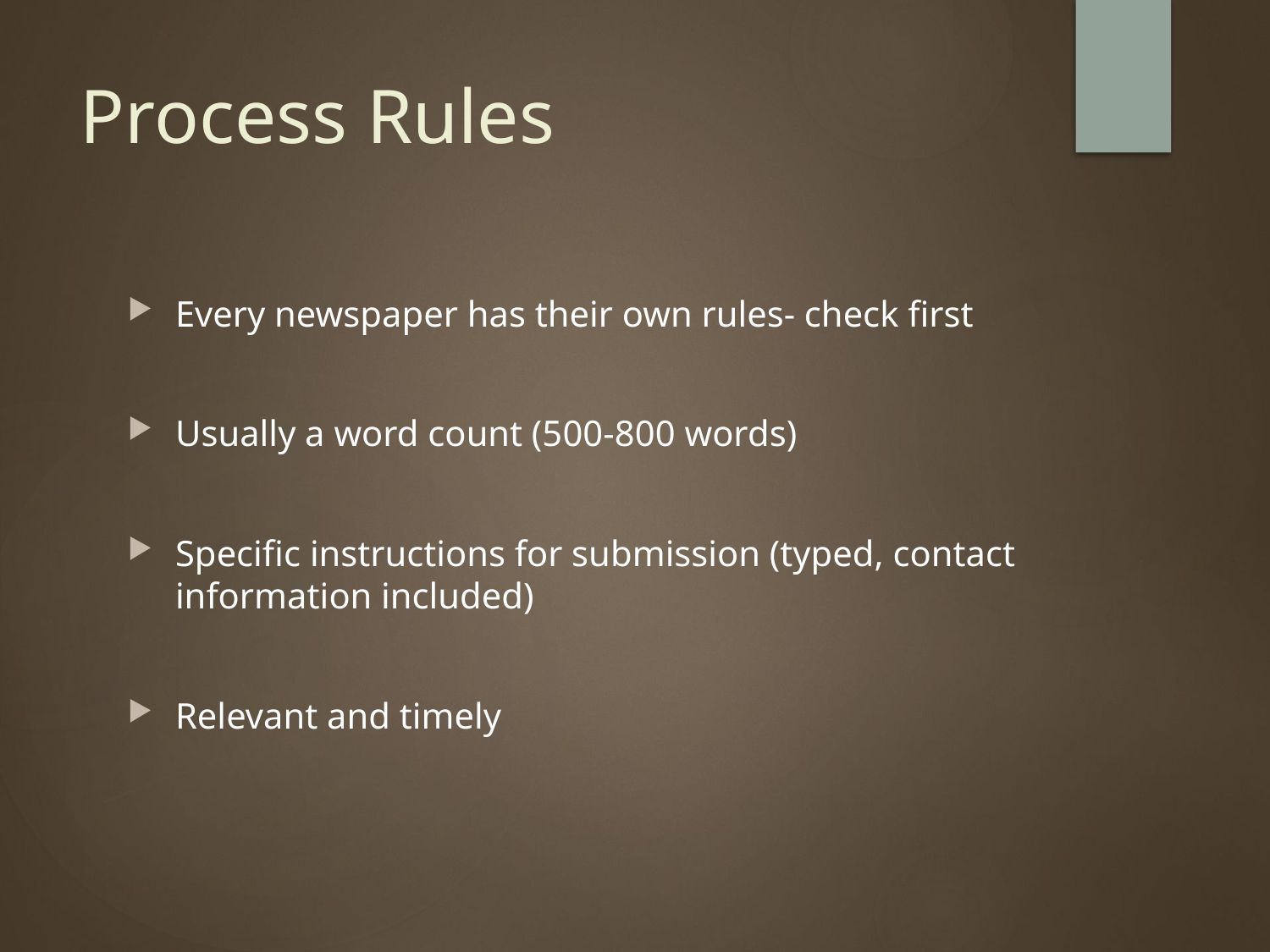

# Process Rules
Every newspaper has their own rules- check first
Usually a word count (500-800 words)
Specific instructions for submission (typed, contact information included)
Relevant and timely

## Slide 4
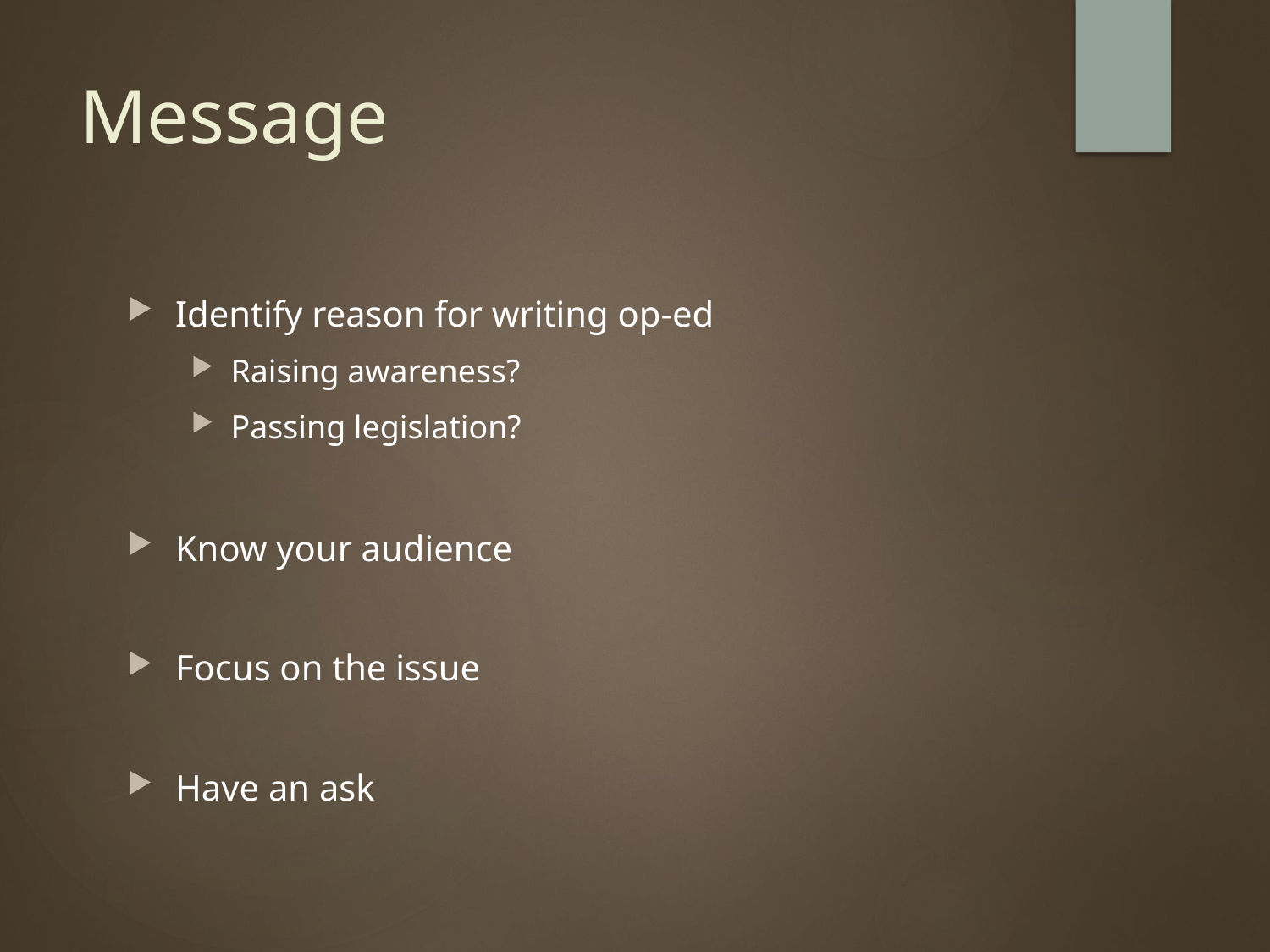

# Message
Identify reason for writing op-ed
Raising awareness?
Passing legislation?
Know your audience
Focus on the issue
Have an ask

## Slide 5
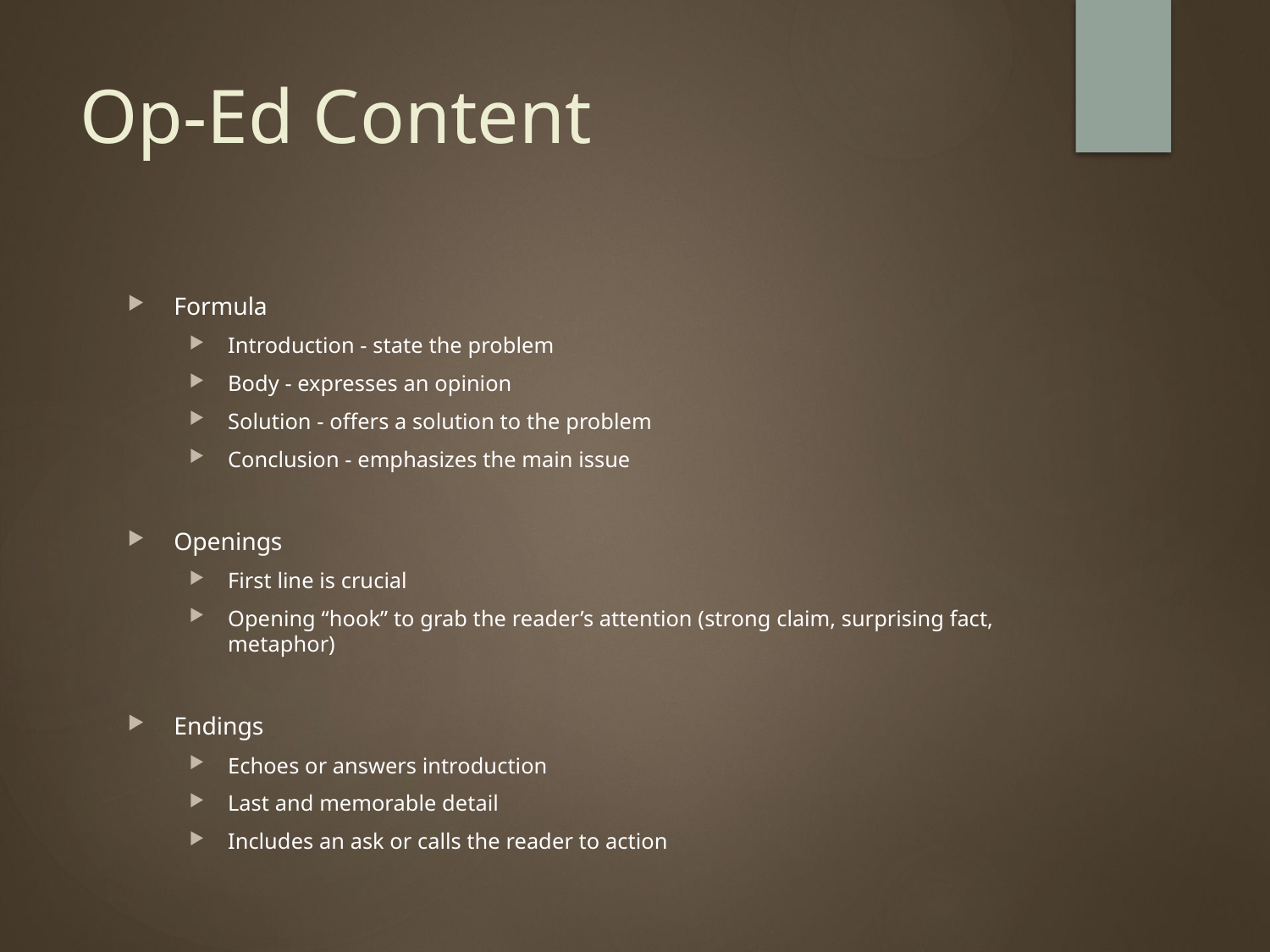

# Op-Ed Content
Formula
Introduction - state the problem
Body - expresses an opinion
Solution - offers a solution to the problem
Conclusion - emphasizes the main issue
Openings
First line is crucial
Opening “hook” to grab the reader’s attention (strong claim, surprising fact, metaphor)
Endings
Echoes or answers introduction
Last and memorable detail
Includes an ask or calls the reader to action

## Slide 6
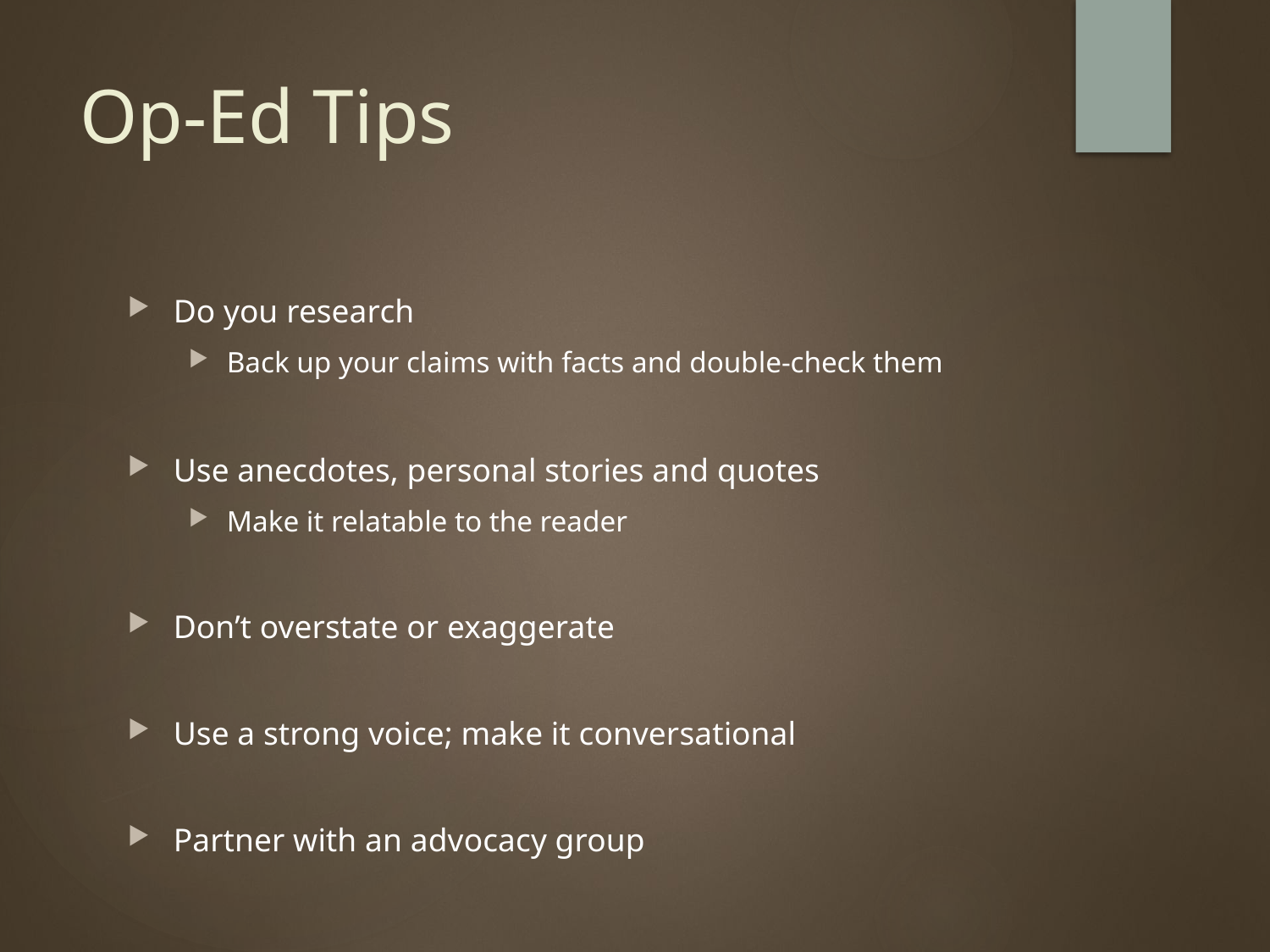

# Op-Ed Tips
Do you research
Back up your claims with facts and double-check them
Use anecdotes, personal stories and quotes
Make it relatable to the reader
Don’t overstate or exaggerate
Use a strong voice; make it conversational
Partner with an advocacy group

## Slide 7
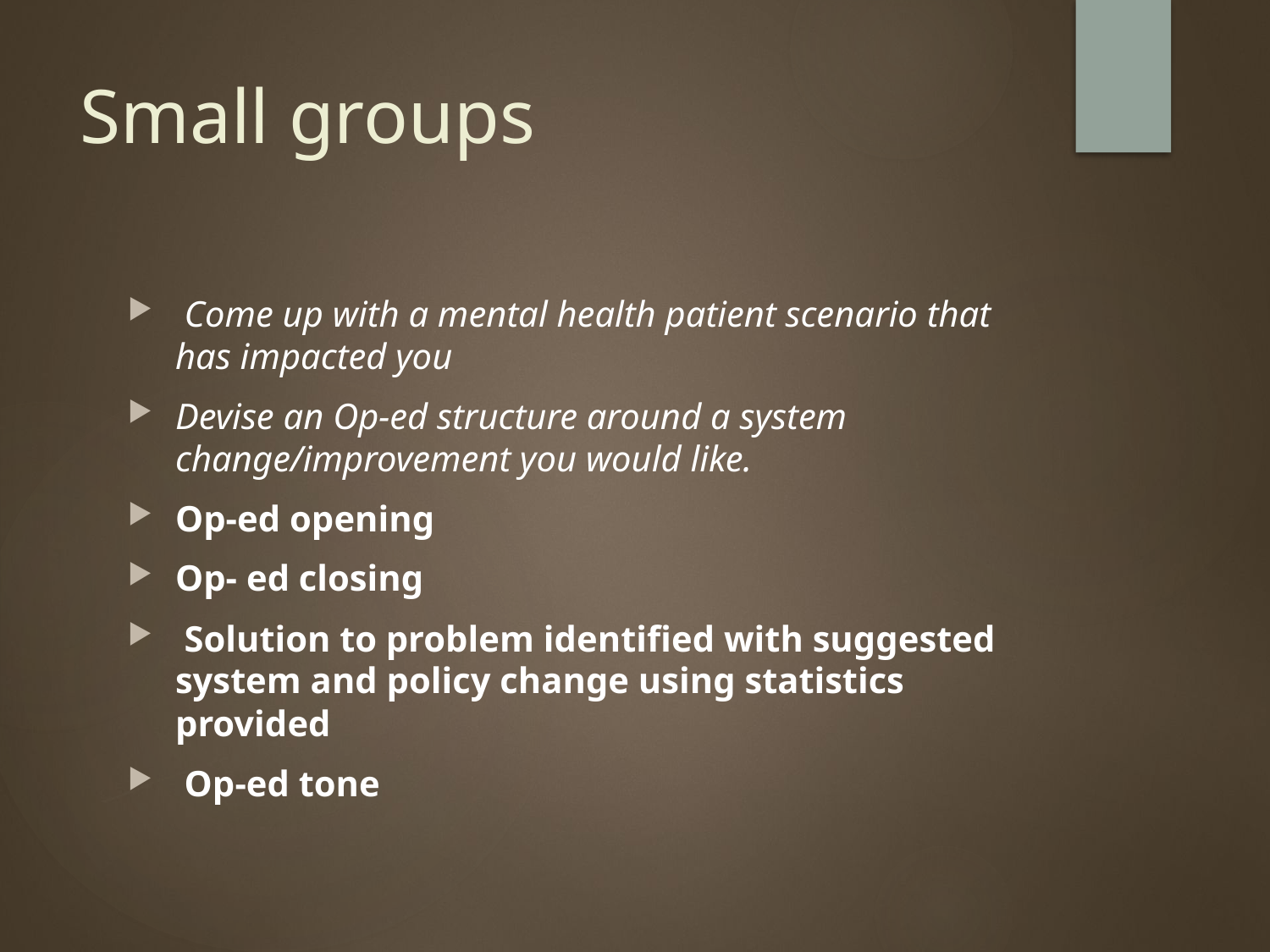

# Small groups
 Come up with a mental health patient scenario that has impacted you
Devise an Op-ed structure around a system change/improvement you would like.
Op-ed opening
Op- ed closing
 Solution to problem identified with suggested system and policy change using statistics provided
 Op-ed tone
