# Supplementary material for: Advancing Advocacy: Implementation of a Child Health Advocacy Curriculum in a Pediatrics Residency Program
Source: MedEdPORTAL. 2020 Feb 14;16:10882. doi: 10.15766/mep_2374-8265.10882 (PMC7062538; doi:10.15766/mep_2374-8265.10882)
Supplement: Supplementary file 1 — A. Lecture 1.pptx B. Lecture 2.pptx C. Lecture 3.ppt D. Lecture 4.pptx E. Workshop 1.pptx F. Workshop 1 Skill Checklist.pdf G. Workshop 2.pptx H. Workshop 3.pptx I. Curriculum Survey.docx [file mep-16-10882-s001.zip › I. Curriculum Survey.docx]

**Advocacy Curriculum Survey**

1. What level of training are you currently in?

Student

PGY 1

PGY 2

PGY 3

PGY 4

1. Have you ever had any formal advocacy training?

Yes

No

1. How likely are you to speak out for a child health issue in the future?

Pre-contemplation

Contemplation

Preparation

Action

1. Have you ever been involved in a community level advocacy?

Yes

No

1. Have you ever been involved in a state level advocacy?

Yes

No

1. Have you ever been involved in a federal level advocacy?

Yes

No

1. How often do you pay attention to current events pertinent to child health?

Daily

Weekly

Monthly

Less than monthly

1. Where do you get your news about pediatric health issues?

Email via American academy of pediatrics federal or state affairs

Local newspaper

American Academy of Pediatrics website

1. Are you a member of any of the following? Mark all that apply.

American Academy of Pediatrics

American Academy of Pediatrics local Chapter

American Medical Association

1. Have you ever attended a local Chapter meeting?

Yes

No

1. Are you interested in learning more about how to effect change at the community, state or

federal level?

5 – Extremely

4 – Very

3 – Moderately

2 – Slightly

1 – Not at all

1. What are your biggest barriers to learning and taking action on issues pertinent to child

health?

Lack of time

I do not understand the legislative process

Intimated by the legislative process

I do not have good awareness regarding pediatric issues in my community

1. How familiar are you with the process in which a bill becomes a law?

5 – Extremely

4 – Very

3 – Moderately

2 – Slightly

1 – Not at all

1. How familiar are you with online resources available for more information on advocacy

training?

5 – Extremely

4 – Very

3 – Moderately

2 – Slightly

1 – Not at all

1. How familiar are you with online resources available for more information on finding your

representative?

5 – Extremely

4 – Very

3 – Moderately

2 – Slightly

1 – Not at all

1. How comfortable are you communicating with your local and state representatives regarding

an issue?

5 – Extremely

4 – Very

3 – Moderately

2 – Slightly

1 – Not at all

1. What would be your preferred method of communicating with your representative?

Letter to your representative

Publishing an op-ed in a newspaper

Interviewing with the media

Meeting your representative in person
